# Organocatalytic and enantioselective Michael reaction between α-nitroesters and nitroalkenes. *Syn*/*anti-*selectivity control using catalysts with the same absolute backbone chirality

**DOI:** 10.3762/bjoc.11.277

**Published:** 2015-12-14

**Authors:** Jose I Martínez, Uxue Uria, Maria Muñiz, Efraím Reyes, Luisa Carrillo, Jose L Vicario

**Affiliations:** 1 Department of Organic Chemistry II, University of the Basque Country (UPV/EHU), P.O. Box 644, 48080 Bilbao, Spain

**Keywords:** asymmetric diastereodivergent, enantioselective, Michael addition, nitroalkenes, nitroesters, organocatalysis, squaramides

## Abstract

The asymmetric and catalytic Michael reaction between α-nitroesters and nitroalkenes has been studied in the presence of two bifunctional catalysts both containing the same absolute chirality at the carbon backbone. The reaction performed in similar conditions allows us to control the *syn* or *anti* selectivity of the Michael adduct obtaining good yields and high enantiocontrol in all cases.

## Introduction

The absolute stereochemistry of a molecule has a paramount influence on the properties that this compound will have when interacting with biological systems [1]. As a consequence, the last decades have witnessed an enormous progress directed toward the development of synthetic methodology for the preparation of chiral molecules as single enantiomers with a well-defined three-dimensional arrangement. In this scenario, asymmetric catalysis arises as a key methodology for chemical production of enantiomerically enriched chiral compounds in terms of atom economy and reduced waste generation [2–6]. Nowadays, many very effective methodologies exist that allow the formation of a chiral compound as a single enantiomer. However, and despite the advances made in the field, an important challenge arises when a molecule containing multiple stereocentres has to be prepared because the access to all possible stereoisomers is not usually straightforward. While obtaining one or other mirror image of the target molecule can also be easily achieved by selecting the correct enantiomer of the catalyst, the relative configuration is typically governed by intrinsic factors associated to the mechanistic profile of the reaction and very often the formation of the major diastereoisomer is determined from the very beginning of the reaction and therefore access to any stereoisomer at will from the same set of starting materials with full absolute and relative stereocontrol is not trivial. Previous reports show that the diastereoselection can be directed by different approaches that include the modification of reaction conditions [7–9], the incorporation of additives or co-catalysts [10–13], the modification of some structural features of the substrate [14–15], the use of two catalysts that are structurally different to each other and that can also operate independently through a single transition state with minimal matched/mismatched interactions [16–22] or finally the concurrent use of two cycle-specific catalysts, in which each of them is exclusively involved in the formation of one stereocentre and therefore has to overcome the stereochemical bias exerted by the stereocentres generated in the previous steps [23–26].

**Scheme 1:**
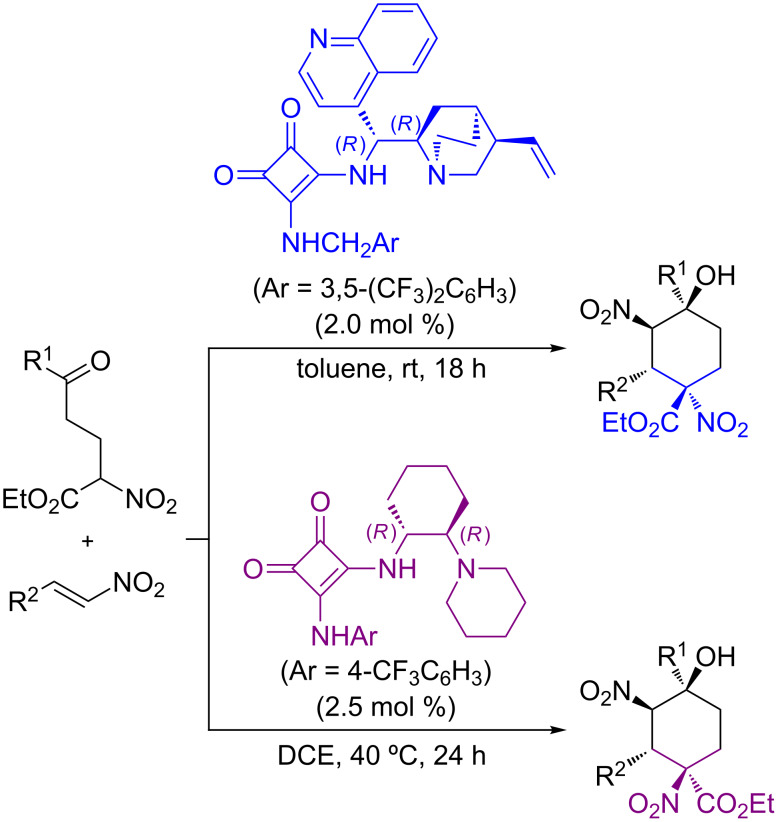
Diastereodivergent cascade Michael/Michael reaction using catalysts with the same absolute chirality reported in our group.

In this context, we have recently reported a catalytic and enantioselective Michael/Michael cascade reaction in which α-nitro-δ-ketoesters react with nitroalkenes to provide densely functionalized cyclohexanes in excellent yield and stereoselectivity (Scheme 1) [27]. This reaction made use of bifunctional tertiary amine/squaramide catalysts [28–32] and, interestingly, we found that catalysts containing the same backbone chirality provided different diastereoisomers, thus allowing the development of a diastereodivergent process.

In this report, we wish to present the extension of this behaviour to the Michael reaction between α-substituted nitroacetates and nitroalkenes. The Michael reaction is regarded as a fundamental tool for the formation of C–C bonds during the synthesis of complex molecules [33–35], in particular, being able to control both the simple and the facial stereoselection of those versions in which two stereocentres are generated in the reaction become of special utility to synthetic chemists. In this case, using two different bifunctional tertiary amine/squaramide organocatalysts [36–61] with the same absolute chirality, the stereochemical outcome of the reaction can be controlled to provide the corresponding Michael adducts with two stereocentres, one of them being quaternary, and allowing the preparation of the desired diastereoisomer at will.

## Results and Discussion

**Scheme 2:**
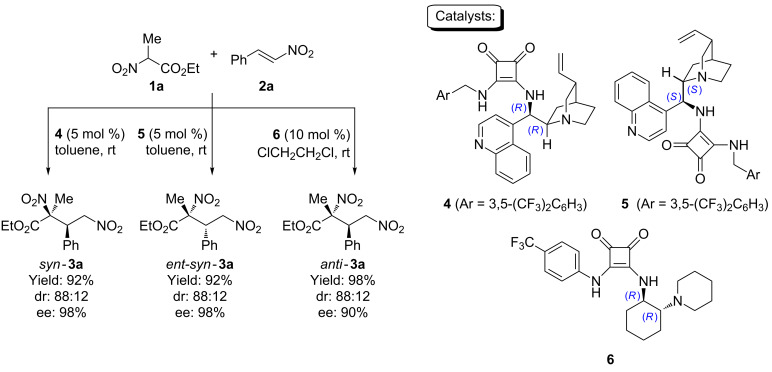
Diastereodivergent enantioselective Michael reaction using ethyl 2-nitropropanoate and β-nitrostyrene as model substrates.

We started our work by first applying the conditions optimized for our recently reported cascade process to the reaction between ethyl 2-nitropropanoate (**1a**) and β-nitrostyrene (**2a**). As it can be seen in Scheme 2, the use of catalyst **4** led to the formation of the *syn*-diastereoisomer *syn*-**3a** in excellent yield, an acceptable 88:12 dr and 98% ee. As expected, the use of the pseudoenantiomeric catalyst **5** provided the corresponding enantiomer *ent*-*syn*-**3a** with similar results. Moving to cyclohexanediamine-based catalyst **6** resulted in the same behaviour as observed in our previous report, isolating the other possible diastereoisomer *anti*-**3a** in good yield, 88:12 dr and 90% ee.

Once we had confirmed the good performance of the reaction in this intermolecular Michael reaction, we proceeded to evaluate the scope of this transformation in order to establish whether this method could be useful and general for a variety of nitroalkene Michael acceptors and nitroacetate donors. For this reason, we initially studied the reaction using squaramide **4** as catalyst that leads to the formation of *syn*-**3** adducts. In this sense, and as it can be seen in Table 1, the reaction performed excellently in almost all the cases studied. In particular, when the reaction was conducted using nitrostyrene derivatives as Michael acceptors, the corresponding adducts **3a–k** were isolated cleanly, in high yields, diastereo- and enantioselectivities regardless the electronic nature of the aromatic substituent of the nitrostyrene reagent. Indeed, the reaction using nitrostyrenes containing electron-donating groups at any of the position of the aryl ring led to the formation of the corresponding adducts (*syn*-**3a–f**) in excellent yield, good diastereoselectivities and enantiomeric excesses over 95% (Table 1, entries 2–6).

**Table 1:** Enantio- and diastereoselective Michael reaction between nitroesters **1** and nitroalkenes **2** catalysed by **4**.^a^

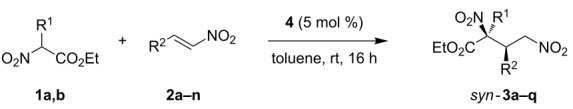						

Entry	R^1^	R^2^	Product	Yield^b^	dr^c^	ee (%)^d^

1	Me	Ph	*syn*-**3a** [27]	92	88:12	98
2	Me	4-MeC_6_H_4_	*syn*-**3b**	85	89:11	97
3	Me	2-MeOC_6_H_4_	*syn*-**3c**	79	82:18	96
4	Me	3-MeOC_6_H_4_	*syn*-**3d**	92	88:12	96
5	Me	4-MeOC_6_H_4_	*syn*-**3e**	86	87:13	>99
6	Me	4-BnOC_6_H_4_	*syn*-**3f**	85	86:14	97
7	Me	2-ClC_6_H_4_	*syn*-**3g**	80	88:12	98
8	Me	3-ClC_6_H_4_	*syn*-**3h**	92	86:14	97
9	Me	4-ClC_6_H_4_	*syn*-**3i**	97	88:12	97
10	Me	2-BrC_6_H_4_	*syn*-**3j**	75	85:15	97
11	Me	4-BrC_6_H_4_	*syn*-**3k**	92	87:13	97
12	Me	2-furyl	*syn*-**3l**	80	86:14	93
13	Me	2-thienyl	*syn*-**3m**	86	93:7	96
14	Me	(MeO)_2_CH	*syn*-**3n**	96	80:20	96
15	Et	Ph	*syn*-**3o**	92	86:14	98
16	Et	4-MeOC_6_H_4_	*syn*-**3p**	88	84:16	97
17	Et	4-BrC_6_H_4_	*syn*-**3q**	78	78:22	95

^a^All reactions were carried out on a 0.1 mmol scale of **1** and **2** in toluene (1 M) at room temperature using 5.0 mol % of **4** as catalyst. ^b^Yield of pure Michael adducts as mixture of diastereoisomers after flash column chromatographic purification. ^c^Determined by ^1^H NMR analysis of the crude reaction mixture. ^d^Determined by HPLC on a chiral stationary phase (see experimental part in Supporting Information File 1 for details).

When nitrostyrenes containing electron-withdrawing groups were tested, the reaction proceeded equally well (Table 1, entries 7–11), although a slight decrease in the yield was observed for those cases in which the substituent was placed at the *ortho*-position (Table 1, entries 7 and 10). Heteroaromatic substituents at the nitroalkene reagent were also tested and those also reacted very efficiently, leading to the formation of adduct *syn*-**3l**–**m** in high yield and good stereoselection (Table 1, entries 12 and 13). Functionalized nitroalkene **2n** also performed well in the reaction, providing the corresponding addition product *syn*-**3n** in good yield and high diastereo- and enantioselectivity (Table 1, entry 14). Disappointingly, when we tested β-alkyl-substituted nitroalkenes (R^2^ = iPr or *n-*Pr) we could not identify the formation of the desired addition product, and a complex mixture of products was obtained as a consequence of the very likely decomposition of these rather unstable nitroalkene reagents. Finally, we also surveyed the use of a nitroacetate donor with a bulkier substituent such as **1b** which also performed very well in the reaction with *trans*-β-nitrostyrene (**2a**), *para*-methoxy-*trans*-β-nitrostyrene (**2e**) or *para*-bromo-*trans*-β-nitrostyrene (**2k**), yielding the corresponding products *syn*-**3o–q** with comparable results to those obtained with Michael donor **1a** (Table 1, entries 15–17).

Next, we proceeded to evaluate the scope of the reaction leading to diastereomeric adducts *anti*-**3** (Table 2) using bifunctional cyclohexanediamine/squaramide catalyst **6** which, as mentioned before, is based on a chiral backbone with the same absolute chirality as **4**. The reaction conditions used for this reaction were those already observed to be optimal in our previous work for the cascade Michael/Henry reaction using this catalyst, that involved changing the solvent to 1,2-dichloroethane [27]. As it happened in the previous case, we could observe that the reaction performed well in all cases tested and, in general, with a similar level of chemical efficiency and stereocontrol, although in comparison with the **4**-catalyzed version, enantioselectivities are normally around 5–10% lower if we analyse case by case. As it can be seen in Table 2, nitrostyrenes containing electron-withdrawing or electron-donating groups at the aromatic moiety performed similarly well in all cases, regardless the position of the substituents (Table 2, entries 1–11) and also heteroaryl moieties were well tolerated in the reaction (Table 2, entries 12 and 13). In the same line, functionalized nitroalkene **2n** could also be successfully used in the reaction leading to adduct *anti*-**3n** in good yield, diastereo- and enantioselectivity (Table 2, entry 14). The use of bulkier ethyl 2-nitrobutanoate (**1b**) as Michael donor also led to good results for three representative nitroalkenes (Table 2, entries 15–17), although in the case of nitroalkene **2e** a somewhat lower enantiomeric excess was obtained. Finally, and as it happened in the reaction catalyzed by **4**, β-alkyl-substituted nitroalkenes were not suitable substrates for the reaction, probably due to their tendency to polymerize under the reaction conditions.

**Table 2:** Enantio- and diastereoselective Michael reaction between nitroesters **1** and nitroalkenes **2** catalysed by **6**.^a^

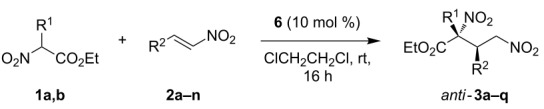						

Entry	R^1^	R^2^	Product	Yield^b^	dr^c^	ee (%)^d^

1	Me	Ph	*anti*-**3a** [27]	98	88:12	90
2	Me	4-MeC_6_H_4_	*anti*-**3b** [62]	94	85:15	84
3	Me	2-MeOC_6_H_4_	*anti*-**3c**	95	90:10	86
4	Me	3-MeOC_6_H_4_	*anti*-**3d**	92	86:14	86
5	Me	4-MeOC_6_H_4_	*anti*-**3e** [62]	92	86:14	78
6	Me	4-BnOC_6_H_4_	*anti*-**3f**	90	86:14	81
7	Me	2-ClC_6_H_4_	*anti*-**3g** [62]	94	93:7	91
8	Me	3-ClC_6_H_4_	*anti*-**3h**	92	85:15	84
9	Me	4-ClC_6_H_4_	*anti*-**3i** [62]	91	83:17	84
10	Me	2-BrC_6_H_4_	*anti*-**3j** [62]	97	93:7	90
11	Me	4-BrC_6_H_4_	*anti*-**3k** [62]	93	83:17	84
12	Me	2-furyl	*anti*-**3l** [62]	94	80:20	80
13	Me	2-thienyl	*anti*-**3m**	98	87:13	80
14	Me	(MeO)_2_CH	*anti*-**3n**	96	76:24	84
15	Et	Ph	*anti*-**3o** [62]	94	87:13	90
16	Et	4-MeOC_6_H_4_	*anti*-**3p**	94	87:13	64
17	Et	4-BrC_6_H_4_	*anti*-**3q**	94	88:12	84

^a^All reactions were carried out on a 0.1 mmol scale of **1** and **2** in 1,2-dichloroethane (1 M) at room temperature using 10.0 mol % of **6** as catalyst. ^b^Yield of pure Michael adducts as mixture of diastereoisomers after flash column chromatographic purification. ^c^Determined by ^1^H NMR analysis of the crude reaction mixture. ^d^Determined by HPLC on a chiral stationary phase (see experimental part in Supporting Information File 1 for details).

**Figure 1:**
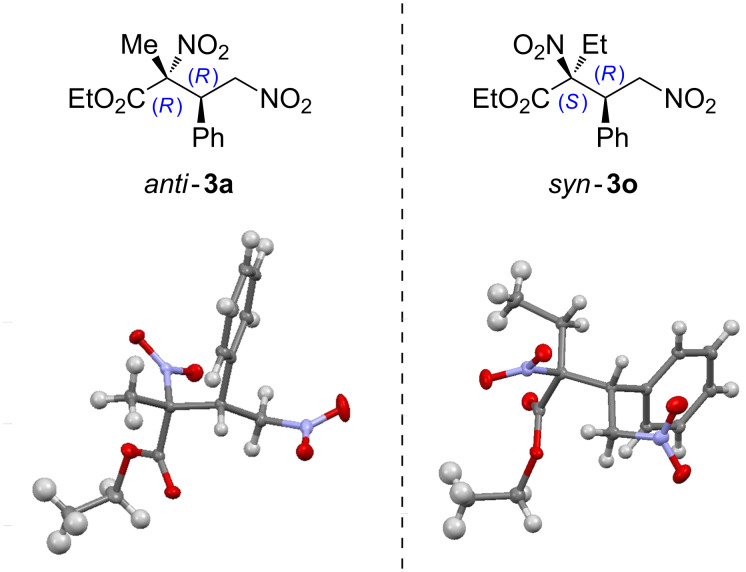
ORTEP diagrams for *anti*-**3a** and *syn*-**3o** respectively.

The absolute configuration was determined by X-ray analysis of products *anti*-**3a** and *syn*-**3o** for which suitable crystals could be obtained (Figure 1). The crystallographic analysis gave a (2*R*,3*R*) configuration for *anti*-**3a** [27] and a (2*S*,3*R*) for *syn*-**3o** [63] and this stereochemical assignment was extended to all other adducts prepared assuming an identical mechanistic pathway.

**Scheme 3:**
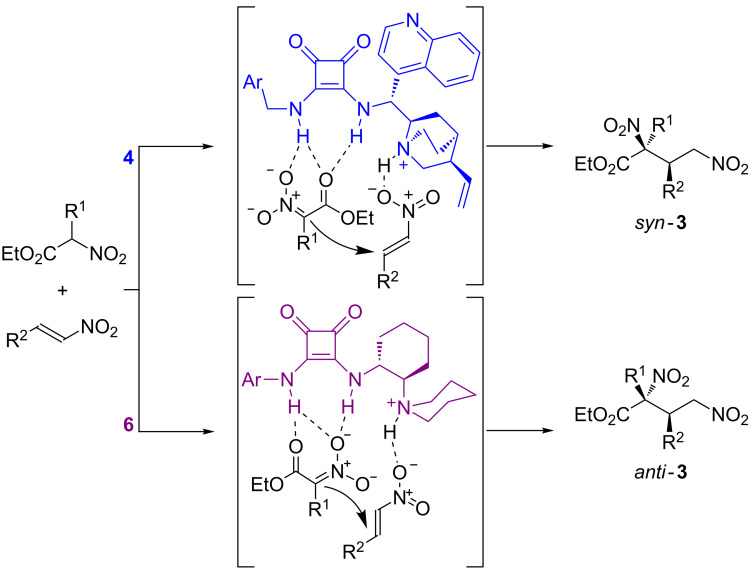
Proposed models to explain the stereochemical outcome of the reaction.

This configuration is also in agreement with a transition state for the Michael reaction such as the one proposed in Scheme 3 [64]. This involves the activation of the nucleophile by the squaramide moiety of the bifunctional catalysts **4** and **6** through the formation of multiple H-bonding interactions and the simultaneous interaction between the nitroalkene acceptor and the ammonium salt moiety, the latter being generated after the initial deprotonation of the pronucleophile. The different possibilities offered by the two catalysts **4** and **6** to form geometrically different H-bonded complexes with the nitroacetate enolate would account for the different simple diastereoselection observed in each case, in which the nitronate moiety exposes a different reactive prochiral face. These results are in good agreement with our previously reported work in which DFT calculations also showed that the difference in the steric bulk of the nitrogen substituents of the Brønsted basic site of the catalysts (the quinuclidine moiety in catalyst **4** and the piperidine scaffold in catalyst **6**) are the key parameters influencing this different arrangement for the nitroacetate pronucleophile [27].

## Conclusion

We have developed an asymmetric catalytic diastereodivergent route for the synthesis of 2,4-dinitro esters taking advantage of the Michael addition of nitroalkenes and using two different bifunctional catalysts derived from cinchona alkaloids (catalyst **4**) or cyclohexadiamine (catalyst **6**). These catalysts, both with the same absolute backbone chirality, allow us to control the *syn* or *anti* selectivity obtaining the final products in good to excellent yields and with high enantioselectivity.

## Supporting Information

File 1 Experimental details, analytical data, NMR spectra and HPLC traces of all compounds prepared.
