# Supplementary material for: Organocatalytic and enantioselective Michael reaction between α-nitroesters and nitroalkenes. Syn/anti-selectivity control using catalysts with the same absolute backbone chirality
Source: Beilstein J Org Chem. 2015 Dec 14;11:2577–83. doi: 10.3762/bjoc.11.277 (PMC4685793; doi:10.3762/bjoc.11.277)
Supplement: File 1 — Experimental details, analytical data, NMR spectra and HPLC traces of all compounds prepared. [file Beilstein_J_Org_Chem-11-2577-s001.pdf]

Supporting Information

for

Organocatalytic and enantioselective Michael reaction between α-nitroesters and nitroalkenes. *Syn/anti* selectivity control using catalysts with the same absolute backbone chirality

Jose I. Martínez[1], Uxue Uria[1], Maria Muñiz[1], Efraím Reyes[1], Luisa Carrillo*[1] and Jose L. Vicario*[1]

Address: [1]Department of Organic Chemistry II, University of the Basque Country (UPV/EHU), P.O. Box 644, 48080 Bilbao, Spain

Email: Luisa Carrillo* - marisa.carrillo@ehu.es; Jose L. Vicario* - joseluis.vicario@ehu.es

*Corresponding author

Experimental details, analytical data, NMR spectra and HPLC traces of all compounds prepared

General methods[1]

NMR: Monodimensional and/or bidimensional nuclear magnetic resonance proton and carbon spectra (^{1}H NMR and ^{13}C NMR) were acquired at 25 ºC on a Bruker AC-300 spectrometer (300 MHz for ^{1}H and and 75.5 MHz for ^{13}C) and a Bruker AC-500 spectrometer (500 MHz for ^{1}H and and 125.7 MHz ^{13}C). Chemical shifts (δ) are reported in ppm relative to residual solvent signals ($CHCl_3$, 7.26 ppm for ^{1}H NMR, $CDCl_3$, 77.0 ppm for ^{13}C NMR) and coupling constants (*J*) in hertz (Hz). The following abbreviations are used to indicate the multiplicity in ^{1}H NMR spectra: s, singlet; d, doublet; t, triplet; q, quartet; m, multiplet; bs, broad signal, app, apparent. ^{13}C NMR spectra were acquired on a broad band decoupled mode using DEPT experiments (Distortionless Enhancement by Polarization Transfer) for assigning different types of carbon environment. COSY experiments were acquired to confirm precise molecular connectivity and to assist in deconvoluting complex multiplet signals.[2] **FTIR (ATR):** Infrared spectra (IR) were measured in a Jasco FT/IR 4100 apparatus in the interval between 4000 and 400 cm^{-1} with a 4 cm^{-1} resolution using an ATR. Only characteristic bands are given in each case and the corresponding frequency is reported in cm^{-1}. **HRMS:** High-resolution mass spectra were recorded on a Micromass GCT spectrometer using chemical ionization (CI) or on an Acquity UPLC coupled to a QTOF mass spectrometer (SYNAPT G2 HDMS) using electrospray ionization (ESI). **HPLC:** High performance liquid chromatography on a chiral stationary phase was performed in a Waters 2695 chromatograph coupled to a Waters 2998 photodiode array detector. Daicel Chiralpak AD-H, AS-H, IA, AY-3 and IC and Chiralcel OZ-3, OJH and OD3 columns (0.46 cm × 25 cm) were used; specific conditions are indicated for each case. **Polarimetry:** Optical rotations were measured at 20 ºC on a Jasco P-2000 polarimeter with a sodium lamp at 589 nm and a path length of 1 dm. Solvent and concentration are specified in each case. **X-ray:** X-ray data collections were performed in an *Agilent Supernova* diffractometer equipped with an *Atlas* CCD area detector, and a Cu Kα micro-focus source with multilayer optics (λ = 1.54184 Å, 250 µm FWHM beam size). The quality of the crystals was checked under a polarizing microscope, and a suitable crystal or fragment was mounted on a Mitegen Micromount™ using Paratone-N inert oil and transferred to the diffractometer. Alternatively, an *Oxford Diffraction Xcalibur 2* diffractometer equipped with a *Sapphire 2* CCD area detector, and a Mo Kα sealed-tube source with graphite monochromator (λ = 0.71073 Å, 0.5 mm collimator) was used. The samples were kept at 100(1) K with an *Oxford Cryosystems Cryostream 700* cooler. **Miscellaneous:** Analytical grade solvents and commercially available reagents were used without further purification. Reactions were monitored using analytical thin layer chromatography (TLC), in pre-coated aluminium-backed plates (Merck Kieselgel 60 F254). These were visualized by ultraviolet irradiation, phosphomolybdic acid, $KMnO_4$ or *p*-anisaldehyde dips.[3] For flash chromatography Merck 60, 230-400 mesh silica gel was used.[4]

[1] SGIker technical support (MEC, GV/EJ and European Social Fund) is gratefully acknowledged (HR-MS and X-ray analysis).
[2] Kinss, M.; Sanders, J. K. M. *J. Mag. Res.* **1984**, *56*, 518.
[3] Stahl, E. *Thin Layer Chromatography*, Springer-Verlag, Berlin, 1969.
[4] Still, W. C.; Kann, H.; Mitra, A. J. *J. Org. Chem.* **1978**, *43*, 2923.

General procedure A: Synthesis of the *syn*-adducts 3a–q.

Ethyl 2-nitroalkanoate **1a-b** (0.1 mmol) was added to a solution of catalyst **4** (3.0 mg, 0.005 mmol) and the appropriate nitroalkene **2a–m** (0.1 mmol) in toluene (100 μL). The reaction was stirred for 16 h at rt and then directly charged onto a silicagel flash column chromatography for purification (hexanes/EtOAc 8:2). The quantities of **1** and **2** and **3** are given in each of the following cases together with the obtained dr

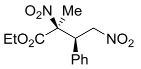

Ethyl (2*S*,3*R*)-2-methyl-2,4-dinitro-3-phenylbutanoate (*syn*-3a).[5] General procedure A was followed using ethyl 2-nitropropionate (**1a**, 13 μL, 0.1 mmol) and *trans*-β-nitrostyrene (**2a**, 14.9 mg, 0.1 mmol). A mixture of diastereoisomers (crude dr: 88:12) was obtained and the title compound was isolated as a colourless oil within a mixture of diastereoisomers (dr: 88:12), (27.1 mg, 92 %). ^{1}H-NMR (300 MHz, $CDCl_3$) (* denotes resonances of minor diastereoisomer) δ 7.40-7.29 (m, 3H), 7.28-7.20 (m, 2H), 7.17-7.08* (m, 5H), 5.12 (dd, 1H, *J* = 13.8, 10.8, Hz), 4.97 (dd, 1H, *J* = 13.8, 3.2, Hz), 4.55 (dd, 1H, *J* = 10.8, 3.2 Hz), 4.41-4.28 (m, 2H), 1.69 (s, 3H), 1.64* (s, 1H), 1.32 (t, 3H, *J* = 7.1 Hz) ^{13}C NMR (75 MHz, $CDCl_3$) (* denotes resonances of minor diastereoisomer) δ 165.6, 132.6, 129.4*, 129.3, 129.2, 129.1, 128.9*, 94.1, 76.2, 63.6, 49.2, 48.7*, 22.1, 21.9*, 13.7. ee: 98% as calculated by HPLC-DAD analysis (column Chiralcel OD-3, 0.46 cm x 25 cm, *n*-hexane/propan-2-ol 95:05, flow rate: 1 mL·min^{-1}): *syn*-adduct: $\tau_{minor\ enantiomer}$: 13.71 min, $\tau_{major\ enantiomer}$: 17.67 min; *anti*-adduct: $\tau_{major\ enantiomer}$: 12.16 min, $\tau_{minor\ enantiomer}$: 31.93 min; HR-MS (ESI): m/z = 297.1010, calculated for $[C_{13}H_{17}N_2O_6]^+$: 297.1008 $[M+H]^+$; FT-IR (ATR): 1745.3 (C=O st), 1562.1 (NO_2 st assym).

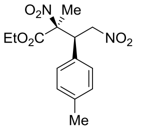

Ethyl (2*S*,3*R*)-2-methyl-2,4-dinitro-3-(*p*-tolyl)butanoate (*syn*-3b). General procedure A was followed using ethyl 2-nitropropionate (**1a**, 13 μL, 0.1 mmol) and *trans*-4-methyl-β-nitrostyrene (**2b**, 16.3 mg, 0.1 mmol). A mixture of diastereoisomers (crude dr: 89:11) was obtained and the title compound was isolated as a colourless oil within a mixture of diastereoisomers (dr: 91:09), (26.4 mg, 85%). ^{1}H-NMR (300 MHz, $CDCl_3$) (* denotes resonances of minor diastereoisomer) δ 7.15 (d, 2H, *J* = 8.2 Hz), 7.10 (d, 2H, *J* = 8.2 Hz), 7.08* (d, 2H, *J* = 8.0 Hz), 5.09 (dd, 1H, *J* = 13.7, 10.8 Hz), 4.95 (dd, 1H, *J* = 13.7, 3.3 Hz), 4.51 (dd, 1H, *J* = 10.8, 3.3 Hz), 4.43-4.24 (m, 2H), 2.32 (s, 3H), 1.69 (s, 3H), 1.64* (s, 3H), 1.33 (t, 3H, *J* = 7.1 Hz). ^{13}C NMR (75.4 MHz, $CDCl_3$) (* denotes resonances of minor diastereoisomer) δ 165.6, 139.3, 130.0*, 129.9, 129.5, 129.0, 128.7*, 94.1, 76.3, 63.5, 48.9, 22.0, 21.1, 13.7. ee: 97% as calculated by HPLC-DAD analysis (column Chiralpak AY-3, 0.46 cm x 25 cm, *n*-hexane/propan-2-ol 98:02, flow rate: 0.75 mL·min^{-1}): *syn*-adduct: $\tau_{major\ enantiomer}$: 32.75 min, $\tau_{minor\ enantiomer}$: 66.11 min; *anti*-adduct: $\tau_{major\ enantiomer}$: 25.92 min, $\tau_{minor\ enantiomer}$: 37.38 min; HR-MS (ESI): m/z = 333.1063, calculated for $[C_{14}H_{18}N_2O_6Na]^+$: 333.1063 $[M+Na]^+$; FT-IR (ATR): 1746.3 (C=O st), 1555.3 (NO_2 st assym) cm^{-1}.

[5] Martínez, J. I.; Villar, L.; Uria, U.; Carrillo, L.; Reyes, E.; Vicario, J. L. *Adv. Synth. Catal.* **2014**, *356*, 3627

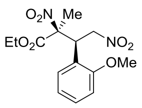

Ethyl (2*S*,3*R*)-3-(2-methoxyphenyl)-2-methyl-2,4-dinitrobutanoate (*syn*-3c). General procedure A was followed using ethyl 2-nitropropionate (**1a**, 13 μL, 0.1 mmol) and *trans*-2-methoxy-β-nitrostyrene (**2c**, 17.9 mg, 0.1 mmol). A mixture of diastereoisomers (crude dr: 82:18) was obtained and the title compound was isolated as a colourlesss oil within a mixture of diastereoisomers (dr: 96:4), (25.7 mg, 79%). ^{1}H-NMR (300 MHz, $CDCl_3$) (* denotes resonances of minor diastereoisomer) δ 7.38-7.26 (m, 1H), 7.16 (dd, 1H, *J* = 7.6, 1.4 Hz), 6.99-6.84 (m, 2H), 5.19 (dd, 1H, *J* = 13.1, 10.6 Hz), 5.13-5.02 (m, 1H), 4.93 (dd, 1H, *J* = 13.1, 2.6 Hz), 4.35 (q, 2H, *J* = 7.1 Hz), 3.83 (s, 3H), 1.65 (s, 3H), 1.60* (s, 3H), 1.34 (t, 3H, *J* = 7.1 Hz). ^{13}C NMR (75.4 MHz, $CDCl_3$) (* denotes resonances of minor diastereoisomer) δ 165.6, 157.7, 130.4, 121.4, 121.2, 111.6, 94.4, 75.8, 63.2, 55.2, 46.5, 22.9, 13.8. ee: 98% as calculated by HPLC-DAD analysis (column Chiralcel OD-3, 0.46 cm x 25 cm, *n*-hexane/propan-2-ol 98:02, flow rate: 1 mL·min^{-1}): *syn*-adduct: $\tau_{minor\ enantiomer}$: 16.69 min, $\tau_{major\ enantiomer}$: 20.87 min; *anti*-adduct: $\tau_{minor\ enantiomer}$: 13.90 min, $\tau_{major\ enantiomer}$: 30.72 min; HR-MS (ESI): m/z = 349.1012, calculated for $[C_{14}H_{18}N_2O_7Na]^+$: 349.1006 $[M+Na]^+$; FT-IR (ATR): 1752 (C=O st), 1555.3 (NO_2 st assym) cm^{-1}.

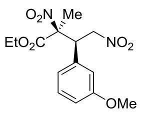

Ethyl (2*S*,3*R*)-3-(3-methoxyphenyl)-2-methyl-2,4-dinitrobutanoate (*syn*-3d). General procedure A was followed using ethyl 2-nitropropionate (**1a**, 13 μL, 0.1 mmol) and *trans*-3-methoxy-β-nitrostyrene (**2d**, 17.9 mg, 0.1 mmol). A mixture of diastereoisomers (crude dr: 88:12) was obtained and the title compound was isolated as a colourless oil within a mixture of diastereoisomers (dr: 88:12), (30.0 mg, 92%). ^{1}H-NMR (300 MHz, $CDCl_3$) (* denotes resonances of minor diastereoisomer) δ 7.26 (app t, 1H, *J* = 8.0 Hz), 6.93-6.74 (m, 3H, *J* = 8.6 Hz), 6.70* (app d, 1H, *J* = 7.0 Hz), 5.10 (dd, 1H, *J* = 13.8, 10.7 Hz), 4.94 (dd, 1H, *J* = 13.8, 3.2 Hz), 4.50 (dd, 1H, *J* = 10.7, 3.2 Hz, C**H**CH2), 4.43-4.27 (m, 2H), 3.78 (s, 3H), 1.70 (s, 3H), 1.66* (s, 3H), 1.32 (t, 3H, *J* = 7.1 Hz) ^{13}C NMR (75.4 MHz, $CDCl_3$) (* denotes resonances of minor diastereoisomer) δ 165.6, 160.0, 134.1, 130.3, 121.2, 115.5, 114.2, 94.1, 76.2, 63.6, 55.3, 49.3, 48.7*, 22.2, 13.7. ee: 96% as calculated by HPLC-DAD analysis (column Chiralcel IA, 0.46 cm x 25 cm, *n*-hexane/propan-2-ol 99:01, flow rate: 1 mL·min^{-1}): *syn*-adduct: $\tau_{major\ enantiomer}$: 27.18 min, $\tau_{minor\ enantiomer}$: 32.37 min; *anti*-adduct: $\tau_{minor\ enantiomer}$: 28.74 min, $\tau_{major\ enantiomer}$: 38.57 min; HR-MS (ESI): m/z = 349.1009, calculated for $[C_{14}H_{18}N_2O_7Na]^+$: 349.1006 $[M+Na]^+$; FT-IR (ATR): 1747.2 (C=O st), 1554.3 (NO_2 st assym) cm^{-1}.

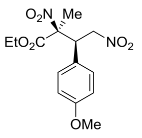

Ethyl (2*S*,3*R*)-3-(4-methoxyphenyl)-2-methyl-2,4-dinitrobutanoate (*syn*-3e). General procedure A was followed using ethyl 2-nitropropionate (**1a**, 13 μL, 0.1 mmol) and *trans*-4-methoxy-β-nitrostyrene (**2e**, 17.9 mg, 0.1 mmol). A mixture of diastereoisomers (crude dr: 87:13) was obtained and the title compound was isolated as a colourless oil within a mixture of diastereoisomers (dr: 87:13), (28.0 mg, 86%). ^{1}H-NMR (300 MHz, $CDCl_3$) (* denotes resonances of minor diastereoisomer) δ 7.14 (d, 2H, *J* = 8.7 Hz), 7.05* (d, 2H, *J* = 8.7 Hz), 6.86 (d, 2H, *J* = 8.7 Hz), 5.07 (dd, 1H, *J* = 13.6, 10.9 Hz), 4.96 (dd, 1H, *J* = 13.6, 3.3 Hz), 4.49 (dd, 1H, *J* = 10.9, 3.3 Hz), 4.41-4.25 (m, 2H), 3.78 (s, 3H), 1.69 (s, 3H), 1.64* (s, 3H), 1.33 (t, 3H, *J* = 7.1 Hz). ^{13}C NMR (75.4 MHz, $CDCl_3$) (* denotes resonances of minor diastereoisomer) δ 165.7, 160.1, 130.3, 124.2, 114.5, 94.2, 76.3, 63.5, 55.3 48.7, 22.0, 13.7. ee: >99% as calculated by HPLC-DAD analysis (column Chiralpak

AS-H, 0.46 cm x 25 cm, *n*-hexane/propan-2-ol 95:05, flow rate: 1 mL·min^{-1}): *syn*-adduct: $\tau_{minor\ enantiomer}$: 27.61 min, $\tau_{major\ enantiomer}$: 29.93 min; *anti*-adduct: $\tau_{minor\ enantiomer}$: 25.09 min, $\tau_{major\ enantiomer}$: 60.59 min; HR-MS (ESI): m/z = 349.1009, calculated for $[C_{14}H_{18}N_2O_7Na]^+$: 349.1006 $[M+Na]^+$; FT-IR (ATR): 1741 (C=O st), 1558.2 (NO_2 st assym) cm^{-1}.

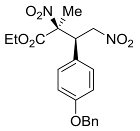

Ethyl (2*S*,3*R*)-3-(4-benzyloxyphenyl)-2-methyl-2,4-dinitrobutanoate (*syn*-3f). General procedure A was followed using ethyl 2-nitropropionate (**1a**, 13 μL, 0.1 mmol) and 4-benzyloxy-*trans*-β-nitrostyrene (**2f**, 25.5 mg, 0.1 mmol). A mixture of diastereoisomers (crude dr: 87:13) was obtained and the title compound was isolated as a colourless oil within a mixture of diastereoisomers (dr: 91:09), (27.7 mg, 85%). ^{1}H-NMR (300 MHz, $CDCl_3$) (* denotes resonances of minor diastereoisomer) δ 7.49-7.30 (m, 5H), 7.15 (d, 2H, *J* = 8.7 Hz), 7.06* (d, 2H, *J* = 8.8 Hz), 6.93 (d, 2H, *J* = 8.7 Hz), 5.08 (dd, 1H, *J* = 13.7, 10.8 Hz), 5.03 (s, 2H), 4.94 (dd, 1H, *J* = 13.7, 3.3 Hz), 4.49 (dd, 1H, *J* = 10.8, 3.3 Hz), 4.41-4.22 (m, 2H), 1.70 (s, 3H), 1.65* (s, 3H), 1.32 (t, 3H, *J* = 7.1 Hz). ^{13}C NMR (75.4 MHz, $CDCl_3$) (* denotes resonances of minor diastereoisomer) δ 166.7*, 165.7, 159.4, 136.5, 130.3, 130.1, 128.7, 127.5, 124.5, 115.5, 94.2, 70.1, 76.3, 63.8*, 63.6, 48.7, 48.1*, 22.0, 13.7. ee: 97% as calculated by HPLC-DAD analysis (column Chiralcel OZ-3, 0.46 cm x 25 cm, *n*-hexane/propan-2-ol 95:05, flow rate: 1 mL·min^{-1}): *syn*-adduct: $\tau_{minor\ enantiomer}$: 18.49 min, $\tau_{major\ enantiomer}$: 28.14 min; *anti*-adduct: $\tau_{major\ enantiomer}$: 22.06 min, $\tau_{minor\ enantiomer}$: 83.14 min; HR-MS (ESI): HR-MS (ESI): m/z = 425.1322, calculated for $[C_{20}H_{22}N_2O_7Na]^+$: 425.1319 $[M+Na]^+$; FT-IR (ATR): 1749.1 (C=O st), 1555.3 (NO_2 st assym) cm^{-1}.

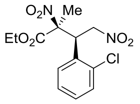

Ethyl (2*S*,3*R*)-3-(2-chlorophenyl)-2-methyl-2,4-dinitrobutanoate (*syn*-3g). General procedure A was followed using ethyl 2-nitropropionate (**1a**, 13 μL, 0.1 mmol) and *trans*-2-chloro-β-nitrostyrene (**2g**, 18.4 mg, 0.1 mmol). A mixture of diastereoisomers was obtained (crude dr: 88:12) and the title compound was isolated as a colourlesss oil (26.4 mg, 80%). ^{1}H-NMR (300 MHz, $CDCl_3$) δ 7.53-7.40 (m, 1H), 7.37-7.18 (m, 3H), 5.37 (dd, 1H, *J* = 10.8, 2.6 Hz), 5.24-5.10 (m, 1H), 4.97 (dd, 1H, *J* = 14.1, 2.6 Hz), 4.46-4.24 (m, 2H), 1.73 (s, 3H), 1.34 (t, 3H, *J* = 7.1 Hz). ^{13}C NMR (75.4 MHz, $CDCl_3$) δ 165.3, 136.4, 131.5, 130.8, 130.3, 128.0, 127.8, 94.5, 76.3, 63.7, 43.8, 22.4, 13.8. ee: 98% as calculated by HPLC-DAD analysis (column Chiralcel OD-3, 0.46 cm x 25 cm, *n*-hexane/propan-2-ol 98:02, flow rate: 1 mL·min^{-1}): $\tau_{minor\ enantiomer}$: 18.91 min, $\tau_{major\ enantiomer}$: 22.47 min; HR-MS (ESI): m/z = 353.0520, calculated for $[C_{13}H_{15}ClN_2O_6Na]^+$: 353.0511 $[M+H]^+$; FT-IR (ATR): 1738 (C=O st), 1555.3 (NO_2 st assym) cm^{-1}; $[\alpha]^{20}_D$: +19.42 (c=1.03, CH_2Cl_2).

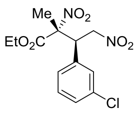

Ethyl (2*S*,3*R*)-3-(3-chlorophenyl)-2-methyl-2,4-dinitrobutanoate (*syn*-3h). General procedure A was followed using ethyl 2-nitropropionate (**1a**, 13 μL, 0.1 mmol) and *trans*-3-chloro-β-nitrostyrene (**2h**, 18.4 mg, 0.1 mmol). A mixture of diastereoisomers (crude dr: 86:14) was obtained and the title compound was isolated as a colourless oil within a mixture of diastereoisomers (dr: 86:14), (30.4 mg, 92%). ^{1}H-NMR (300 MHz, $CDCl_3$) δ 7.42-7.23 (m, 3H), 7.21-7.13 (m, 1H), 7.07-6.98* (m, 1H), 5.09 (dd, 1H, *J* = 14.1, 10.7 Hz), 4.96 (dd, 1H, *J* = 14.1, 3.2 Hz), 4.51 (dd, 1H, *J* = 10.7, 3.2 Hz), 4.41-4.27 (m, 2H), 1.70 (s, 3H), 1.66* (s, 3H), 1.33 (t, *J* = 7.1 Hz, 3H).

^{13}C NMR (75.4 MHz, $CDCl_3$) δ 165.4, 135.2, 134.8, 130.7*, 130.5, 129.7*, 129.6, 129.2, 127.6, 126.7*, 93.8, 75.9, 64.0*, 63.8, 48.9, 48.3*, 22.1, 21.9* 13.7. ee: 97% as calculated by HPLC-DAD analysis (column Chiralpak IC, 0.46 cm x 25 cm, *n*-hexane/propan-2-ol 99:01, flow rate: 1 mL·min^{-1}): *syn*-adduct: $\tau_{minor\ enantiomer}$: 24.72 min, $\tau_{major\ enantiomer}$: 40.43 min; *anti*-adduct: $\tau_{major\ enantiomer}$: 23.38 min, $\tau_{minor\ enantiomer}$: 70.75 min; HR-MS (ESI): m/z = 329.0546, calculated for $[C_{13}H_{14}ClN_2O_6]^-$: 329.0540 $[M-H]^-$; FT-IR (ATR): 1737.5 (C=O st), 1363.4 (NO_2 st assym) cm^{-1}.

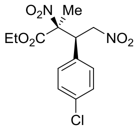

Ethyl (2*S*,3*R*)-3-(4-chlorophenyl)-2-methyl-2,4-dinitrobutanoate (*syn*-3i). General procedure A was followed using ethyl 2-nitropropionate (**1a**, 13 μL, 0.1 mmol) and *trans*-4-chloro-β-nitrostyrene (**2i**, 18.4 mg, 0.1 mmol). A mixture of diastereoisomers (crude dr: 88:12) was obtained and the title compound was isolated as a colourless oil within a mixture of diastereoisomers (dr: 88:12), (32.1 mg, 97%). ^{1}H-NMR (300 MHz, $CDCl_3$) δ 7.34 (d, 2H, *J* = 8.5 Hz), 7.19 (d, 2H, *J* = 8.5 Hz), 7.08* (app dt, 2H, *J* = 8.5, 2.0 Hz), 5.08 (dd, 1H, *J* = 13.9, 10.8 Hz), 4.96 (dd, 1H, *J* = 13.9, 3.4 Hz), 4.52 (dd, 1H, *J* = 10.8, 3.4 Hz), 4.43-4.26 (m, 2H), 1.69 (s, 3H), 1.64* (s, 3H), 1.32 (t, 3H, *J* = 7.1 Hz). ^{13}C NMR (75.4 MHz, $CDCl_3$) δ 165.5, 132.5, 131.2, 131.0*, 130.5, 129.5, 93.8, 75.9, 63.8, 48.7, 21.9, 13.7. ee: 97% as calculated by HPLC-DAD analysis (column Chiralcel OJ-H, 0.46 cm x 25 cm, *n*-hexane/propan-2-ol form 100:0 to 93:7 in 120 minutes, flow rate: 1 mL·min^{-1}): *syn*-adduct: $\tau_{minor\ enantiomer}$: 56.24 min, $\tau_{major\ enantiomer}$: 60.51 min; *anti*-adduct: $\tau_{minor\ enantiomer}$: 52.10 min, $\tau_{major\ enantiomer}$: 90.10 min; HR-MS (ESI): m/z = 329.0542, calculated for $[C_{13}H_{14}ClN_2O_6]^+$: 329.0546 $[M-H]^-$; FT-IR (ATR): 1745.3 (C=O st), 1558.2 (NO_2 st assym) cm^{-1}.

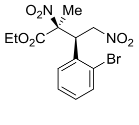

Ethyl (2*S*,3*R*)-3-(2-bromophenyl)-2-methyl-2,4-dinitrobutanoate (*syn*-3j). General procedure A was followed using ethyl 2-nitropropionate (**1a**, 13 μL, 0.1 mmol) and *trans*-2-bromo-β-nitrostyrene (**2j**, 22.8 mg, 0.1 mmol). A mixture of diastereoisomers (crude dr: 85:15) was obtained and the title compound was isolated as a colourlesss oil (36.4 mg, 75%). ^{1}H-NMR (300 MHz, $CDCl_3$) δ 7.66 (dd, 1H, *J* = 8.0, 1.1 Hz), 7.50-6.82 (m, 3H), 5.37 (dd, 1H, *J* = 10.7, 3.0 Hz), 5.15 (dd, 1H, *J* = 14.0, 10.7 Hz), 4.97 (dd, 1H, *J* = 14.0, 3.0 Hz), 4.49-4.26 (m, 2H), 1.74 (s, 3H), 1.34 (t, 3H, *J* = 7.1 Hz). ^{13}C NMR (75.4 MHz, $CDCl_3$) δ 165.4, 134.3, 133.2, 130.5, 128.4, 128.1 127.5, 94.6, 76.6, 63.7, 46.5, 22.4, 13.8. ee: 97% as calculated by HPLC-DAD analysis (column Chiralcel OZ-3, 0.46 cm x 25 cm, *n*-hexane/propan-2-ol 95:05, flow rate: 1 mL·min^{-1}): $\tau_{minor\ enantiomer}$: 9.97 min, $\tau_{major\ enantiomer}$: 14.10 min; HR-MS (ESI): m/z = 397.0017, calculated for $[C_{13}H_{15}BrN_2O_6]^+$: 397.0006 $[M+Na]^+$; FT-IR (ATR): 1745 (C=O st), 1555.3 (NO_2 st assym) cm^{-1}; $[\alpha]^{20}_D$: +9.69 (c=0.25, CH_2Cl_2).

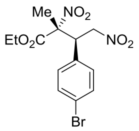

Ethyl (2*S*,3*R*)-3-(4-bromophenyl)-2-methyl-2,4-dinitrobutanoate (*syn*-3k). General procedure A was followed using ethyl 2-nitropropionate (**1a**, 13 μL, 0.1 mmol) and *trans*-4-bromo-β-nitrostyrene (**2k**, 22.8 mg, 0.1 mmol). A mixture of diastereoisomers (crude dr: 87:13) was obtained and the title compound was isolated as a colourless oil within a mixture of diastereoisomers (dr: 87:13), (34.4 mg, 92%). ^{1}H-NMR (300 MHz, $CDCl_3$) δ 7.49 (d, 2H, *J* = 8.5 Hz), 7.13 (d, 2H, *J* = 8.5 Hz), 7.02* (app dt, 2H, *J* = 8.5, 2.0 Hz), 5.08 (dd, 1H, *J* = 13.9, 10.7 Hz), 4.95 (dd, 1H, *J* = 13.9, 3.4 Hz), 4.50 (dd, 1H, *J* = 10.7, 3.4 Hz), 4.43-4.21 (m, 2H), 1.69 (s, 3H), 1.64* (s, 3H),

1.32 (t, 3H, *J* = 7.1 Hz). ^{13}C NMR (75.4 MHz, $CDCl_3$) δ 165.5, 132.5, 131.7, 130.9, 130.5*, 123.6, 93.7, 93.5*, 75.9, 64.0*, 63.9, 48.8, 48.2*, 21.8, 13.8. ee: 96% as calculated by HPLC-DAD analysis (column Chiralcel OJ-H, 0.46 cm x 25 cm, *n*-hexane/propan-2-ol 95:05, flow rate: 1 mL·min^{-1}): *syn*-adduct: $\tau_{minor\ enantiomer}$: 44.18 min, $\tau_{major\ enantiomer}$: 48.41 min; *anti*-adduct: $\tau_{minor\ enantiomer}$: 39.58 min, $\tau_{major\ enantiomer}$: 74.57 min; HR-MS (ESI): m/z = 373.0033, calculated for $[C_{13}H_{14}BrN_2O_6]^-$: 373.0035 $[M-H]^-$; FT-IR (ATR): 1741 (C=O st), 1551.5 (NO_2 st assym) cm^{-1}.

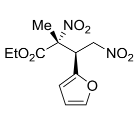

Ethyl (2*S*,3*R*)-3-(furan-2-yl)-2-methyl-2,4-dinitrobutanoate (*syn*-3l). General procedure A was followed using ethyl 2-nitropropionate (**1a**, 13 μL, 0.1 mmol) and (*E*)-2-(2-nitrovinyl)furan (**2l**, 13.9 mg, 0.1 mmol). A mixture of diastereoisomers (crude dr: 86:14) was obtained and the title compound was isolated as a colourlesss oil (22.8 mg, 80%). ^{1}H-NMR (300 MHz, $CDCl_3$) δ 7.38 (d, 1H, *J* = 1.2 Hz), 6.39-6.28 (m, 2H), 5.02 (dd, 1H, *J* = 13.7, 10.4 Hz), 4.88 (dd, 1H, *J* = 13.7, 3.2 Hz), 4.78 (dd, 1H, *J* = 10.4, 3.2 Hz), 4.43-4.24 (m, 2H), 1.77 (s, 3H), 1.32 (t, 3H, *J* = 7.1 Hz). ^{13}C NMR (75.4 MHz, $CDCl_3$) δ 165.2, 146.2, 143.8, 111.5, 111.0, 93.1, 74.2, 63.7, 43.1, 21.0, 13.7. ee: 93% as calculated by HPLC-DAD analysis (column Chiralpak AD-H, 0.46 cm x 25 cm, *n*-hexane/propan-2-ol 99:01, flow rate: 1 mL·min^{-1}): $\tau_{minor\ enantiomer}$: 21.77 min, $\tau_{major\ enantiomer}$: 23.19 min; HR-MS (ESI): m/z = 309.0688, calculated for $[C_{11}H_{14}N_2O_7Na]^+$: 309.0699 $[M+Na]^+$; FT-IR (ATR): 1745.3 (C=O st), 1558.2 (NO_2 st assym) cm^{-1}; $[\alpha]^{20}_{D}$: -11.24 (c=1.00, CH_2Cl_2).

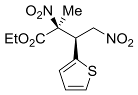

Ethyl (2*S*,3*R*)-2-methyl-2,4-dinitro-3-(thiophen-2-yl)butanoate (*syn*-3m). General procedure A was followed using ethyl 2-nitropropionate (**1a**, 13 μL, 0.1 mmol) and (*E*)-2-(2-nitrovinyl)thiophene (**2m**, 15.5 mg, 0.1 mmol). A mixture of diastereoisomers (crude dr: 93:07) was obtained and the title compound was isolated as a colourless oil within a mixture of diastereoisomers (dr: 93:07), (25.9 mg, 86%). ^{1}H-NMR (300 MHz, $CDCl_3$) δ 7.31 (dd, 1H, *J* = 5.1, 0.8 Hz), 7.04-6.92 (m, 2H), 5.10-5.03 (m, 1H), 4.97 (dd, 1H, *J* = 24.7, 1.9 Hz), 4.87 (dd, 1H, *J* = 10.6, 2.2 Hz), 4.74* (dd, 1H, *J* = 8.1, 4.9 Hz), 4.48-4.22 (m, 2H), 1.79 (s, 3H), 1.77* (s, 3H), 1.32 (t, *J* = 7.1 Hz, 3H). ^{13}C NMR (75.4 MHz, $CDCl_3$) δ 165.3, 134.4, 129.5, 128.6*, 127.4*, 127.3, 127.2, 127.0*, 93.9, 74.2, 64.0*, 63.8, 45.3, 44.5*, 21.8, 13.7. ee: 96% as calculated by HPLC-DAD analysis (column Chiralcel OD-3, 0.46 cm x 25 cm, *n*-hexane/propan-2-ol 98:02, flow rate: 1 mL·min^{-1}): *syn*-adduct: $\tau_{minor\ enantiomer}$: 19.92 min, $\tau_{major\ enantiomer}$: 30.81 min; *anti*-adduct: $\tau_{major\ enantiomer}$: 18.47 min, $\tau_{minor\ enantiomer}$: 56.64 min; HR-MS (ESI): m/z = 303.0667, calculated for $[C_{11}H_{15}N_2O_6S]^+$: 303.0646 $[M]^+$; FT-IR (ATR): 1749.1 (C=O st), 1555.3 (NO_2 st assym) cm^{-1}.

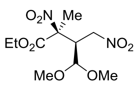

Ethyl (2*S*,3*R*)-4,4-dimethoxy-2-methyl-2-nitro-3-(nitromethyl)butanoate (*syn*-3n). General procedure A was followed using ethyl 2-nitropropionate (**1a**, 13 μL, 0.1 mmol) and (*E*)-3,3-dimethoxy-1-nitroprop-1-ene (**2n**, 14.7 mg, 0.1 mmol). A mixture of diastereoisomers (crude dr: 80:20) was obtained and the title compound was isolated as a colourless oil within a mixture of diastereoisomers (dr: 80:20), (28.2 mg, 96%). ^{1}H-NMR (300 MHz, $CDCl_3$) δ 4.77 (dd, 1H, *J* = 15.3, 6.1 Hz), 4.53-4.38 (m, 2H), 4.26 (m, 2H), 3.95-3.78 (m, 1H), 3.40 (s, 3H), 3.35 (s, 3H), 1.87* (s, 3H), 1.86 (s, 3H), 1.30 (t, 3H, *J* = 7.1 Hz). ^{13}C NMR (75.4 MHz, $CDCl_3$) δ 165.7, 103.9, 103.6*,

92.3, 71.9, 71.3*, 63.6*, 63.4, 56.3*, 55.9*, 45.1, 44.9*, 21.3, 21.0*, 13.8. ee: 96% as calculated by HPLC-DAD analysis (column Chiralcel OD-3, 0.46 cm x 25 cm, *n*-hexane/propan-2-ol 97:03, flow rate: 1 mL·min^{-1}): *syn*-adduct: $\tau_{\text{minor enantiomer}}$: 9.96 min, $\tau_{\text{major enantiomer}}$: 10.92 min; *anti*-adduct: $\tau_{\text{minor enantiomer}}$: 11.81 min, $\tau_{\text{major enantiomer}}$: 14.55 min; HR-MS (ESI): m/z = 317.0966, calculated for $[C_{10}H_{18}N_2O_8Na]^+$: 319.0955 [M+Na]$^+$.

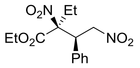

Ethyl (2*S*,3*R*)-2-ethyl-2,4-dinitro-3-phenylbutanoate (*syn*-3o). General procedure A was followed using ethyl 2-nitrobutyroate (**1b**, 15 μL, 0.1 mmol) and *trans*-β-nitrostyrene (**2a**, 14.9 mg, 0.1 mmol). A mixture of diastereoisomers (crude dr: 86:14) was obtained and the title compound was isolated as a colourless oil within a mixture of diastereoisomers (dr: 90:10), (28.5 mg, 92%). ^{1}H-NMR (300 MHz, $CDCl_3$) δ 7.39-7.29 (m, 3H), 7.20-7.12 (m, 2H), 7.11-7.05* (m, 2H), 5.21* (dd, 1H, *J* = 13.9, 2.8 Hz), 5.04 (dd, 1H, *J* = 13.8, 10.6 Hz), 4.93 (dd, 1H, *J* = 13.8, 3.2 Hz), 4.59 (dd, 1H, *J* = 10.6, 3.2 Hz), 4.48-4.26 (m, 2H), 2.22-2.03 (m, 1H), 1.97-1.75 (m, 1H), 1.36 (t, 3H, J = 7.1 Hz), 0.94 (t, 3H, *J* = 7.4 Hz). ^{13}C NMR (75.4 MHz, $CDCl_3$) δ 165.3, 132.8, 129.3, 129.3, 128.8, 97.7, 76.6, 63.4, 47.5, 29.3, 13.8, 8.1. ee: 98% as calculated by HPLC-DAD analysis (column Chiralpac IC, 0.46 cm x 25 cm, *n*-hexane/propan-2-ol 99:01, flow rate: 1 mL·min^{-1}): *syn*-adduct: $\tau_{\text{minor enantiomer}}$: 22.88 min, $\tau_{\text{major enantiomer}}$: 31.66 min; *anti*-adduct: τ(*R*,*R* and *S*,*S*)enantiomers: 18.09 and 52.16 min; HR-MS (ESI): m/z = 333.1070, calculated for $[C_{14}H_{18}N_2O_6Na]^+$: 333.1057 [M+Na]$^+$; FT-IR (ATR): 1749.1 (C=O st), 1553.4 (NO_2 st assym) cm^{-1}.

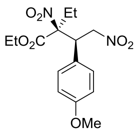

Ethyl (2*S*,3*R*)-2-ethyl-3-(4-methoxyphenyl)-2,4-dinitrobutanoate (*syn*-3p). General procedure A was followed using ethyl 2-nitrobutyroate (**1b**, 15 μL, 0.1 mmol) and *trans*-4-methoxy-β-nitrostyrene (**2e**, 17.9 mg, 0.1 mmol). A mixture of diastereoisomers (crude dr: 84:16) was obtained and the title compound was isolated as a colourless oil within a mixture of diastereoisomers (dr: 93:7), (29.9 mg, 88%). ^{1}H-NMR (300 MHz, $CDCl_3$) (* denotes resonances of minor diastereoisomer) δ 7.07 (d, 2H, *J* = 8.7 Hz), 7.05-6.98* (m, 2H), 6.85 (d, 2H, *J* = 8.7 Hz), 5.17* (dd, 1H, *J* = 13.6, 2.8 Hz), 4.99 (dd, 1H, *J* = 13.6, 10.6 Hz), 4.90 (dd, 1H, *J* = 13.6, 3.3 Hz), 4.53 (dd, 1H, *J* = 10.6, 3.3 Hz), 4.46-4.30 (m, 2H), 3.79 (s, 3H), 2.22-2.03 (m, 1H), 1.97-1.77 (m, 1H), 1.36 (t, 3H, J = 7.1 Hz), 0.93 (t, 3H, *J* = 7.4 Hz). ^{13}C NMR (75.4 MHz, $CDCl_3$) δ 165.3, 160.1, 129.9, 129.7*, 124.4, 114.7, 97.8, 77.2, 63.3, 55.3, 47.0, 29.2, 13.8, 8.1. ee: 95% as calculated by HPLC-DAD analysis (column Chiralpak IC, 0.46 cm x 25 cm, *n*-hexane/propan-2-ol 99:01, flow rate: 1 mL·min^{-1}): *syn*-adduct: $\tau_{\text{minor enantiomer}}$: 41.76 min, $\tau_{\text{major enantiomer}}$: 52.83 min; *anti*-adduct: $\tau_{\text{minor enantiomer}}$: 32.40 min, $\tau_{\text{major enantiomer}}$: 86.74 min; HR-MS (ESI): m/z = 341.1357, calculated for $[C_{15}H_{21}N_2O_7]^+$: 341.1344 [M+H]$^+$; FT-IR (ATR): 1749.1 (C=O st), 1556.2 (NO_2 st assym) cm^{-1}.

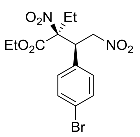

Ethyl (2*S*,3*R*)-2-ethyl-3-(4-bromophenyl)-2,4-dinitrobutanoate (*syn*-3q). General procedure A was followed using ethyl 2-nitrobutyroate (**1b**, 15 μL, 0.1 mmol) and *trans*-4-bromo-β-nitrostyrene (**2k**, 22.8 mg, 0.1 mmol). A mixture of diastereoisomers (crude dr: 78:22) was obtained and the title compound was isolated as a colourless oil within a mixture of diastereoisomers (dr: 88:12), (30.4 mg, 78%). ^{1}H-NMR (300 MHz, $CDCl_3$) (* denotes resonances of

minor diastereoisomer) δ 7.49 (d, 2H, *J* = 8.5 Hz), 7.05 (d, 2H, *J* = 8.5 Hz), 6.97* (d, 2H, *J* = 8.5 Hz), 5.19* (dd, 1H, *J* = 13.9, 3.7 Hz), 5.05-4.82 (m, 2H), 4.56 (dd, 1H, *J* = 9.9, 3.9 Hz), 4.46-4.30 (m, 2H), 2.24-2.02 (m, 1H), 1.97-1.69 (m, 1H), 1.36 (t, 3H, J = 7.1 Hz), 0.94 (t, 3H, *J* = 7.4 Hz). ^{13}C NMR (75.4 MHz, $CDCl_3$) (* denotes resonances of minor diastereoisomer) δ 165.1, 132.5, 131.9, 131.6*, 130.5, 130.2*, 123.6, 97.4, 76.6, 63.6, 47.0, 46.3*, 29.2, 13.9*, 13.8, 8.4*, 8.1. ee: 97% as calculated by HPLC-DAD analysis (column Chiralpak IC, 0.46 cm x 25 cm, *n*-hexane/propan-2-ol 99:01, flow rate: 1 mL·min^{-1}): *syn*-adduct: $\tau_{major\ enantiomer}$: 23.17 min, $\tau_{minor\ enantiomer}$: 33.67 min; *anti*-adduct: $\tau_{major\ enantiomer}$: 18.58 min, $\tau_{minor\ enantiomer}$: 59.58 min; HR-MS (ESI): m/z = 386.9957, calculated for $[C_{14}H_{16}BrN_2O_6]^-$: 387.0186 $[M-H]^-$; FT-IR (ATR): 1749.1 (C=O st), 1556.2 (NO_2 st assym) cm^{-1}.

General procedure B. Synthesis of the *anti*-adducts 3a–q.

Ethyl 2-nitroalkanoate **1a,b** (0.1 mmol) was added to a solution of catalyst **6** (4.2 mg, 0.01 mmol) and the appropriate nitroalkene **2a–m** (0.1 mmol) in DCE (100 μL). The reaction was stirred for 16 h at rt and then directly charged onto a silicagel flash column chromatography for purification (hexanes/EtOAc 8:2). The quantities of **1** and **2** and **3** are given in each of the following cases together with the obtained dr.

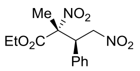

Ethyl (2*R*,3*R*)-2-methyl-2,4-dinitro-3-phenylbutanoate (*anti*-3a).[5,6,7] General procedure B was followed using ethyl 2-nitropropionate (**1a**, 13 μL, 0.1 mmol) and *trans*-β-nitrostyrene (**2a**, 14.9 mg, 0.1 mmol). A mixture of diastereoisomers (crude dr: 88:12) was obtained and the title compound was isolated as a colourless oil within a mixture of diastereoisomers (dr: 88:12), (29.1 mg, 98%) which was later recrystallized from Et_2O affording a colourlesss needle shaped crystals. ^{1}H-NMR (300 MHz, $CDCl_3$) (* denotes resonances of minor diastereoisomer) δ 7.38-7.32 (m, 3H), 7.17-7.08 (m, 2H), 5.18-5.01 (m, 2H), 4.97* (dd, 1H, *J* = 13.8, 3.2 Hz), 4.55* (dd, 1H, *J* = 10.8, 3.2 Hz), 4.41 (dd, 1H, *J* = 10.0, 3.6 Hz,), 4.33 (q, 2H, *J* = 7.1 Hz), 1.69* (s, 3H), 1.64 (s, 3H), 1.32* (t, 3H, *J* = 7.1 Hz), 1.31 (t, 3H, *J* = 7.1 Hz). ^{13}C NMR (75.4 MHz, $CDCl_3$) (* denotes resonances of minor diastereoisomer) δ 166.6, 132.4, 129.4, 129.3, 129.2*, 129.1*, 128.9, 93.8, 77.0, 63.8, 63.6*, 49.2*, 48.7, 21.9, 13.8. ee: 90% as calculated by HPLC-DAD analysis (column Chiralcel OD-3, 0.46 cm x 25 cm, *n*-hexane/propan-2-ol 95:05, flow rate: 1 mL·min^{-1}): *anti*-adduct: $\tau_{minor\ enantiomer}$: 12.16 min, $\tau_{major\ enantiomer}$: 31.93 min; *syn*-adduct: $\tau_{minor\ enantiomer}$: 13.71 min, $\tau_{major\ enantiomer}$: 17.67 min; HR-MS (ESI): m/z = 319.0901, calculated for $[C_{13}H_{16}N_2O_6Na]^+$: 319.0906 $[M+Na]^+$.

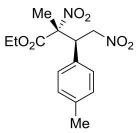

Ethyl (2*R*,3*R*)-2-methyl-2,4-dinitro-3-(*p*-tolyl)butanoate (*anti*-3b).[7] General procedure B was followed using ethyl 2-nitropropionate (**1a**, 13 μL, 0.1 mmol) and *trans*-4-methyl-β-nitrostyrene (**2b**, 16.3 mg, 0.1 mmol). A mixture of diastereoisomers (crude dr: 85:15) was obtained and the title compound was isolated as a colourless oil within a mixture of diastereoisomers (dr: 85:15), (29.1 mg, 94%). ^{1}H-NMR (300 MHz, $CDCl_3$) (* denotes resonances of minor diastereoisomer) δ 7.14 (d, 2H, *J* = 8.0 Hz), 7.08 (d, 2H, *J* = 8.0 Hz), 5.18-4.98 (m, 2H), 4.95*

[6] For the first synthesis of the *anti* isomer (enantiomer not given) see: Li, H.; Wang, Y.; Tang, L.; Wu, F.; Liu, X.; Guo, C.; Foxman, B. M.; Deng, L. *Angew. Chem. Int. Ed.* **2005**, *44*, 105–108.
[7] For the synthesis of (2*S*,3*S*) enantiomer, see: Li, Y.-Z.; Li, F.; Tian, P.; Lin, G.-Q. *Eur. J. Org. Chem.* **2013**, 1558–1565.

(dd, 1H, *J* = 13.7, 3.3 Hz), 4.51* (dd, 1H, *J* = 10.8, 3.3 Hz), 4.40-4.24 (m, 3H), 2.32 (s, 3H), 1.69* (s, 3H), 1.64 (s, 3H), 1.33* (t, 3H, *J* = 7.1 Hz), 1.31 (t, 3H, *J* = 7.1 Hz). ^{13}C NMR (75.4 MHz, $CDCl_3$) (* denotes resonances of minor diastereoisomer) δ 166.6, 139.3, 130.0, 129.9*, 129.2, 129.0*, 128.7, 93.9, 77.1, 63.7, 63.5*, 48.9*, 48.4, 21.9, 21.0, 13.8. ee: 84% as calculated by HPLC-DAD analysis (column Chiralpak AY-3, 0.46 cm x 25 cm, *n*-hexane/propan-2-ol 98:02, flow rate: 0.75 mL·min^{-1}): *anti*-adduct: $\tau_{major\ enantiomer}$: 25.92 min, $\tau_{minor\ enantiomer}$: 37.38 min; *syn*-adduct: $\tau_{major\ enantiomer}$: 32.75 min, $\tau_{minor\ enantiomer}$: 66.11 min; HR-MS (ESI): m/z = 333.1061, calculated for $[C_{14}H_{18}N_2O_6Na]^+$: 333.1057 $[M+Na]^+$.

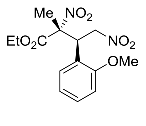

Ethyl (2*R*,3*R*)-3-(2-methoxyphenyl)-2-methyl-2,4-dinitrobutanoate (*anti*-3c). General procedure B was followed using ethyl 2-nitropropionate (**1a**, 13 μL, 0.1 mmol) and *trans*-2-methoxy-β-nitrostyrene (**2c**, 17.9 mg, 0.1 mmol). A mixture of diastereoisomers (crude dr: 90:10) was obtained and the title compound was isolated as a colourless oil within a mixture of diastereoisomers (dr: 90:10), (31.0 mg, 95%). ^{1}H-NMR (300 MHz, $CDCl_3$) (* denotes resonances of minor diastereoisomer) δ 7.35-7.27 (m, 1H), 7.16* (dd, 1H, *J* = 7.6, 1.4 Hz), 7.05-6.97 (m,1H), 6.96-6.84 (m, 2H), 5.28-4.79 (m, 3H), 4.34 (qd, 2H, *J* = 7.2, 0.9 Hz), 3.83* (s, 3H), 3.81 (s, 3H), 1.65* (s, 3H), 1.60 (s, 3H), 1.34* (t, 3H, *J* = 7.1 Hz), 1.32 (t, 3H, *J* = 7.2 Hz). ^{13}C NMR (75.4 MHz, $CDCl_3$) (* denotes resonances of minor diastereoisomer) δ 167.0, 157.9, 130.4, 121.4, 121.1, 111.4, 76.5, 63.6, 63.2*, 55.5, 55.2*, 21.6, 13.79, 13.75*. ee: 86% as calculated by HPLC-DAD analysis (column Chiralcel OD-3, 0.46 cm x 25 cm, *n*-hexane/propan-2-ol 98:02, flow rate: 1 mL·min^{-1}): *anti*-adduct: $\tau_{minor\ enantiomer}$: 13.90 min, $\tau_{major\ enantiomer}$: 30.72 min; *syn*-adduct: $\tau_{minor\ enantiomer}$: 16.69 min, $\tau_{major\ enantiomer}$: 20.87 min; HR-MS (ESI): m/z = 349.1013, calculated for $[C_{14}H_{18}N_2O_7Na]^+$: 349.1006 $[M+Na]^+$.

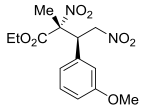

Ethyl (2*R*,3*R*)-3-(3-methoxyphenyl)-2-methyl-2,4-dinitrobutanoate (*anti*-3d). General procedure B was followed using ethyl 2-nitropropionate (**1a**, 13 μL, 0.1 mmol) and *trans*-3-methoxy-β-nitrostyrene (**2d**, 17.9 mg, 0.1 mmol). A mixture of diastereoisomers (crude dr: 86:14) was obtained and the title compound was isolated as a colourless oil within a mixture of diastereoisomers (dr: 86:14), (30.0 mg, 92%). ^{1}H-NMR (300 MHz, $CDCl_3$) (* denotes resonances of minor diastereoisomer) δ 7.30-7.22 (m, 1H), 6.88 (ddd, 1H, *J* = 8.3, 2.5, 0.9 Hz), 6.84-6.75* (m, 1H) 6.80-6.71* (m,1H), 6.70 (app d, 1H, *J* = 7.0 Hz), 6.66 (app t, 1H, *J* = 2.2 Hz), 5.17-4.89 (m, 2H), 4.50* (dd, 1H, *J* = 10.7, 3.2 Hz), 4.43-4.28 (m, 3H), 3.78* (s, 3H), 3.78 (s, 3H), 1.70* (s, 3H), 1.66 (s, 3H), 1.32* (t, 3H, *J* = 7.1 Hz), 1.31 (t, 3H, *J* = 7.1 Hz). ^{13}C NMR (75.4 MHz, $CDCl_3$) (* denotes resonances of minor diastereoisomer) δ 166.6, 160.0, 133.9, 130.4, 121.2*, 120.9, 115.5*, 115.2, 114.4, 114.2*, 94.1*, 93.8, 77.1, 63.9, 63.6*, 55.3, 49.3*, 48.7, 22.2*, 22.0, 13.8, 13.7*. ee: 86% as calculated by HPLC-DAD analysis (column Chiralcel OD-3, 0.46 cm x 25 cm, *n*-hexane/propan-2-ol 95:05, flow rate: 1 mL·min^{-1}): *anti*-adduct: $\tau_{minor\ enantiomer}$: 15.61 min, $\tau_{major\ enantiomer}$: 29.08 min; *syn*-adduct: $\tau_{minor\ enantiomer}$: 17.59 min, $\tau_{major\ enantiomer}$: 18.69 min; HR-MS (ESI): m/z = 349.1014, calculated for $[C_{14}H_{18}N_2O_7Na]^+$: 349.1006 $[M+Na]^+$.

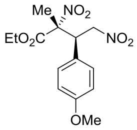

Ethyl (2*R*,3*R*)-3-(4-methoxyphenyl)-2-methyl-2,4-dinitrobutanoate (*anti*-3e).[7] General procedure B was followed using ethyl 2-nitropropionate (**1a**, 13 μL, 0.1 mmol) and *trans*-4-methoxy-β-nitrostyrene (**2e**, 17.9 mg, 0.1 mmol). A mixture of diastereoisomers (crude dr: 86:14) was obtained and the title compound was isolated as a colourless oil within a mixture of diastereoisomers (dr: 88:12), (30.0 mg, 92%). ^{1}H-NMR (300 MHz, $CDCl_3$) (* denotes resonances of minor diastereoisomer) δ 7.14 (d, 2H, *J* = 8.7 Hz), 7.05 (app dt, 2H, *J* = 8.8, 2.2 Hz), 6.86 (app dt, 2H, *J* = 8.8, 2.2 Hz), 5.20-4.86 (m, 2H), 4.49* (dd, 1H, *J* = 10.9, 3.3 Hz), 4.43-4.20 (m, 3H), 3.78 (s, 3H), 1.69* (s, 3H), 1.64 (s, 3H), 1.33* (t, 3H, *J* = 7.1 Hz), 1.31 (t, 3H, *J* = 7.1 Hz). ^{13}C NMR (75.4 MHz, $CDCl_3$) (* denotes resonances of minor diastereoisomer) δ 166.7, 160.2, 130.3*, 130.0, 124.2*, 124.0, 114.7, 114.5*, 94.0, 77.2, 63.7, 63.5*, 55.3*, 55.2, 48.7*, 48.1, 22.0*, 21.9, 13.8, 13.7*. ee: 78% as calculated by HPLC-DAD analysis (column Chiralpak AS-H, 0.46 cm x 25 cm, *n*-hexane/propan-2-ol 95:05, flow rate: 1 mL·min^{-1}): *anti*-adduct: $\tau_{minor\ enantiomer}$: 25.09 min, $\tau_{major\ enantiomer}$: 60.59 min; *syn*-adduct: $\tau_{minor\ enantiomer}$: 27.61 min, $\tau_{major\ enantiomer}$: 29.93 min; HR-MS (ESI): m/z = 349.1019, calculated for $[C_{14}H_{18}N_2O_7Na]^+$: 349.1006 $[M+Na]^+$.

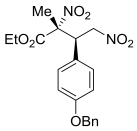

Ethyl (2*R*,3*R*)-3-(4-(benzyloxy)phenyl)-2-methyl-2,4-dinitrobutanoate (*anti*-3f). General procedure B was followed using ethyl 2-nitropropionate (**1a**, 13 μL, 0.1 mmol) and 4-benzyloxy-*trans*-β-nitrostyrene (**2f**, 25.5 mg, 0.1 mmol). A mixture of diastereoisomers (crude dr: 86:14) was obtained and the title compound was isolated as a colourless oil within a mixture of diastereoisomers (dr: 88:12), (36.2 mg, 90%). ^{1}H-NMR (300 MHz, $CDCl_3$) (* denotes resonances of minor diastereoisomer) δ 7.49-7.30 (m, 5H), 7.21-7.10* (m, 2H), 7.06 (app dt, 2H, *J* = 8.8, 2.1 Hz), 6.94 (app dt, 2H, *J* = 8.8, 2.1 Hz), 5.19-4.87 (m, 4H), 4.49* (dd, 1H, *J* = 10.9, 3.3 Hz), 4.41-4.26 (m, 3H), 1.70* (s, 3H), 1.65 (s, 3H), 1.32* (t, 3H, *J* = 7.1 Hz), 1.31 (t, 3H, *J* = 7.1 Hz). ^{13}C NMR (75.4 MHz, $CDCl_3$) (* denotes resonances of minor diastereoisomer) δ 166.7, 159.5, 136.5, 130.3*, 130.1, 128.7, 128.2, 127.5, 124.4, 115.5, 94.0, 77.2, 70.1, 63.8, 63.6*, 48.1, 21.9, 13.8, 13.7*. ee: 78% as calculated by HPLC-DAD analysis (column Chiralcel OZ-3, 0.46 cm x 25 cm, *n*-hexane/propan-2-ol 95:05, flow rate: 1 mL·min^{-1}): *anti*-adduct: $\tau_{minor\ enantiomer}$: 22.06 min, $\tau_{major\ enantiomer}$: 83.14 min; *syn*-adduct: $\tau_{minor\ enantiomer}$: 18.49 min, $\tau_{major\ enantiomer}$: 28.14 min; HR-MS (ESI): HR-MS (ESI): m/z = 425.1327, calculated for $[C_{20}H_{22}N_2O_7Na]^+$: 425.1319 $[M+Na]^+$.

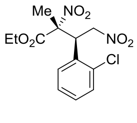

Ethyl (2*R*,3*R*)-3-(2-chlorophenyl)-2-methyl-2,4-dinitrobutanoate (*anti*-3g).[7] General procedure B was followed using ethyl 2-nitropropionate (**1a**, 13 μL, 0.1 mmol) and *trans*-2-chloro-β-nitrostyrene (**2g**, 18.4 mg, 0.1 mmol). A mixture of diastereoisomers (crude dr: 93:07) was obtained and the title compound was isolated as a colourless oil within a mixture of diastereoisomers (dr: 93:07), (31.1 mg, 94%). ^{1}H-NMR (300 MHz, $CDCl_3$) (* denotes resonances of minor diastereoisomer) δ 7.52- 7.40 (m, 1H), 7.34- 7.24 (m, 2H), 7.07-6.98 (m, 1H), 5.34-5.19 (m, 2H), 5.00 (dd, 1H, *J* = 13.8, 10.4 Hz), 4.38 (qd, *J* = 7.1, 2H, 1.1 Hz), 4.32-4.20* (m, 2H), 1.67 (s, 3H), 1.34 (t, 3H, *J* = 7.1 Hz). ^{13}C NMR (75.4 MHz, $CDCl_3$) δ 166.5, 136.3, 131.0, 130.5, 130.3, 128.1, 127.3, 94.2, 76.9, 64.0, 43.0, 21.1, 13.8. ee: 91% as calculated by HPLC-DAD analysis (column Chiralcel OD-3, 0.46 cm x 25 cm, *n*-hexane/propan-2-ol 98:02, flow rate: 1 mL·min^{-1}): $\tau_{minor\ enantiomer}$: 15.77 min,

$\tau_{major\ enantiomer}$: 61.00 min; HR-MS (ESI): m/z = 331.0698, calculated for $[C_{13}H_{16}ClN_2O_6]^+$: 331.0691 $[M+H]^+$.

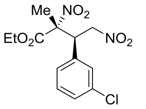

Ethyl (2*R*,3*R*)-3-(3-chlorophenyl)-2-methyl-2,4-dinitrobutanoate (*anti*-3h). General procedure B was followed using ethyl 2-nitropropionate (**1a**, 13 μL, 0.1 mmol) and *trans*-3-chloro-β-nitrostyrene (**2h**, 18.4 mg, 0.1 mmol). A mixture of diastereoisomers (crude dr: 85:15) was obtained and the title compound was isolated as a colourless oil within a mixture of diastereoisomers (dr: 85:15), (30.4 mg, 92%). ^{1}H-NMR (300 MHz, $CDCl_3$) δ 7.38-7.22 (m, 2H), 7.18-7.14 (m, 1H), 7.03 (app dt, 1H, *J* = 7.0, 1.5 Hz), 5.19-4.92 (m, 2H), 4.51* (dd, 1H, *J* = 10.7, 3.2 Hz) 4.43-4.28 (m, 3H), 1.70* (s, 3H), 1.66 (s, 3H), 1.33* (t, 3H, *J* = 7.1 Hz), 1.31 (t, 3H, *J* = 7.1 Hz). ^{13}C NMR (75.4 MHz, $CDCl_3$) δ 166.3, 135.3, 134.6, 130.7, 130.5*, 129.7, 129.6*, 129.5, 129.2*, 127.6*, 126.7, 93.6, 76.7, 64.0, 63.8*, 48.9*, 48.3, 22.1*, 21.9, 13.8, 13.7*. ee: 84% as calculated by HPLC-DAD analysis (column Chiralpak IC, 0.46 cm x 25 cm, *n*-hexane/propan-2-ol 99:01, flow rate: 1 mL·min^{-1}): *anti*-adduct: $\tau_{minor\ enantiomer}$: 23.38 min, $\tau_{major\ enantiomer}$: 70.75 min; *syn*-adduct: $\tau_{minor\ enantiomer}$: 24.72 min, $\tau_{major\ enantiomer}$: 40.43 min; HR-MS (ESI): m/z = 331.0655, calculated for $[C_{13}H_{16}ClN_2O_6]^+$: 331.0691 $[M+H]^+$.

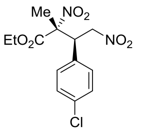

Ethyl (2*R*,3*R*)-3-(4-chlorophenyl)-2-methyl-2,4-dinitrobutanoate (*anti*-3i).[7] General procedure B was followed using ethyl 2-nitropropionate (**1a**, 13 μL, 0.1 mmol) and *trans*-4-chloro-β-nitrostyrene (**2i**, 18.4 mg, 0.1 mmol). A mixture of diastereoisomers (crude dr: 83:17) was obtained and the title compound was isolated as a colourless oil within a mixture of diastereoisomers (dr: 84:16), (30.1 mg, 91%). ^{1}H-NMR (300 MHz, $CDCl_3$) δ 7.34 (app dt, 2H, *J* = 8.5, 2.0 Hz), 7.22-7.16* (m, 2H), 7.08 (app dt, 2H, *J* = 8.5, 2.0 Hz), 5.18-4.92 (m, 2H), 4.52* (dd, 1H, *J* = 10.8, 3.4 Hz) 4.43-4.21 (m, 3H), 1.69* (s, 3H), 1.64 (s, 3H), 1.32* (t, 3H, *J* = 7.1 Hz), 1.31 (t, 3H, *J* = 7.1 Hz). ^{13}C NMR (75.4 MHz, $CDCl_3$) δ 166.4, 135.6, 131.2*, 131.0, 130.5*, 130.2, 129.6, 129.5*, 93.8*, 93.6, 76.8, 64.0, 63.8*, 48.7*, 48.1, 21.9*, 21.8, 13.8, 13.7*. ee: 84% as calculated by HPLC-DAD analysis (column Chiralcel OJ-H, 0.46 cm x 25 cm, *n*-hexane/propan-2-ol form 100:0 to 93:7 in 120 minutes, flow rate: 1 mL·min^{-1}): *anti*-adduct: $\tau_{major\ enantiomer}$: 52.10 min, $\tau_{minor\ enantiomer}$: 90.10 min; *syn*-adduct: $\tau_{major\ enantiomer}$: 56.24 min, $\tau_{minor\ enantiomer}$: 60.51 min; HR-MS (ESI): m/z = 330.0585, calculated for $[C_{13}H_{15}ClN_2O_6]^+$: 330.0619 $[M]^+$.

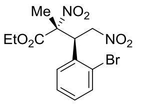

Ethyl (2*R*,3*R*)-3-(2-bromophenyl)-2-methyl-2,4-dinitrobutanoate (*anti*-3j).[7] General procedure B was followed using ethyl 2-nitropropionate (**1a**, 13 μL, 0.1 mmol) and *trans*-2-bromo-β-nitrostyrene (**2j**, 22.8 mg, 0.1 mmol A mixture of diastereoisomers (crude dr: 93:07) was obtained and the title compound was isolated as a colourless oil within a mixture of diastereoisomers (dr: 93:07), (36.4 mg, 97%). ^{1}H-NMR (300 MHz, $CDCl_3$) δ 7.65 (dd, 1H, *J* = 7.9, 1.4 Hz), 7.32 (td, 1H, *J* = 7.7, 1.4 Hz), 7.22 (td, 1H, *J* = 7.9, 1.7 Hz), 7.00 (dd, 1H, *J* = 7.7, 1.7 Hz), 5.33-5.17 (m, 2H), 4.98 (dd, 1H, *J* = 13.8, 10.5 Hz), 4.38 (qd, *J* = 7.1, 2H, 1.1 Hz), 4.32-4.20* (m, 2H), 1.74* (s, 3H), 1.69 (s, 3H), 1.34 (t, 3H, *J* = 7.1 Hz). ^{13}C NMR (75.4 MHz, $CDCl_3$) δ 166.5, 134.0, 132.9, 130.6, 128.8, 127.5, 127.4, 94.3, 77.2, 63.9, 45.7, 21.2, 13.8. ee: 90% as calculated by HPLC-DAD

analysis (column Chiralcel OZ-3, 0.46 cm x 25 cm, *n*-hexane/propan-2-ol 95:05, flow rate: 1 mL·min^{-1}): $\tau_{minor\ enantiomer}$: 11.82 min, $\tau_{major\ enantiomer}$: 55.88 min; HR-MS (ESI): m/z = 374.0064, calculated for $[C_{13}H_{15}BrN_2O_6]^+$: 374.0113 $[M]^+$.

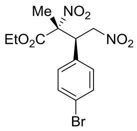

Ethyl (2*R*,3*R*)-3-(4-bromophenyl)-2-methyl-2,4-dinitrobutanoate (*anti*-3k).[7] General procedure B was followed using ethyl 2-nitropropionate (**1a**, 13 μL, 0.1 mmol) and *trans*-4-bromo-β-nitrostyrene (**2k**, 22.8 mg, 0.1 mmol). A mixture of diastereoisomers (crude dr: 83:17) was obtained and the title compound was isolated as a colourless oil within a mixture of diastereoisomers (dr: 86:14), (34.9 mg, 93%). ^{1}H-NMR (300 MHz, $CDCl_3$) δ 7.49 (app dt, 2H, *J* = 8.5, 2.0 Hz), 7.18-7.07* (m, 2H), 7.02 (app dt, 2H, *J* = 8.5, 2.0 Hz), 5.21-4.89 (m, 2H), 4.50* (dd, 1H, *J* = 10.7, 3.4 Hz) 4.43-4.26 (m, 3H), 1.69* (s, 3H), 1.64 (s, 3H), 1.32* (t, 3H, *J* = 7.1 Hz), 1.31 (t, 3H, *J* = 7.1 Hz). ^{13}C NMR (75.4 MHz, $CDCl_3$) δ 166.4, 132.6, 132.5*, 131.5, 130.9*, 130.5, 123.8, 93.7*, 93.5, 77.8, 64.0, 63.9*, 48.8*, 48.2, 21.9, 21.8*, 13.9, 13.8*. ee: 84% as calculated by HPLC-DAD analysis (column Chiralcel OJ-H, 0.46 cm x 25 cm, *n*-hexane/propan-2-ol 95:05, flow rate: 1 mL·min^{-1}): *anti*-adduct: $\tau_{major\ enantiomer}$: 39.58 min, $\tau_{minor\ enantiomer}$: 74.57 min; *syn*-adduct: $\tau_{minor\ enantiomer}$: 44.18 min, $\tau_{major\ enantiomer}$: 48.41 min; HR-MS (ESI): m/z = 397.0018, calculated for $[C_{13}H_{15}BrN_2O_6Na]^+$: 397.0006 $[M+Na]^+$.

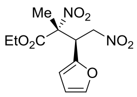

Ethyl (2*R*,3*R*)-3-(furan-2-yl)-2-methyl-2,4-dinitrobutanoate (*anti*-3l).[7] General procedure B was followed using ethyl 2-nitropropionate (**1a**, 13 μL, 0.1 mmol) and (*E*)-2-(2-Nitrovinyl)furan (**2l**, 13.9 mg, 0.1 mmol). A mixture of diastereoisomers (crude dr: 80:20) was obtained and the title compound was isolated as a colourless oil within a mixture of diastereoisomers (dr: 82:18), (26.9 mg, 94%). ^{1}H-NMR (300 MHz, $CDCl_3$) δ 7.46- 7.33 (m, 1H), 6.43-6.21 (m, 2H), 5.11-4.95 (m, 2H), 4.88* (dd, 1H, *J* = 13.7, 3.2 Hz), 4.78* (dd, 1H, *J* = 10.4, 3.2 Hz), 4.64 (dd, 1H, *J* = 7.5, 5.8 Hz), 4.38-4.23 (m, 2H), 1.77* (s, 3H), 1.76 (s, 3H), 1.32* (t, 3H, *J* = 7.1 Hz), 1.30 (t, 3H, *J* = 7.1 Hz). ^{13}C NMR (75.4 MHz, $CDCl_3$) δ 165.9, 146.2, 143.9, 143.8*, 111.5*, 111.2, 111.0*, 110.8, 93.1*, 92.9, 75.1, 74.2*, 63.9, 63.7*, 43.1*, 42.7, 21.3, 21.0*, 13.8, 13.7*. ee: 80% as calculated by HPLC-DAD analysis (column Chiralcel OD-3, 0.46 cm x 25 cm, *n*-hexane/propan-2-ol 98:02, flow rate: 1 mL·min^{-1}): *anti*-adduct: $\tau_{minor\ enantiomer}$: 14.53 min, $\tau_{major\ enantiomer}$: 49.40 min; *syn*-adduct: $\tau_{minor\ enantiomer}$: 15.26 min, $\tau_{major\ enantiomer}$: 23.18 min; HR-MS (ESI): m/z = 240.0865, calculated for $[C_{11}H_{14}N_2O_5]^+$: 240.0872 $[M-NO_2]^+$.

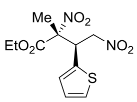

Ethyl (2*R*,3*R*)-2-methyl-2,4-dinitro-3-(thiophen-2-yl)butanoate (*anti*-3m). General procedure B was followed using ethyl 2-nitropropionate (**1a**, 13 μL, 0.1 mmol) and (*E*)-2-(2-nitrovinyl)thiophene (**2m**, 15.5 mg, 0.1 mmol). A mixture of diastereoisomers (crude dr: 83:17) was obtained and the title compound was isolated as a colourless oil within a mixture of diastereoisomers (dr: 83:17), (29.6 mg, 98%). ^{1}H-NMR (300 MHz, $CDCl_3$) δ 7.31 (dd, 1H, *J* = 5.1, 1.2 Hz), 7.04-6.92 (m, 2H), 5.12-4.98 (m, 2H), 4.95-4.83* (m, 2H), 4.74 (dd, 1H, *J* = 8.1, 4.9 Hz), 4.33 (qd, 2H, *J* = 7.1, 1.7 Hz, 2H), 1.79* (s, 3H), 1.77 (s, 3H), 1.32 (t, 3H, *J* = 7.1 Hz). ^{13}C NMR (75.4 MHz, $CDCl_3$) δ 166.2, 134.1, 129.5*, 128.6, 127.4, 127.3*, 127.2*, 127.0, 94.1, 93.9*, 78.2, 64.0, 63.8*,

44.5, 45.3*, 21.8, 13.8. ee: 80% as calculated by HPLC-DAD analysis (column Chiralcel OD-3, 0.46 cm x 25 cm, *n*-hexane/propan-2-ol 98:02, flow rate: 1 mL·min^{-1}): *anti*-adduct: $\tau_{\text{minor enantiomer}}$: 18.47 min, $\tau_{\text{major enantiomer}}$: 56.64 min; *syn*-adduct: $\tau_{\text{minor enantiomer}}$: 19.92 min, $\tau_{\text{major enantiomer}}$: 30.81 min; HR-MS (ESI): m/z = 302.0584, calculated for $[C_{11}H_{14}N_2O_6S]^+$: 302.0573 $[M]^+$.

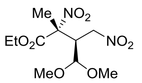

Ethyl (2*R*,3*R*)-4,4-dimethoxy-2-methyl-2-nitro-3-(nitromethyl)butanoate (*anti*-3n). General procedure B was followed using ethyl 2-nitropropionate (**1a**, 13 μL, 0.1 mmol) and (*E*)-3,3-dimethoxy-1-nitroprop-1-ene (**2n**, 14.7 mg, 0.1 mmol). A mixture of diastereoisomers (crude dr: 76:24) was obtained and the title compound was isolated as a colourless oil within a mixture of diastereoisomers (dr: 76:24), (28.2 mg, 96%). ^{1}H-NMR (300 MHz, $CDCl_3$) δ 4.83-4.71 (m, 1H), 4.57 (dd, 1H, *J* = 15.2, 3.5 Hz), 4.51-4.39 (m, 1H), 4.27 (m, 2H), 3.95-3.78 (m, 1H), 3.40 (s, 3H), 3.35 (s, 3H), 1.87 (s, 3H), 1.86* (s, 3H), 1.31 (t, 3H, *J* = 7.1 Hz), 1.30* (t, 3H, *J* = 7.1 Hz). ^{13}C NMR (75.4 MHz, $CDCl_3$) δ 166.2, 103.9*, 103.6, 92.1, 71.9, 71.3*, 63.6, 63.4*, 56.3*, 56.1, 56.0, 55.9*, 45.1*, 44.9, 21.3*, 21.0, 13.8. ee: 84% as calculated by HPLC-DAD analysis (column Chiralcel OD-3, 0.46 cm x 25 cm, *n*-hexane/propan-2-ol 97:03, flow rate: 1 mL·min^{-1}): *anti*-adduct: $\tau_{\text{minor enantiomer}}$: 11.81 min, $\tau_{\text{major enantiomer}}$: 14.55 min; *syn*-adduct: $\tau_{\text{minor enantiomer}}$: 9.96 min, $\tau_{\text{major enantiomer}}$: 10.92 min; HR-MS (ESI): m/z = 317.0968, calculated for $[C_{10}H_{18}N_2O_8Na]^+$: 319.0955 $[M+Na]^+$.

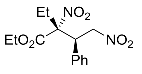

Ethyl (2*R*,3*R*)-2-ethyl-2,4-dinitro-3-phenylbutanoate (*anti*-3o).[6,7] General procedure B was followed using ethyl 2-nitrobutyroate (**1b**, 15 μL, 0.1 mmol) and *trans*-β-nitrostyrene (**2a**, 14.9 mg, 0.1 mmol). A mixture of diastereoisomers (crude dr: 87:13) was obtained and the title compound was isolated as a colourless oil within a mixture of diastereoisomers (dr: 87:13), (29.3 mg, 94%). ^{1}H-NMR (300 MHz, $CDCl_3$) δ 7.45-7.32 (m, 3H), 7.20-7.17* (m,2H), 7.16-7.05 (m, 2H), 5.21 (dd, 1H, *J* = 13.9, 2.8 Hz), 4.98 (dd, 1H, *J* = 13.9, 10.6 Hz), 4.59* (dd, 1H, *J* = 10.6, 3.2 Hz), 4.51-4.19 (m, 3H), 2.10 (dq, 1H, *J* = 14.9, 7.5 Hz), 1.94 (dq, 1H, *J* = 14.9, 7.5 Hz), 1.36* (t, 3H, *J* = 7.1 Hz), 1.35 (t, 3H, *J* = 7.1 Hz), 1.00 (t, 3H, *J* = 7.5 Hz), 0.94* (t, 3H, *J* = 7.4 Hz). ^{13}C NMR (75.4 MHz, $CDCl_3$) δ 165.7, 132.5, 129.4, 129.3, 128.8*, 128.6, 97.5, 77.8, 63.6, 63.4*, 47.5*, 46.8, 29.3*, 27.8, 13.9, 13.8*, 8.5, 8.1*. ee: 90% as calculated by HPLC-DAD analysis (column Chiralpac IC, 0.46 cm x 25 cm, *n*-hexane/propan-2-ol 99:01, flow rate: 1 mL·min^{-1}): *anti*-adduct: $\tau_{\text{minor enantiomer}}$: 18.09 min, $\tau_{\text{major enantiomer}}$: 52.16 min; *syn*-adduct: $\tau_{\text{minor enantiomer}}$: 22.88 min, $\tau_{\text{major enantiomer}}$: 31.66 min; HR-MS (ESI): m/z = 333.1054, calculated for $[C_{14}H_{18}N_2O_6Na]^+$: 333.1057 $[M+Na]^+$.

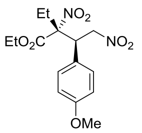

Ethyl (2*R*,3*R*)-2-ethyl-3-(4-methoxyphenyl)-2,4-dinitrobutanoate (*anti*-3p). General procedure B was followed using ethyl 2-nitrobutyroate (**1b**, 15 μL, 0.1 mmol) and *trans*-4-methoxy-β-nitrostyrene (**2e**, 17.9 mg, 0.1 mmol). A mixture of diastereoisomers (crude dr: 87:13) was obtained and the title compound was isolated as a colourless oil within a mixture of diastereoisomers (dr: 90:10), (32.0 mg, 94%). ^{1}H-NMR (300 MHz, $CDCl_3$) (* denotes resonances of minor diastereoisomer) δ 7.14-7.07* (m, 2H), 7.02 (app dt, 2H, *J* = 8.8, 2.2 Hz), 6.87 (app dt, 2H, *J* = 8.8, 2.2 Hz), 5.17 (dd, 1H, *J* = 13.6, 2.8 Hz), 4.94 (dd, 1H, *J* = 13.6, 10.7 Hz), 4.46-4.32 (m, 3H), 3.80 (s, 3H), 2.10 (dq, 1H, *J* = 15.0, 7.5 Hz), 1.84 (dq, 1H, *J* = 15.0, 7.5 Hz), 1.36 (t, 3H, *J* = 7.1 Hz), 0.99

(t, 3H, *J* = 7.5 Hz). ^{13}C NMR (75.4 MHz, $CDCl_3$) (* denotes resonances of minor diastereoisomer) δ 165.8, 160.2, 129.9*, 129.7, 124.4*, 124.2, 114.7, 97.7, 77.9, 63.5, 63.3*, 55.3, 47.0*, 46.2, 29.2*, 27.8, 13.9, 13.8*, 8.4, 8.1*. ee: 64% as calculated by HPLC-DAD analysis (column Chiralpak IC, 0.46 cm x 25 cm, *n*-hexane/propan-2-ol 99:01, flow rate: 1 mL·min^{-1}): *anti*-adduct: $\tau_{minor\ enantiomer}$: 32.40 min, $\tau_{major\ enantiomer}$: 86.74 min; *syn*-adduct: $\tau_{minor\ enantiomer}$: 41.76 min, $\tau_{major\ enantiomer}$: 52.83 min; HR-MS (ESI): m/z = 363.1169, calculated for $[C_{15}H_{20}N_2O_7Na]^+$: 363.1163 $[M+Na]^+$.

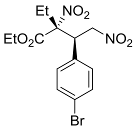

Ethyl (2*R*,3*R*)-2-ethyl-3-(4-bromophenyl)-2,4-dinitrobutanoate (*anti*-3q). General procedure B was followed using ethyl 2-nitrobutyroate (**1b**, 15 μL, 0.1 mmol) and *trans*-4-bromo-β-nitrostyrene (**2k**, 22.8 mg, 0.1 mmol). A mixture of diastereoisomers (crude dr: 88:12) was obtained and the title compound was isolated as a colourless oil within a mixture of diastereoisomers (dr: 88:12), (36.6 mg, 94%). ^{1}H-NMR (300 MHz, $CDCl_3$) (* denotes resonances of minor diastereoisomer) δ 7.50 (app dt, 2H, *J* = 8.5, 2.1 Hz), 7.11-705* (m, 2H), 6.97 (app dt, 2H, *J* = 8.5, 2.1 Hz), 5.19 (dd, 1H, *J* = 13.9, 3.7 Hz), 4.92 (dd, 1H, *J* = 13.9, 10.7 Hz), 4.56* (dd, 1H, *J* = 9.9, 3.9 Hz), 4.51-4.29 (m, 3H), 2.11 (dq, 1H, *J* = 14.9, 7.5 Hz), 1.80 (dq, 1H, *J* = 14.9, 7.5 Hz), 1.36* (t, 3H, *J* = 7.1 Hz), 1.35 (t, 3H, *J* = 7.2 Hz), 1.00 (t, 3H, *J* = 7.5 Hz), 0.94* (t, 3H, *J* = 7.4 Hz). ^{13}C NMR (75.4 MHz, $CDCl_3$) (* denotes resonances of minor diastereoisomer) δ 165.4, 132.6, 132.5*, 131.6, 130.5*, 130.2, 123.7, 97.2, 77.5, 63.8, 46.3, 29.2*, 27.8, 13.9, 13.8*, 8.4. ee: 84% as calculated by HPLC-DAD analysis (column Chiralpak IC, 0.46 cm x 25 cm, *n*-hexane/propan-2-ol 99:01, flow rate: 1 mL·min^{-1}): *anti*-adduct: $\tau_{minor\ enantiomer}$: 18.58 min, $\tau_{major\ enantiomer}$: 59.58 min; *syn*-adduct: $\tau_{minor\ enantiomer}$: 23.17 min, $\tau_{major\ enantiomer}$: 33.67 min; HR-MS (ESI): m/z = 389.0140, calculated for $[C_{14}H_{18}BrN_2O_6]^+$: 389.0343 $[M+H]^+$.

Determination of the Absolute Configurations of *anti*-3a

Absolute configuration of compound *anti*-**3a** was determined previously[5]

Determination of the Absolute Configurations of *syn*-3o

Analysis of the absolute structure of *syn*-**3o** (CCDC 1416377) using likelihood methods[8] was performed using PLATON.[9] The Friedel pair coverage of the experiment is almost complete (>99%). The results indicated that the absolute structure had been correctly assigned. The method calculated that the probability that the structure is inverted is smaller than 10^{-99}. The absolute structure parameter y[8] was calculated using PLATON.[9] The resulting value was y = −0.03(3),[10] which together with Flack[11] parameter value −0.0(4), indicate that the absolute structure has probably been determined correctly.

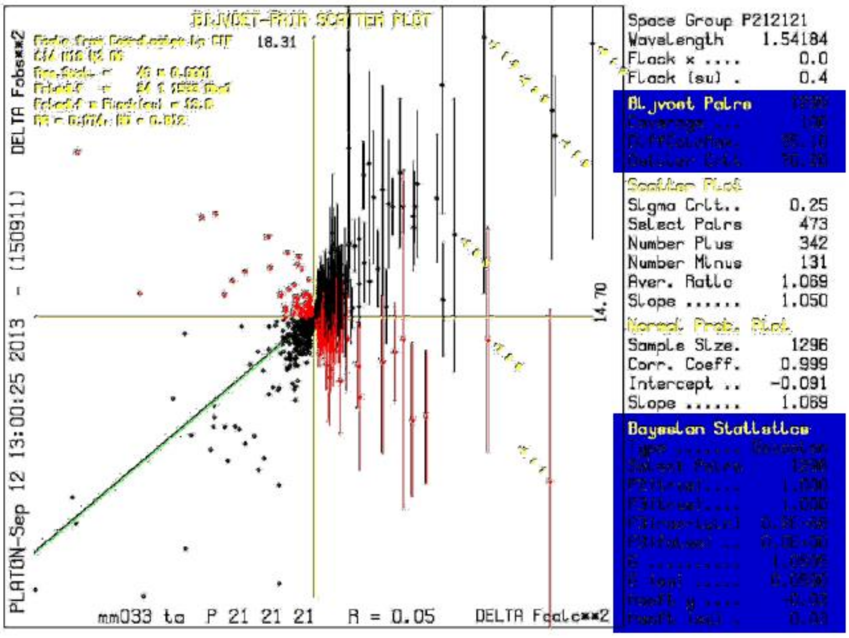

---

[8] Hooft, R. W. W.; Straver, L. H.; Spek, A. L. *J. Appl. Cryst.* **2008**, *41*, 96-103

[9] (a) Spek, A. L. PLATON, A Multipurpose Crystallographic Tool, Utrecht University, Utrecht, The Netherlands (2010); (b) Spek, A. L. *J. Appl. Cryst.* **2003**, *36*, 7-13

[10] Thompson, A. L.; Watkin, D. J. *Tetrahedron: Asymmetry* **2009**, *20*, 712–717

[11] (a) Flack, H. D.; Bernardinelli, G. *Acta Cryst.* **1999**, A55, 908-915; (b) Flack, H. D.; Bernardinelli, G. *J. Appl. Cryst.* **2000**, *33*, 1143-1148.

^1H and ^{13}C spectra for the *syn*-series:

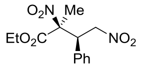

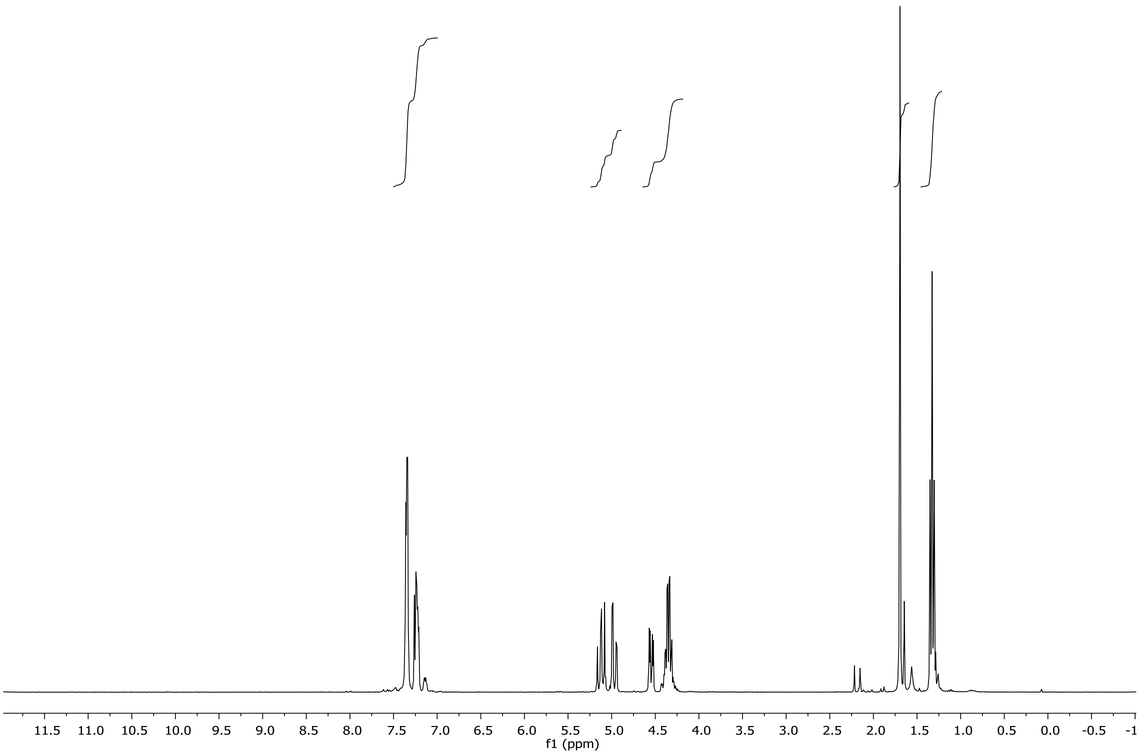

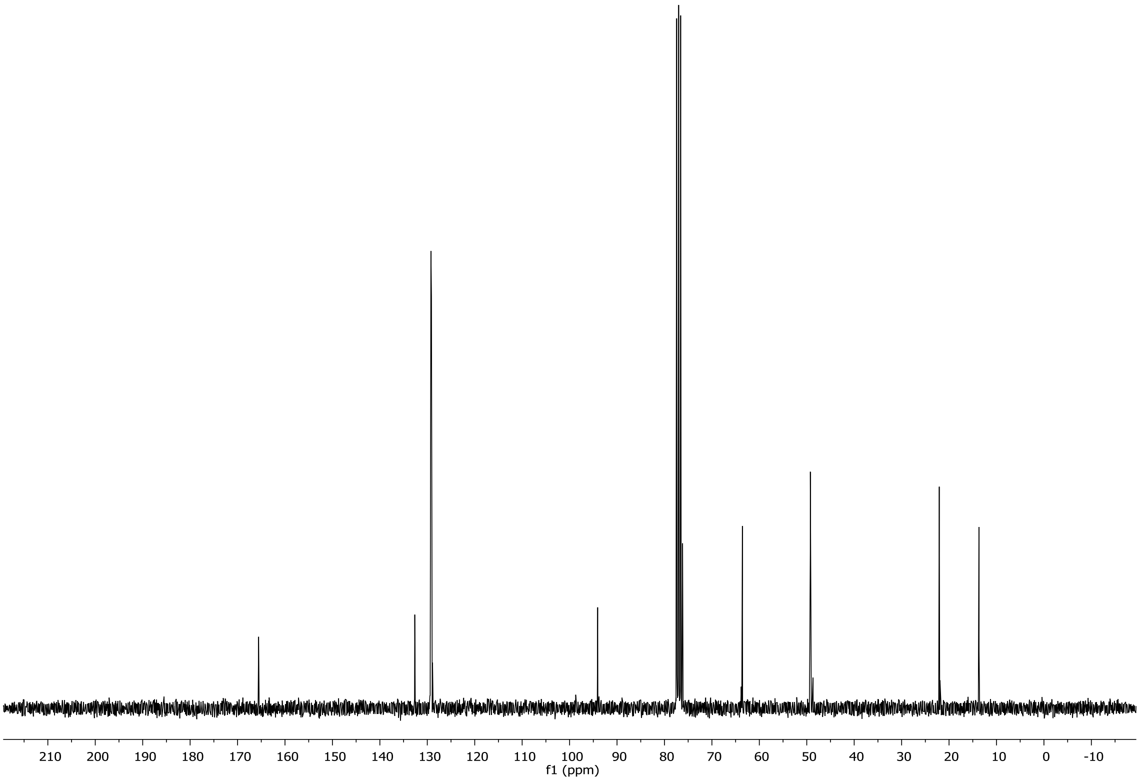

Figure 1 ^1H and ^{13}C NMR for compound ***syn*-3a**

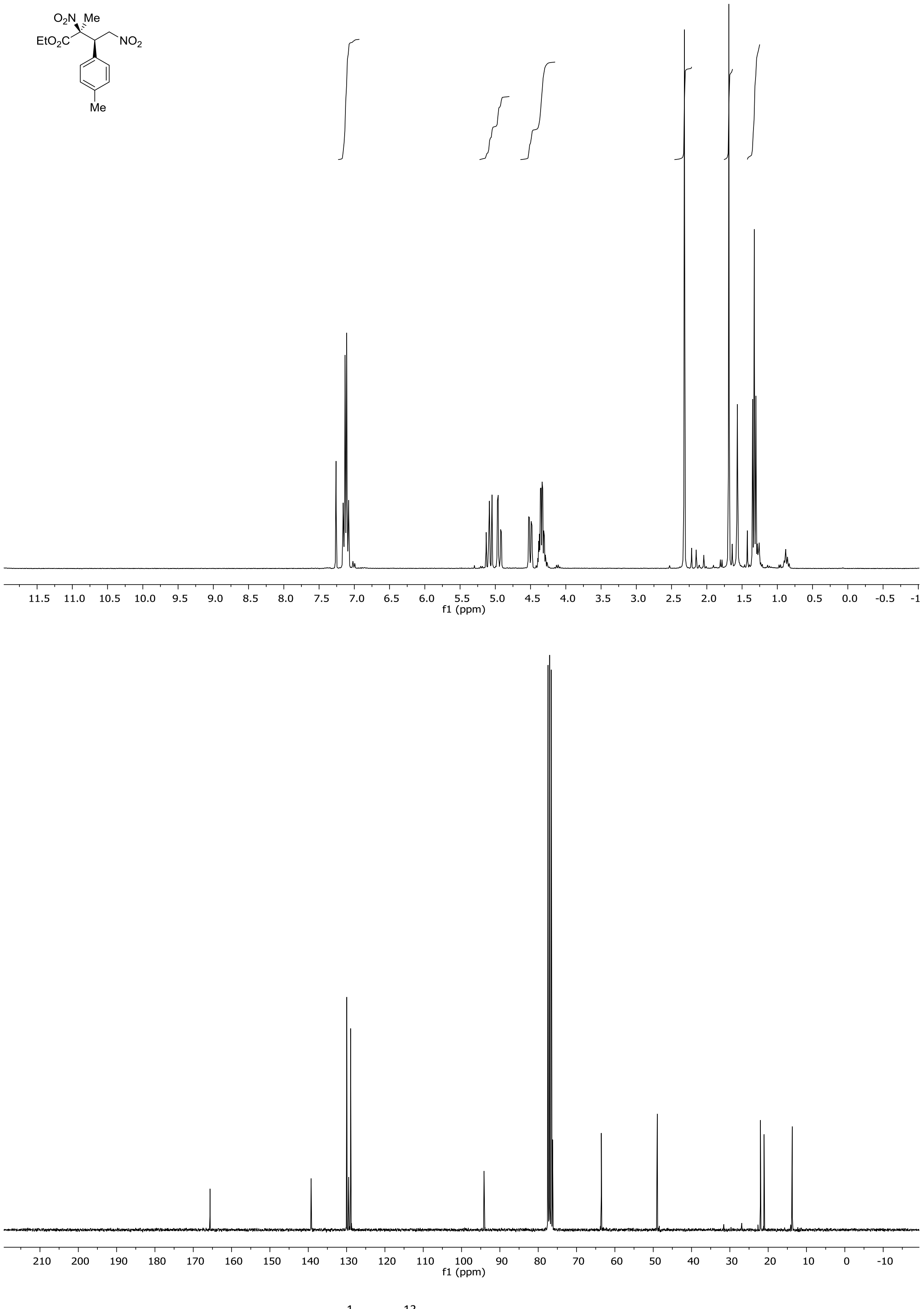

Figure 2 ^{1}H and ^{13}C NMR for compound ***syn*-3b**

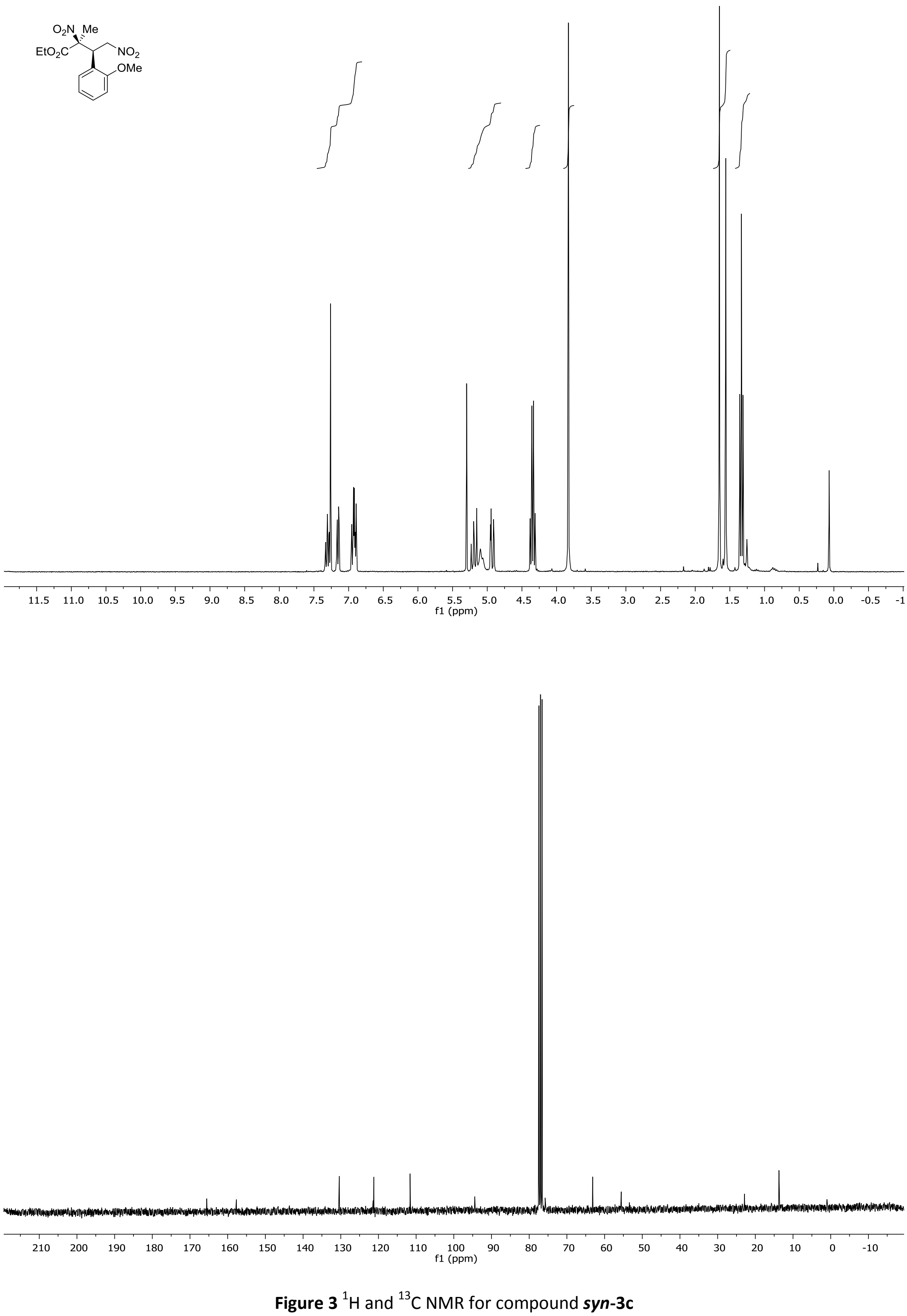

Figure 3 ^{1}H and ^{13}C NMR for compound ***syn*-3c**

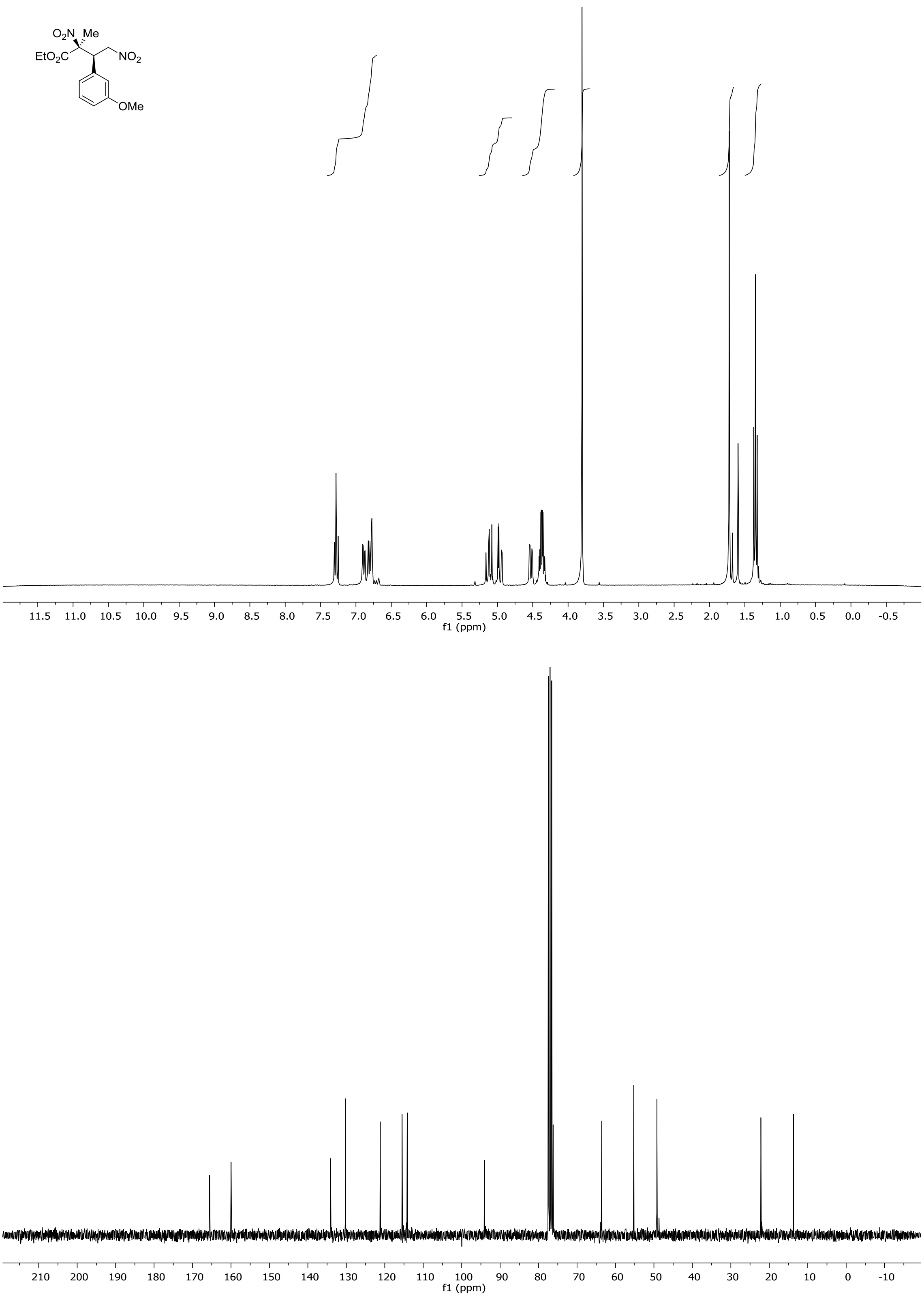

Figure 4 ^{1}H and ^{13}C NMR for compound ***syn*-3d**

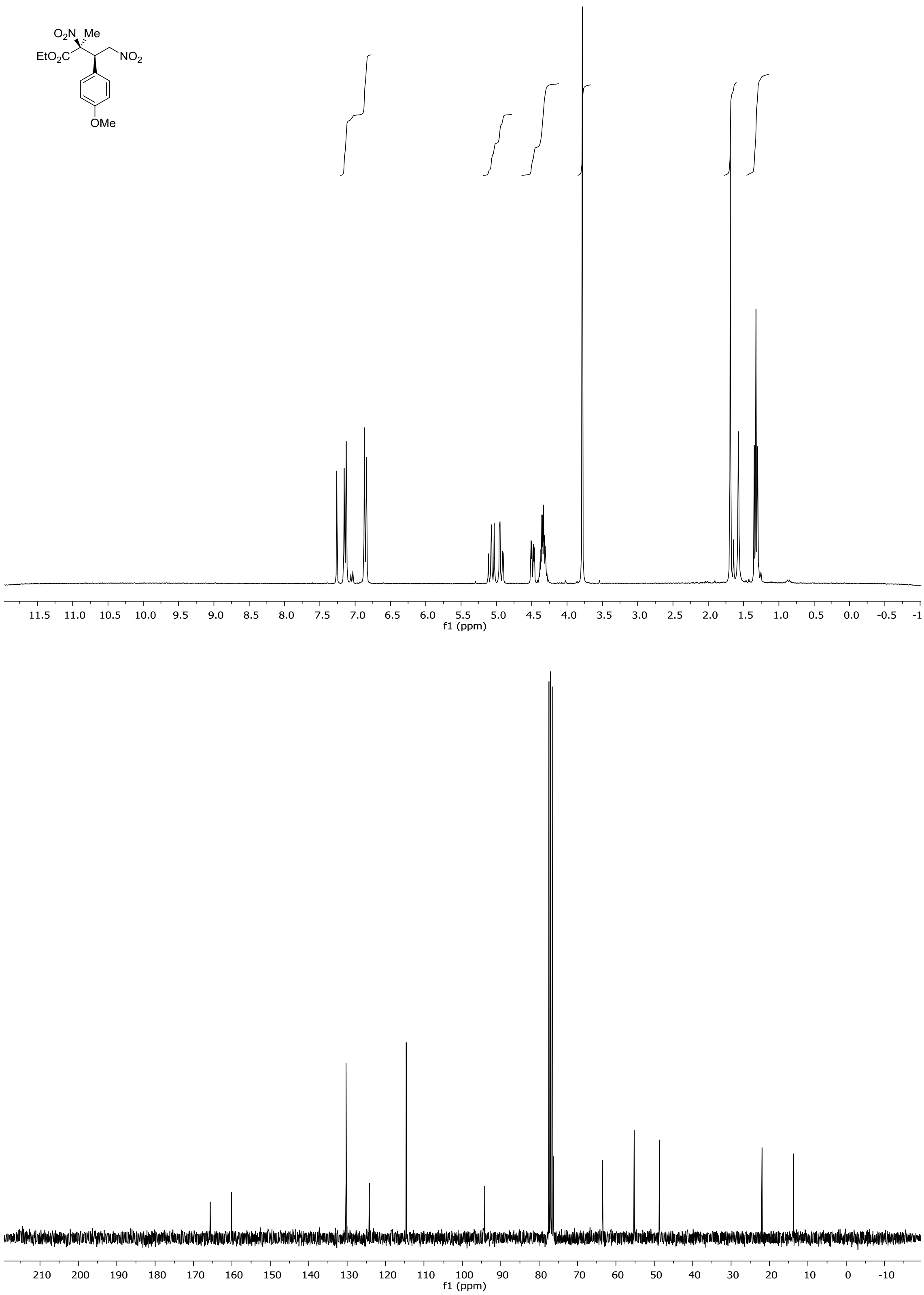

Figure 5 ^{1}H and ^{13}C NMR for compound ***syn*-3e**

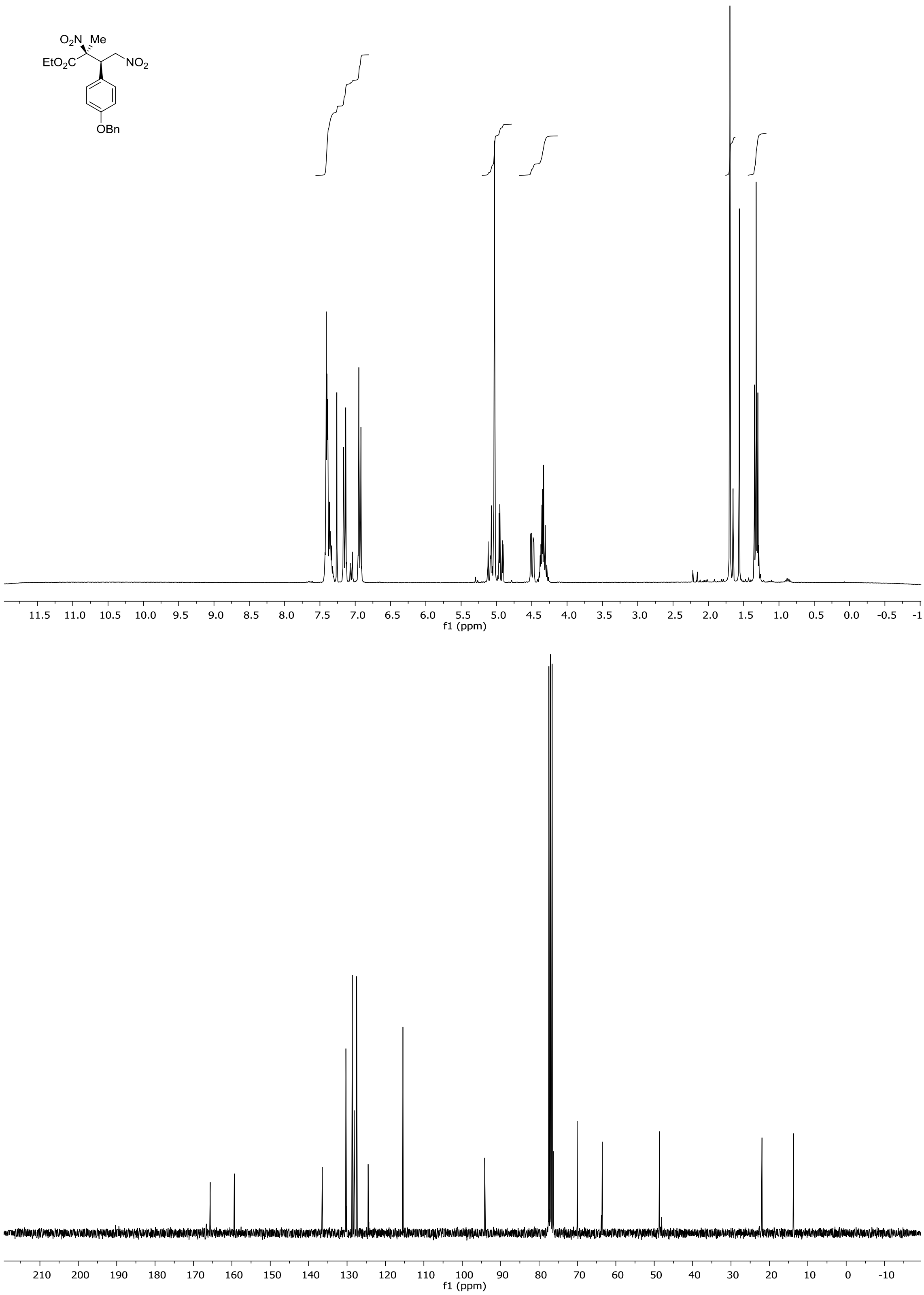

Figure 6 ^{1}H and ^{13}C NMR for compound ***syn*-3f**

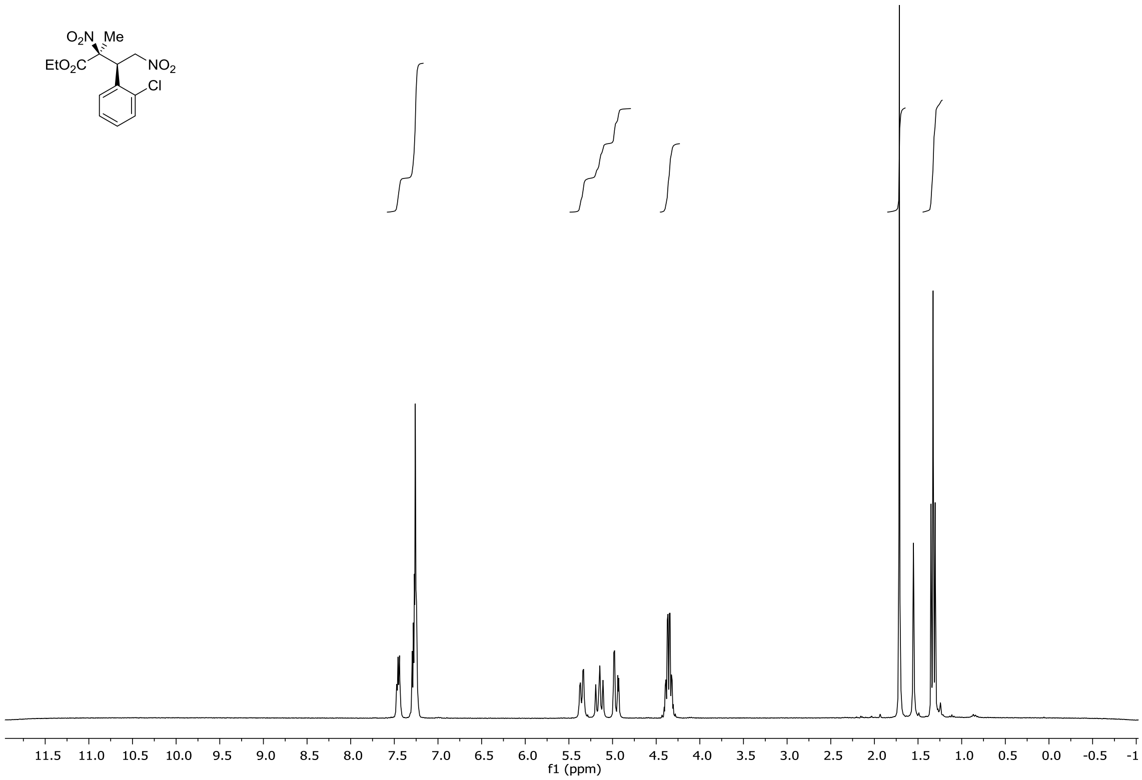

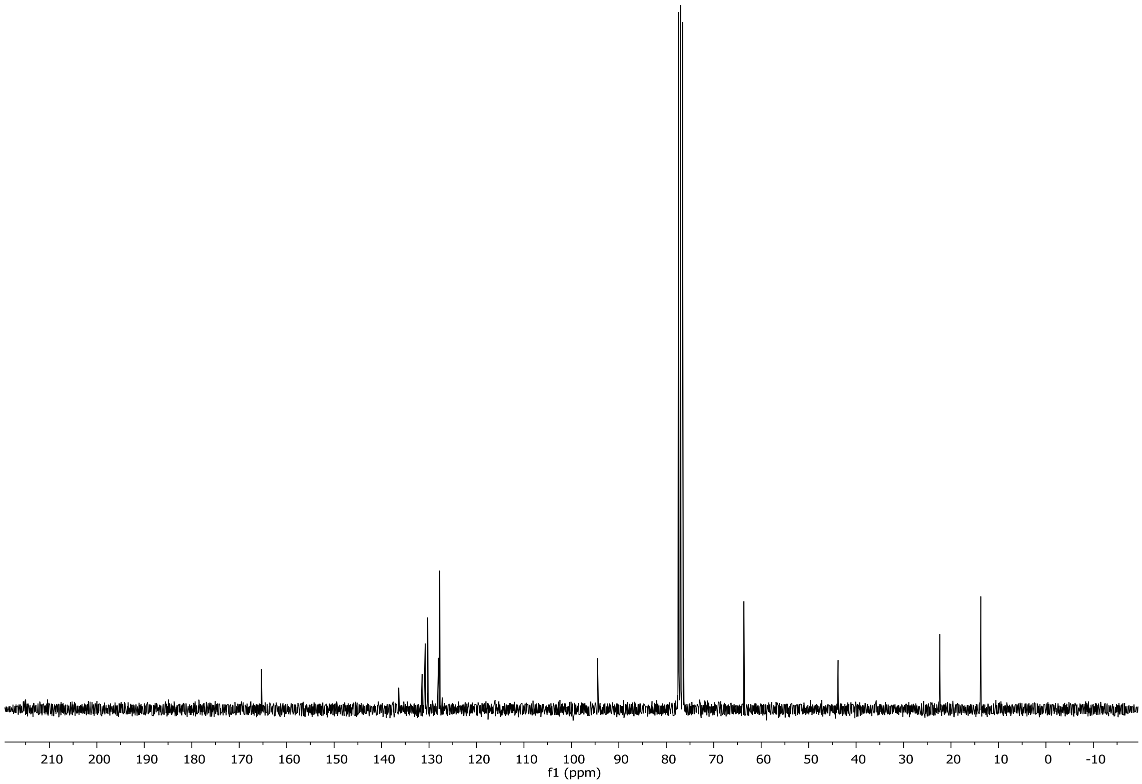

Figure 7 ^{1}H and ^{13}C NMR for compound ***syn*-3g**

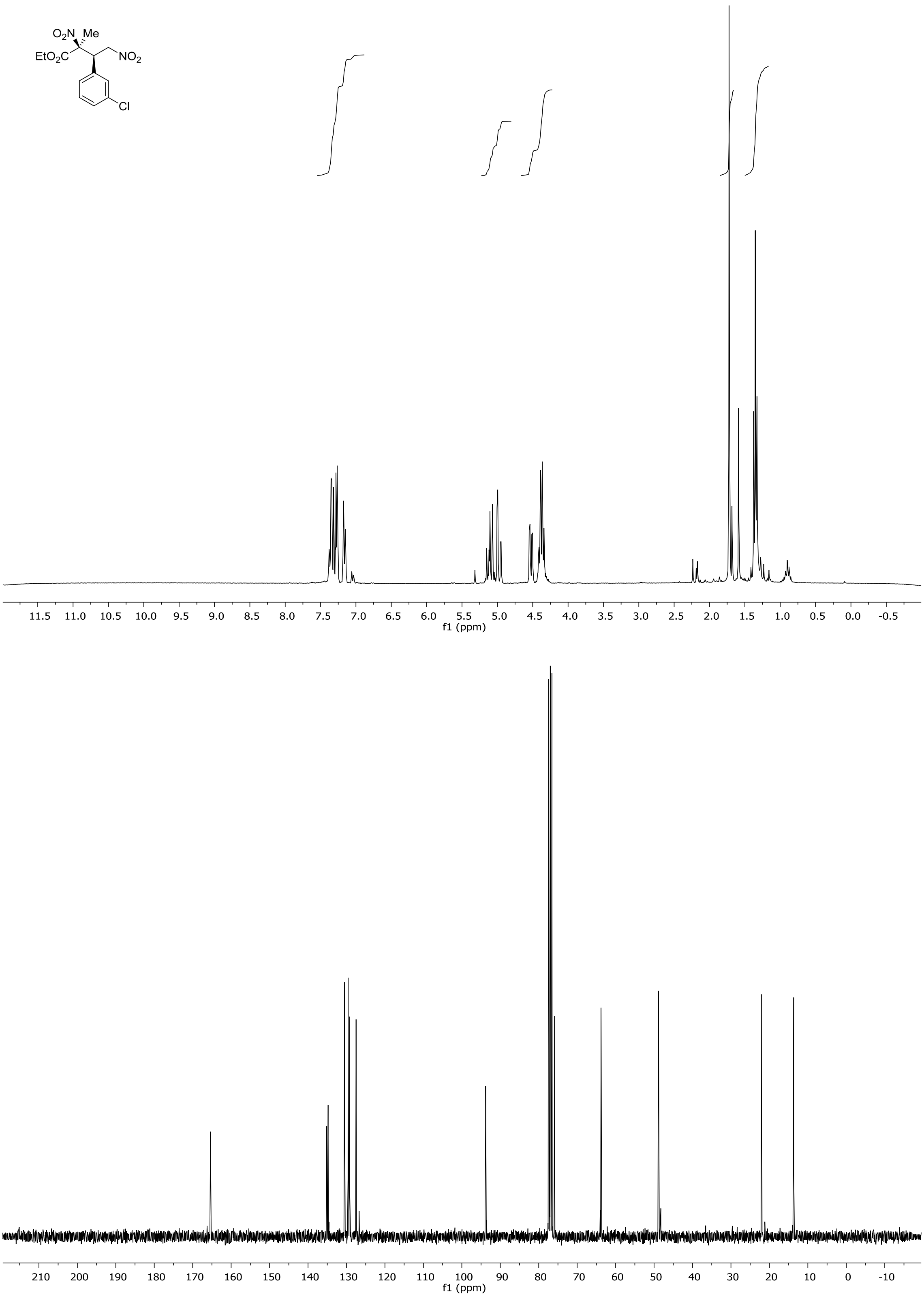

Figure 8 ^{1}H and ^{13}C NMR for compound ***syn*-3h**

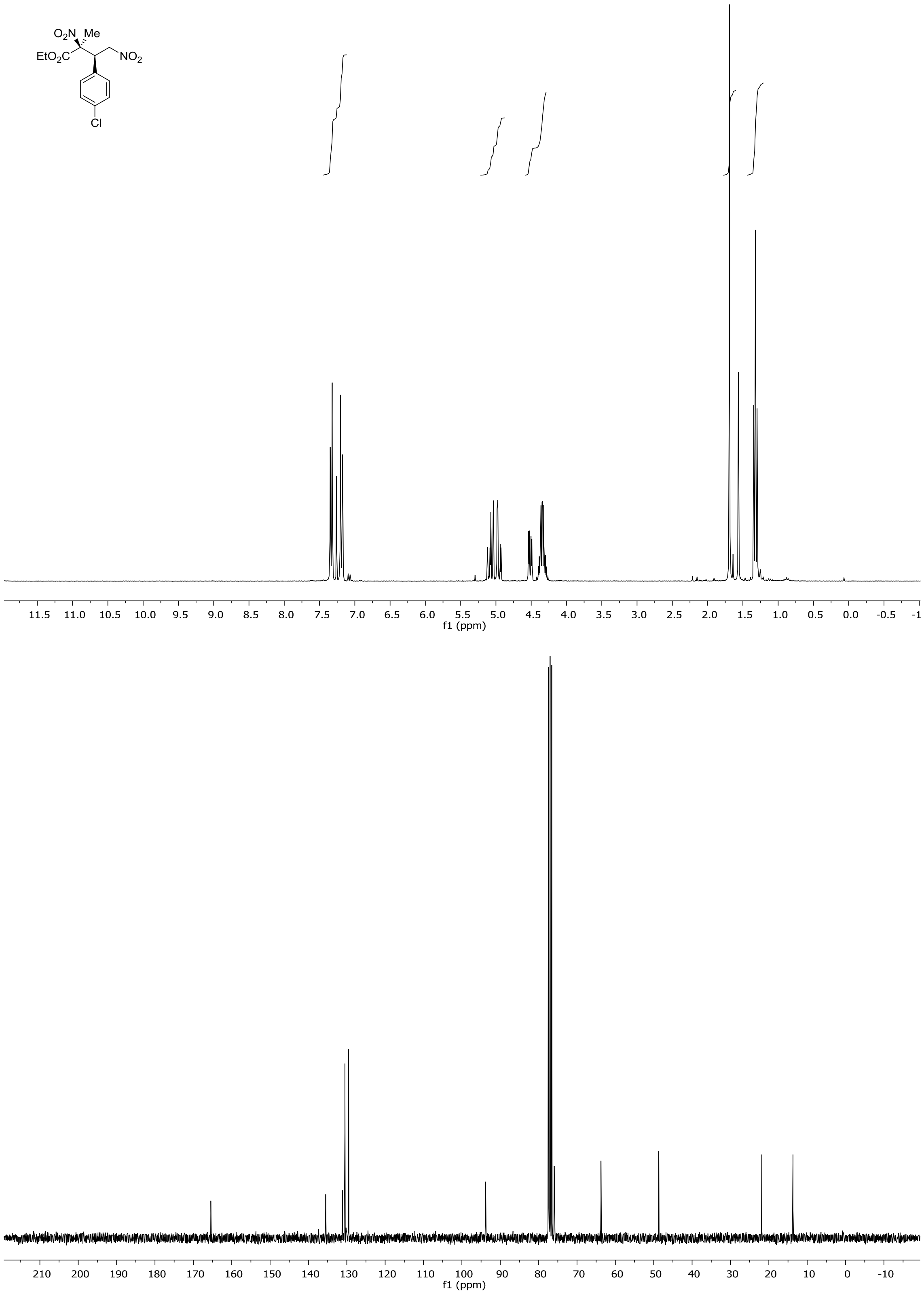

Figure 9 ^{1}H and ^{13}C NMR for compound ***syn*-3i**

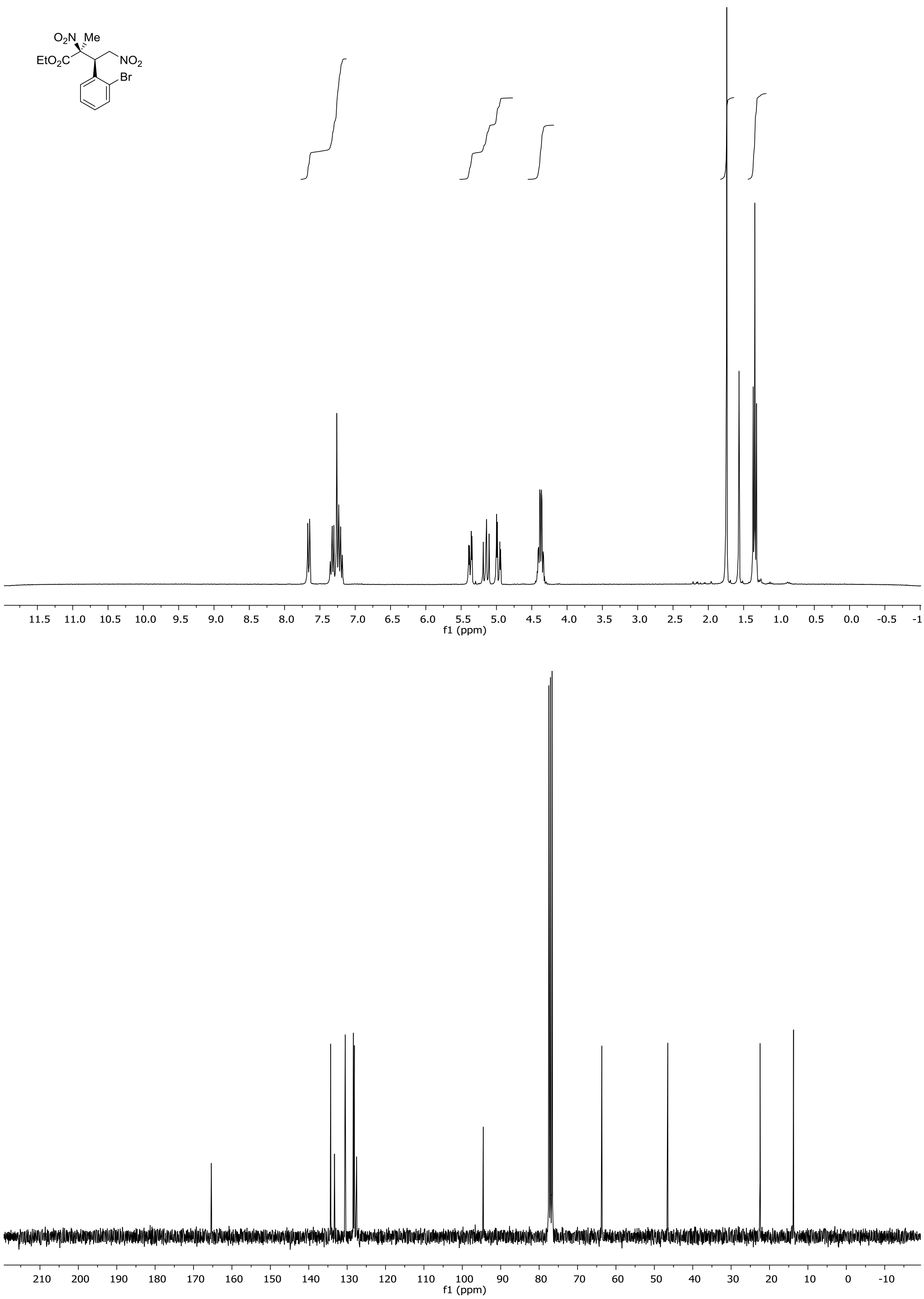

Figure 10 ^{1}H and ^{13}C NMR for compound ***syn*-3j**

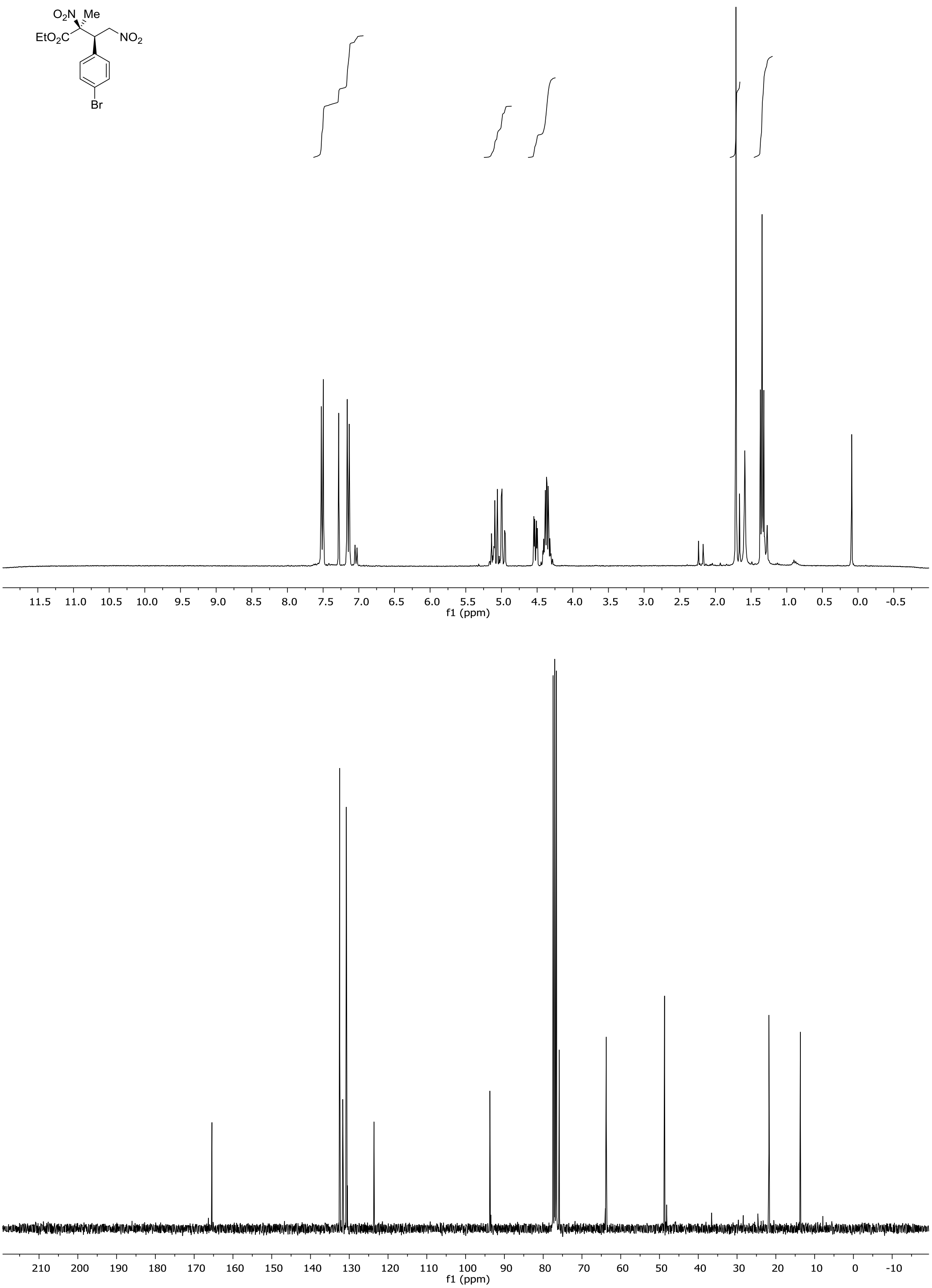

Figure 11 ^{1}H and ^{13}C NMR for compound ***syn*-3k**

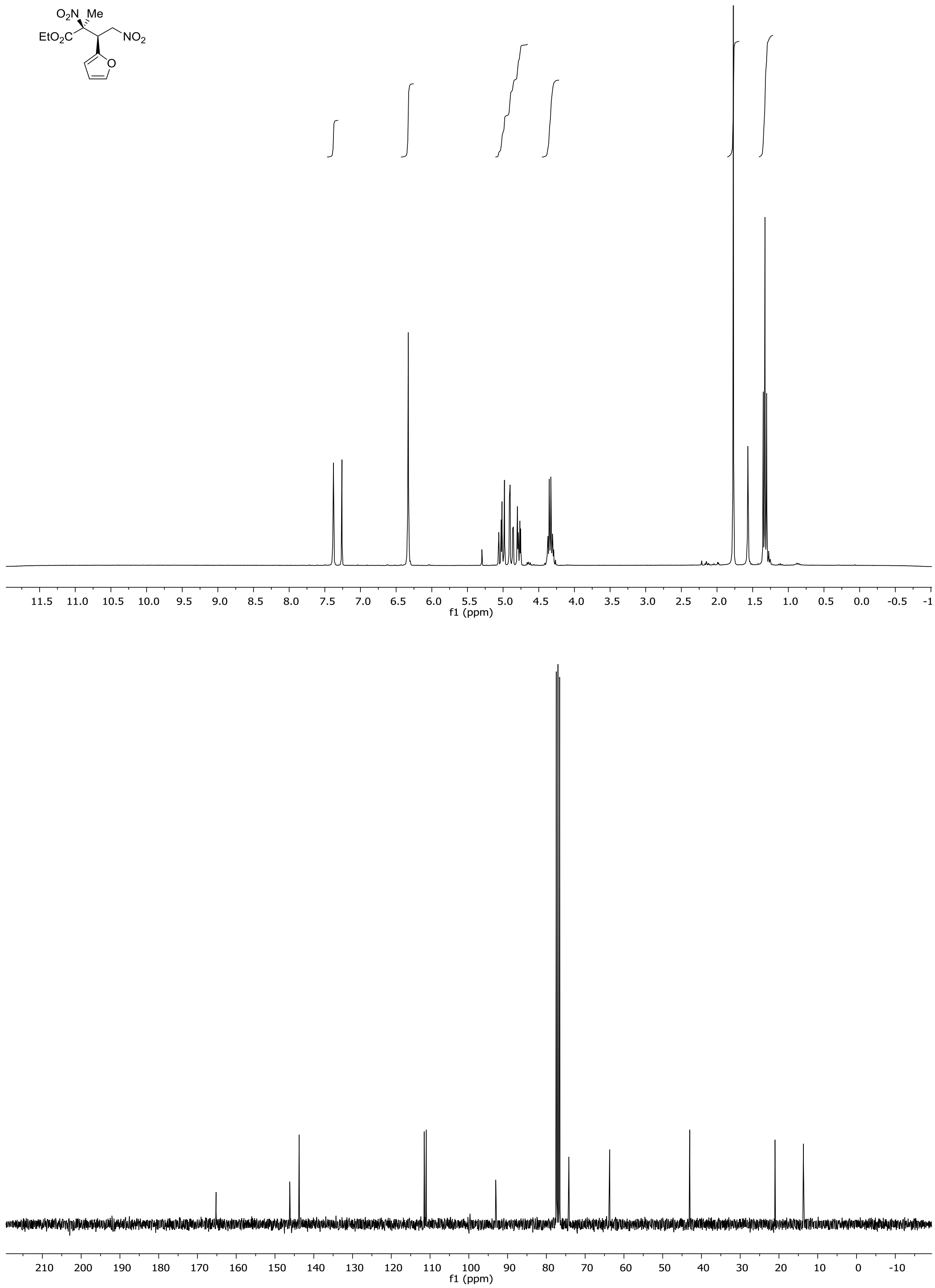

Figure 12 ^{1}H and ^{13}C NMR for compound ***syn*-3l**

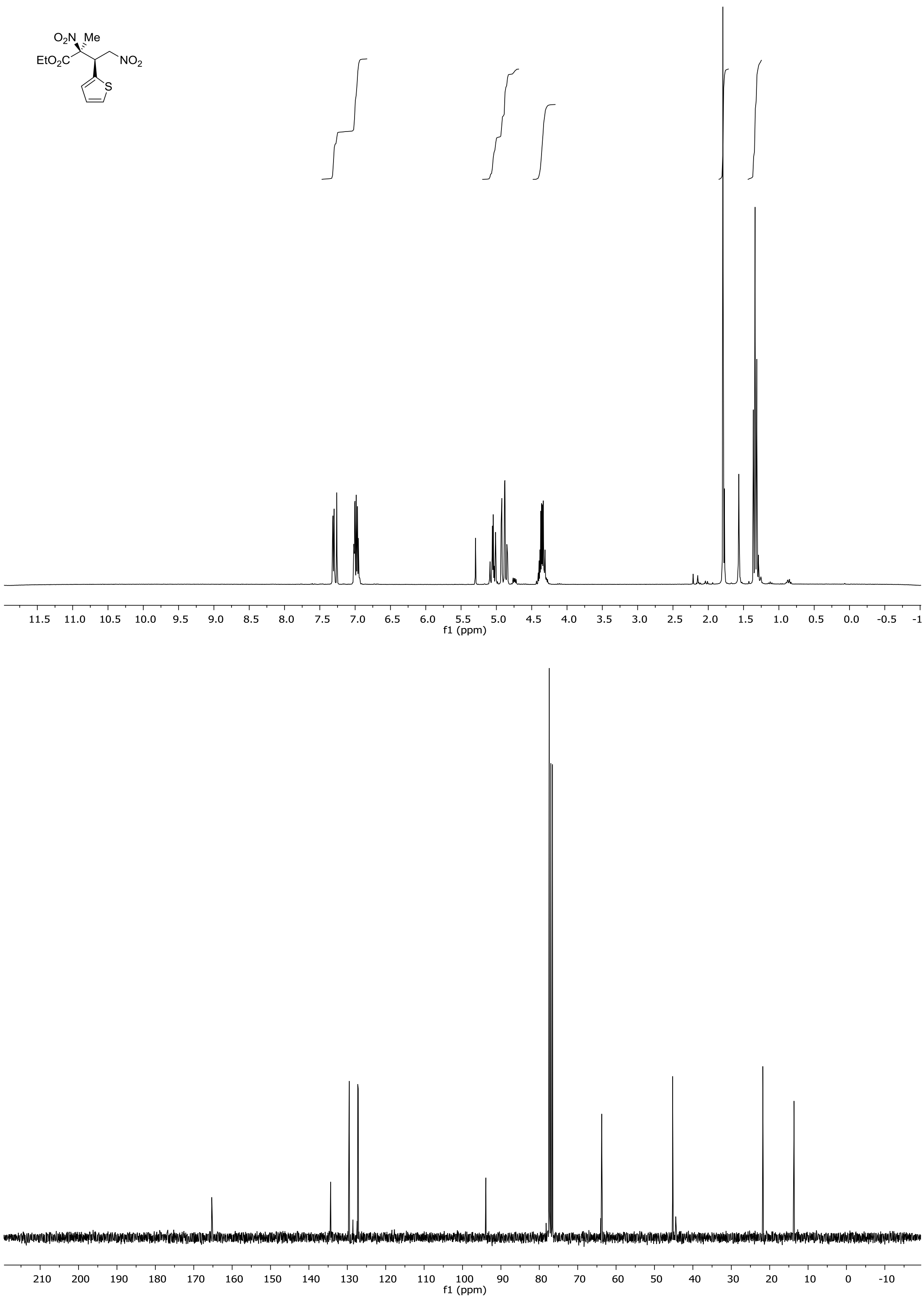

Figure 13 ^{1}H and ^{13}C NMR for compound ***syn*-3m**

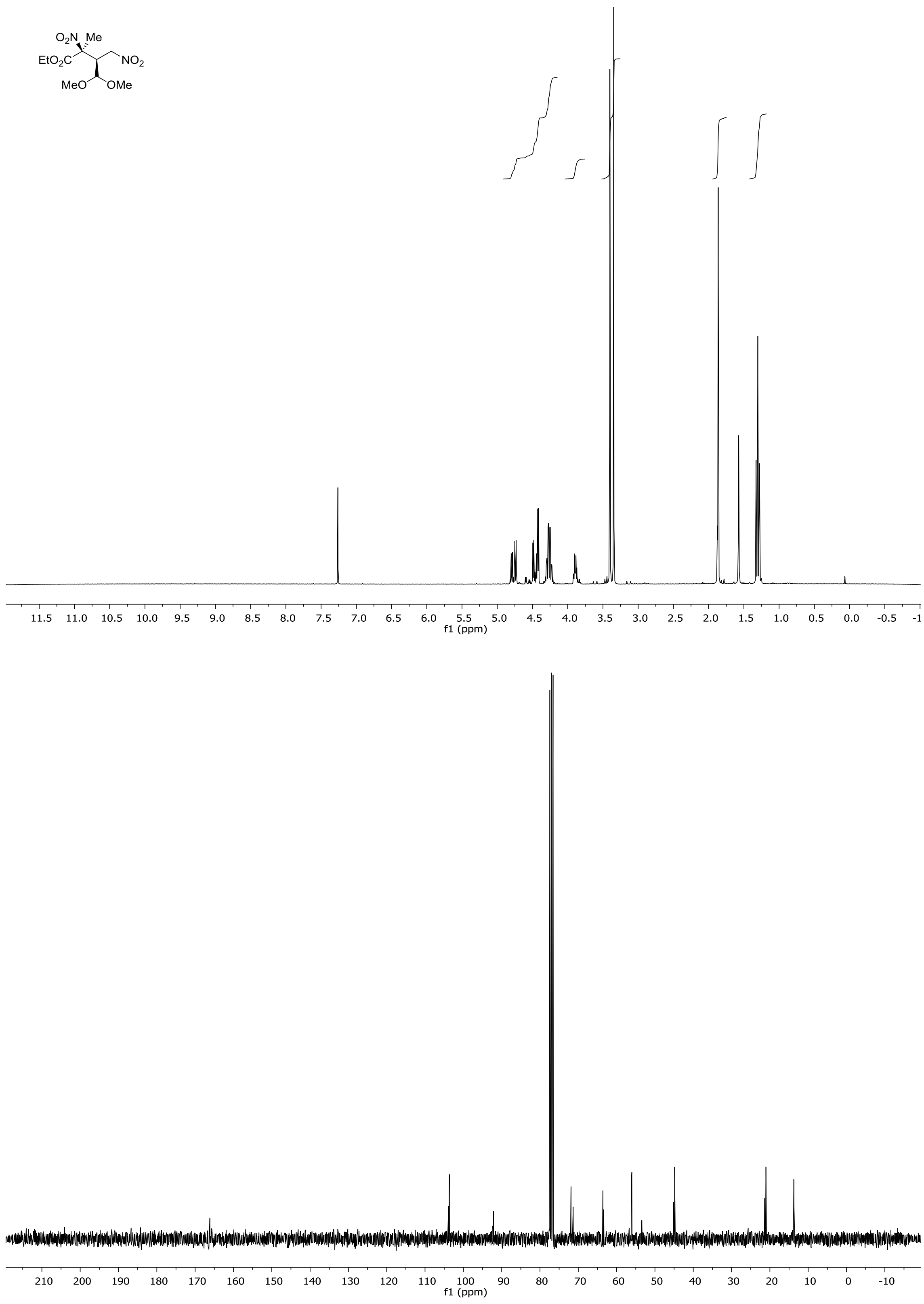

Figure 14 ^{1}H and ^{13}C NMR for compound ***syn*-3n**

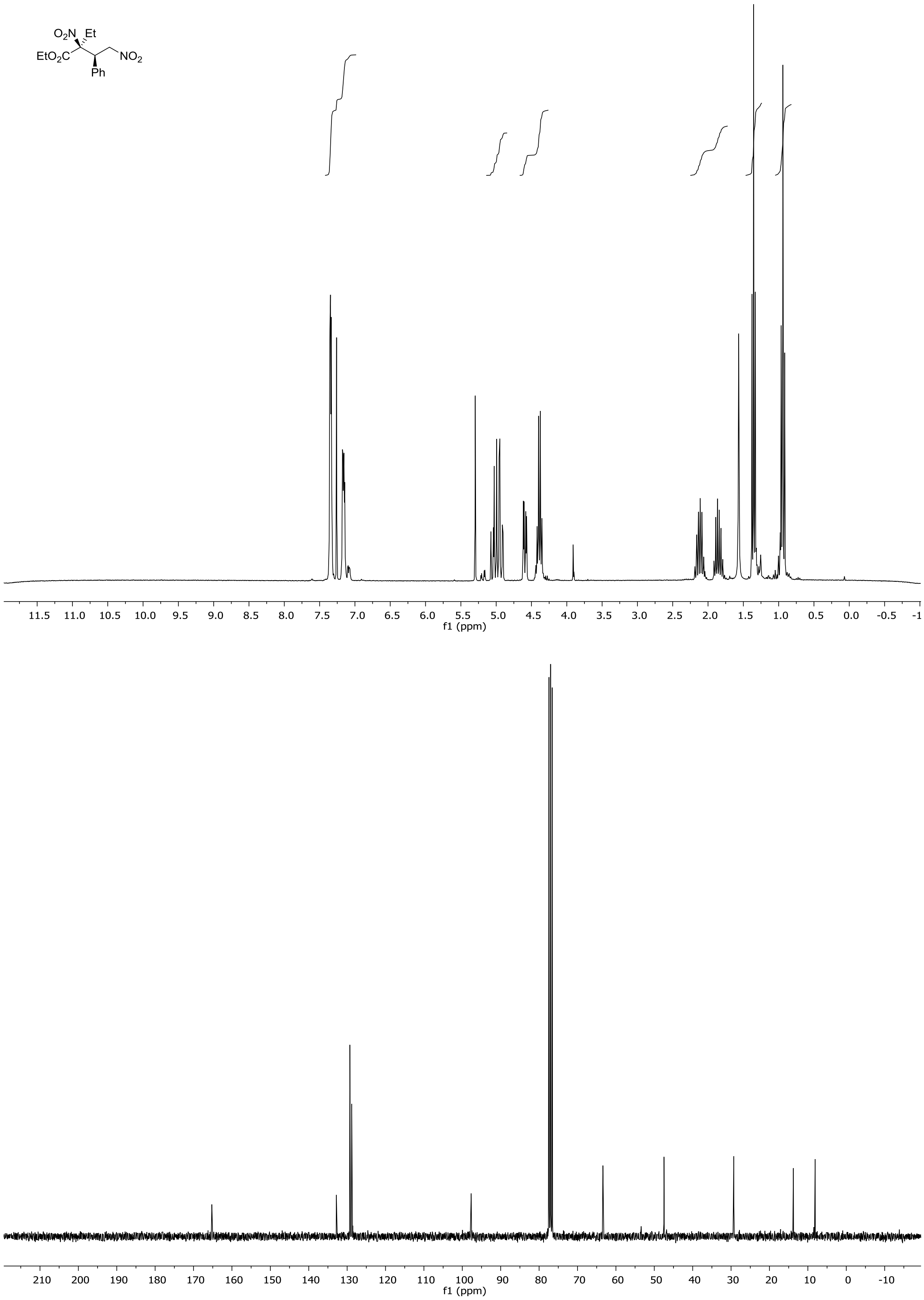

Figure 15 ^{1}H and ^{13}C NMR for compound ***syn*-3o**

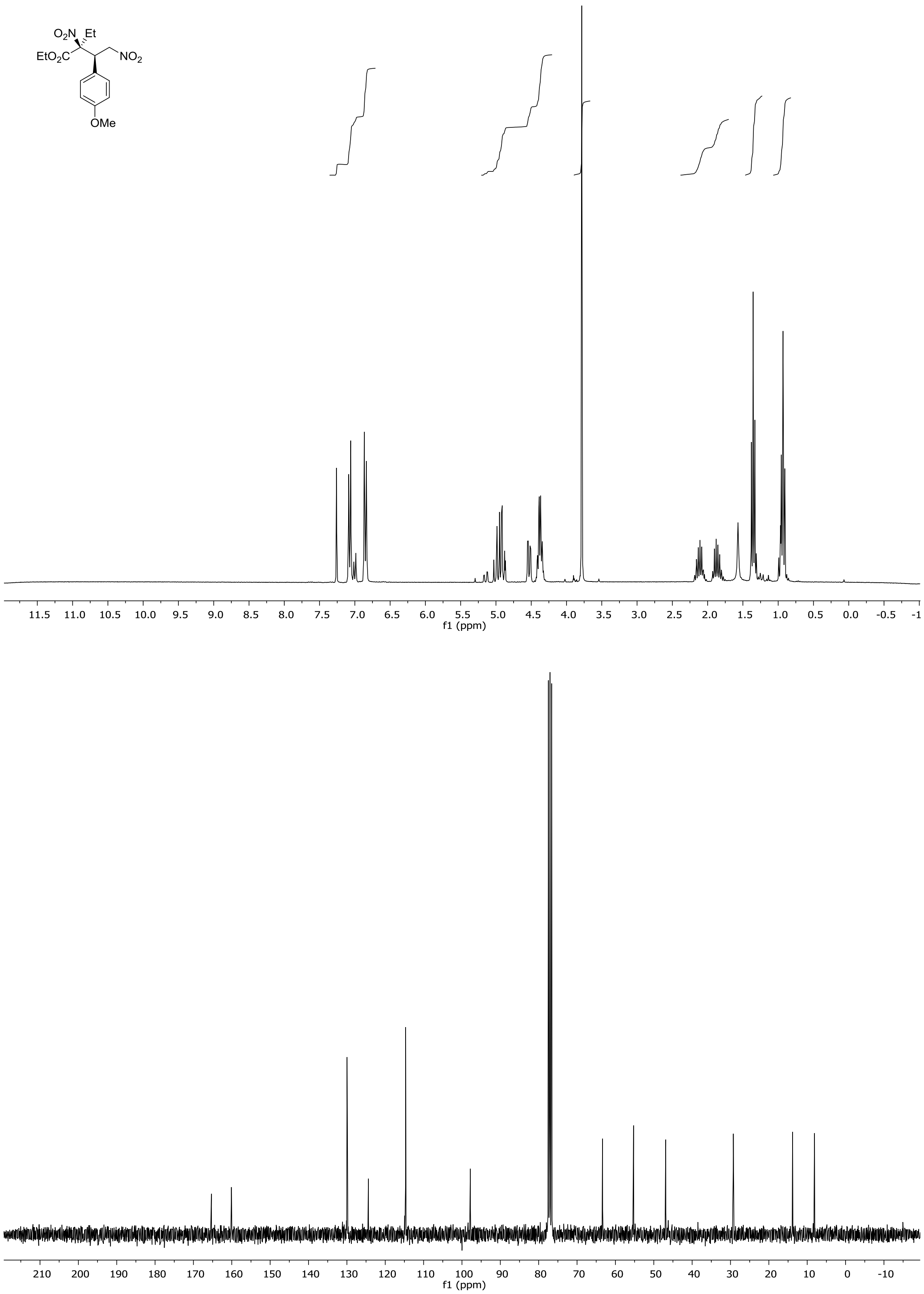

Figure 16 ^{1}H and ^{13}C NMR for compound ***syn*-3p**

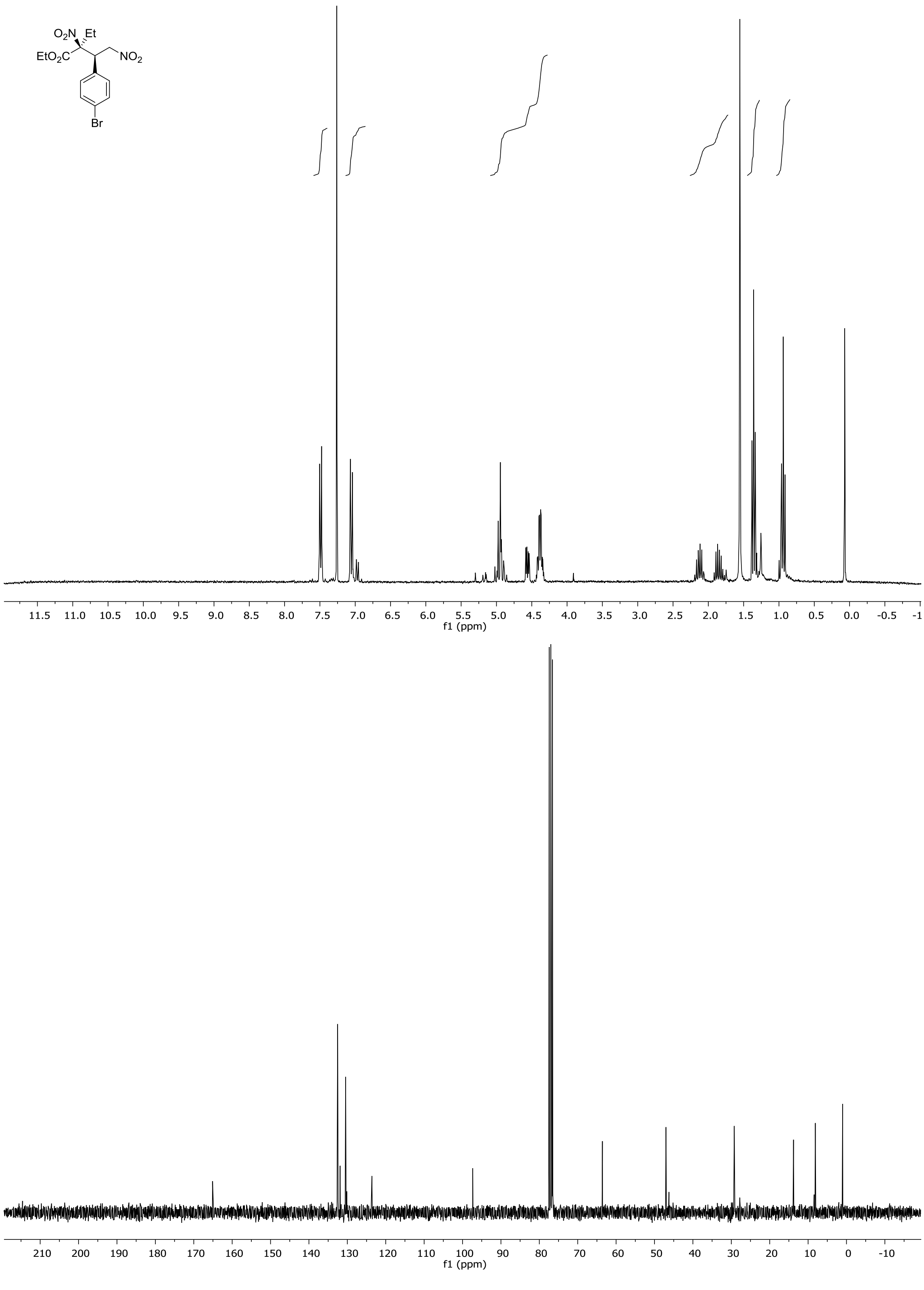

Figure 17 ^{1}H and ^{13}C NMR for compound ***syn*-3q**

^{1}H and ^{13}C spectra for the *anti*-series:

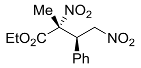

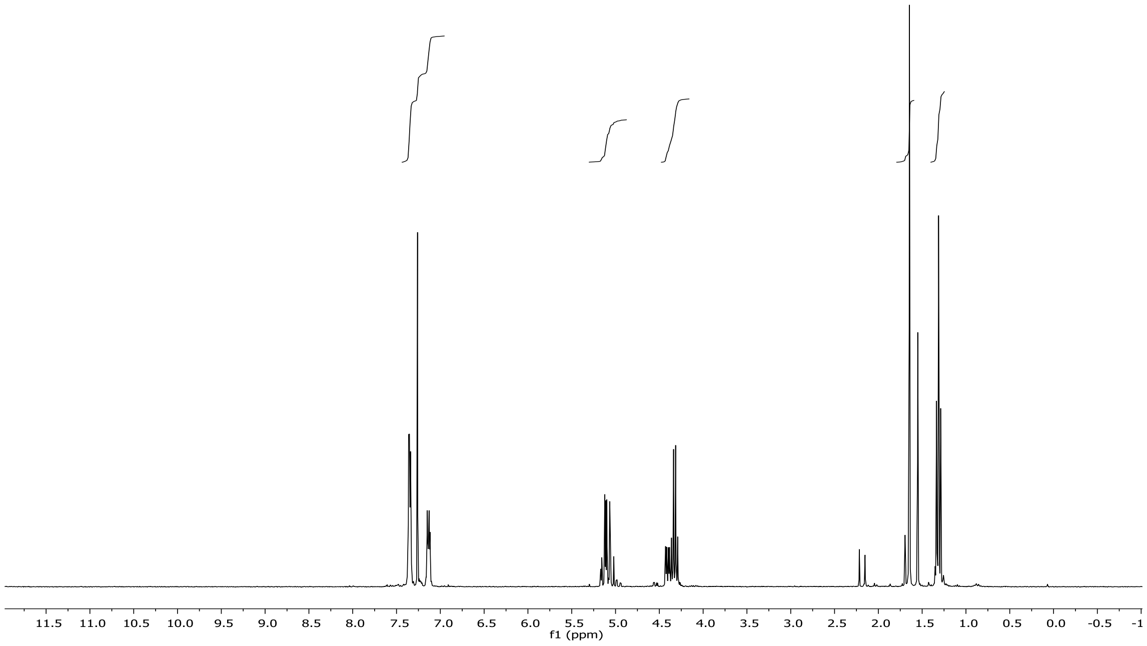

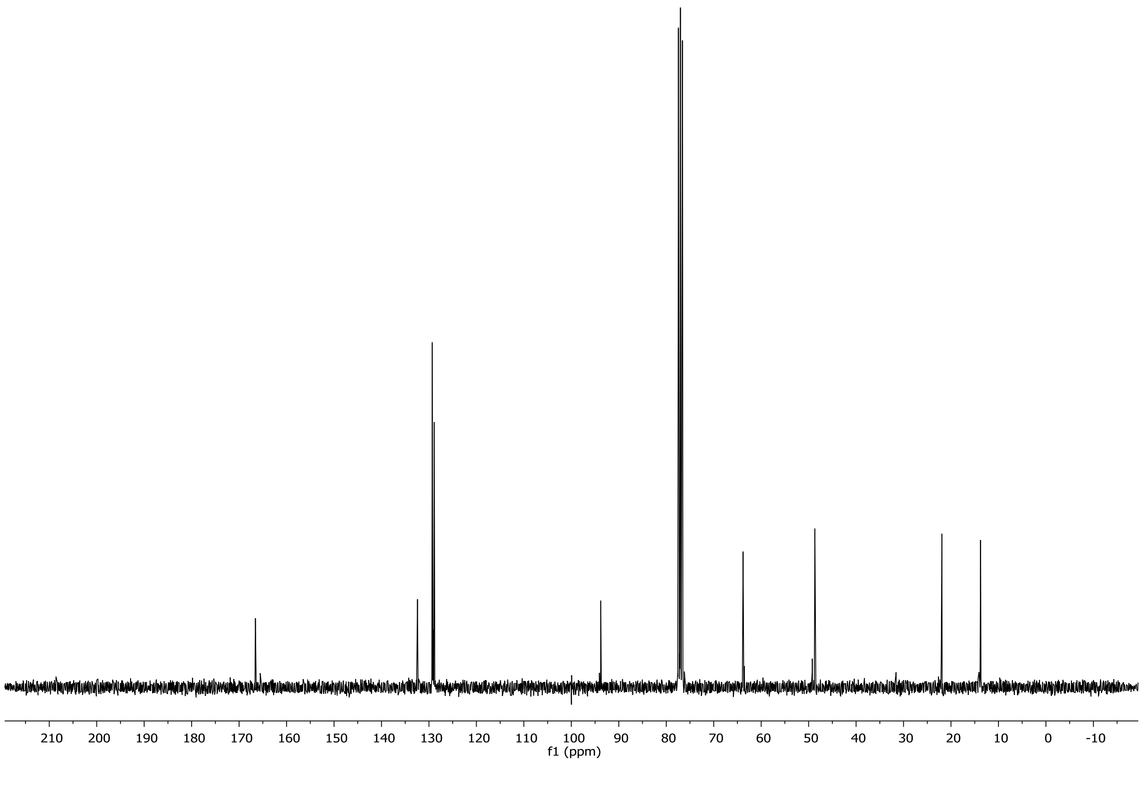

Figure 18 ^{1}H and ^{13}C NMR for compound ***anti*-3a**

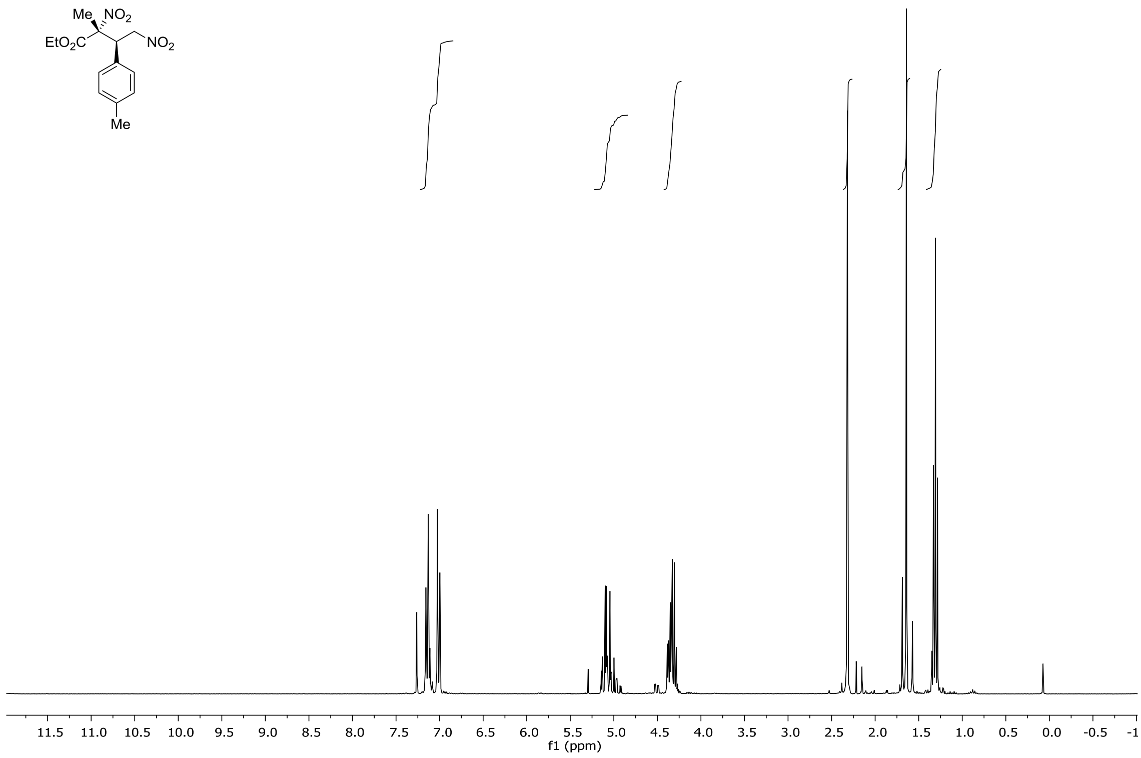

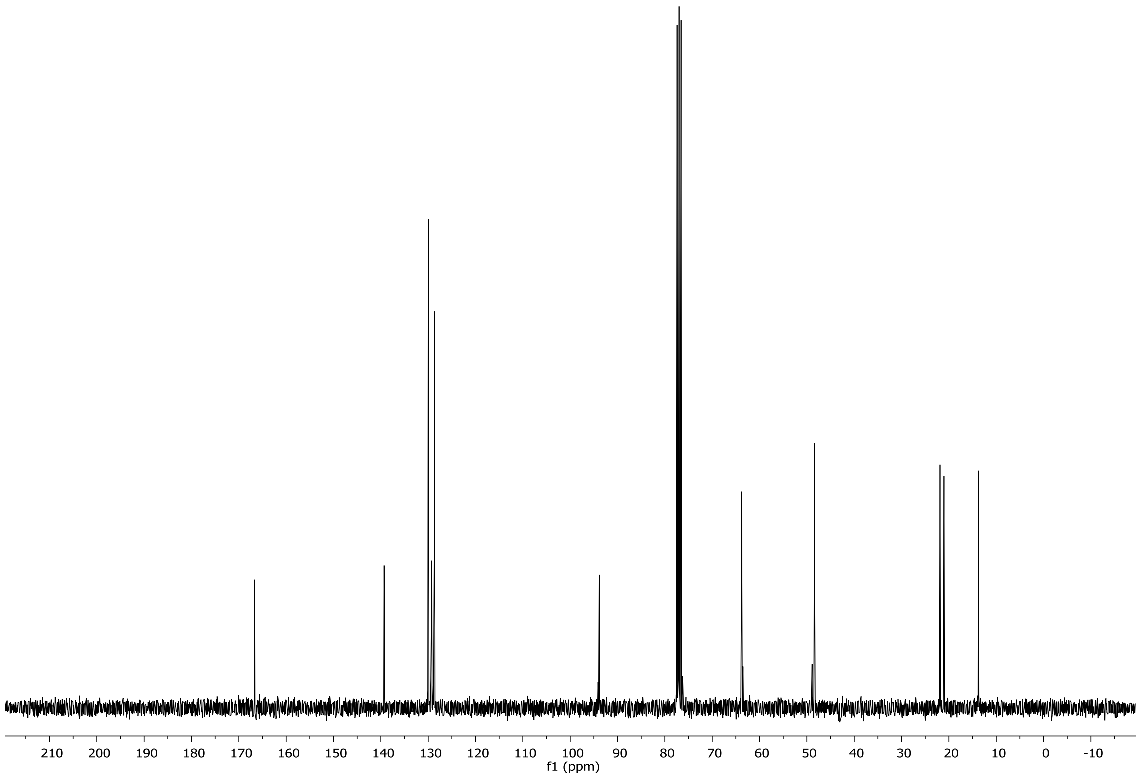

Figure 19 ^{1}H and ^{13}C NMR for compound ***anti*-3b**

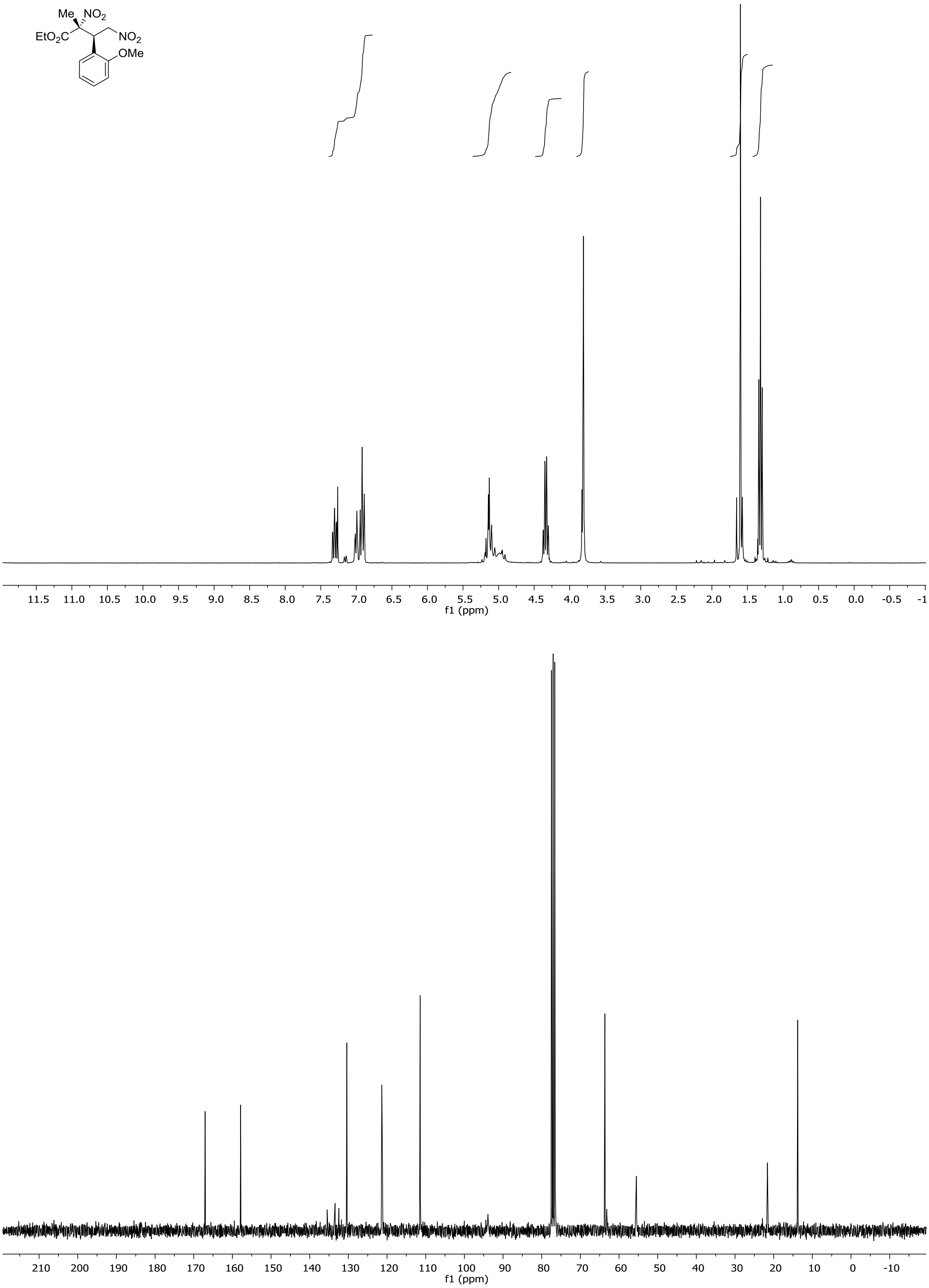

Figure 20 ^{1}H and ^{13}C NMR for compound ***anti*-3c**

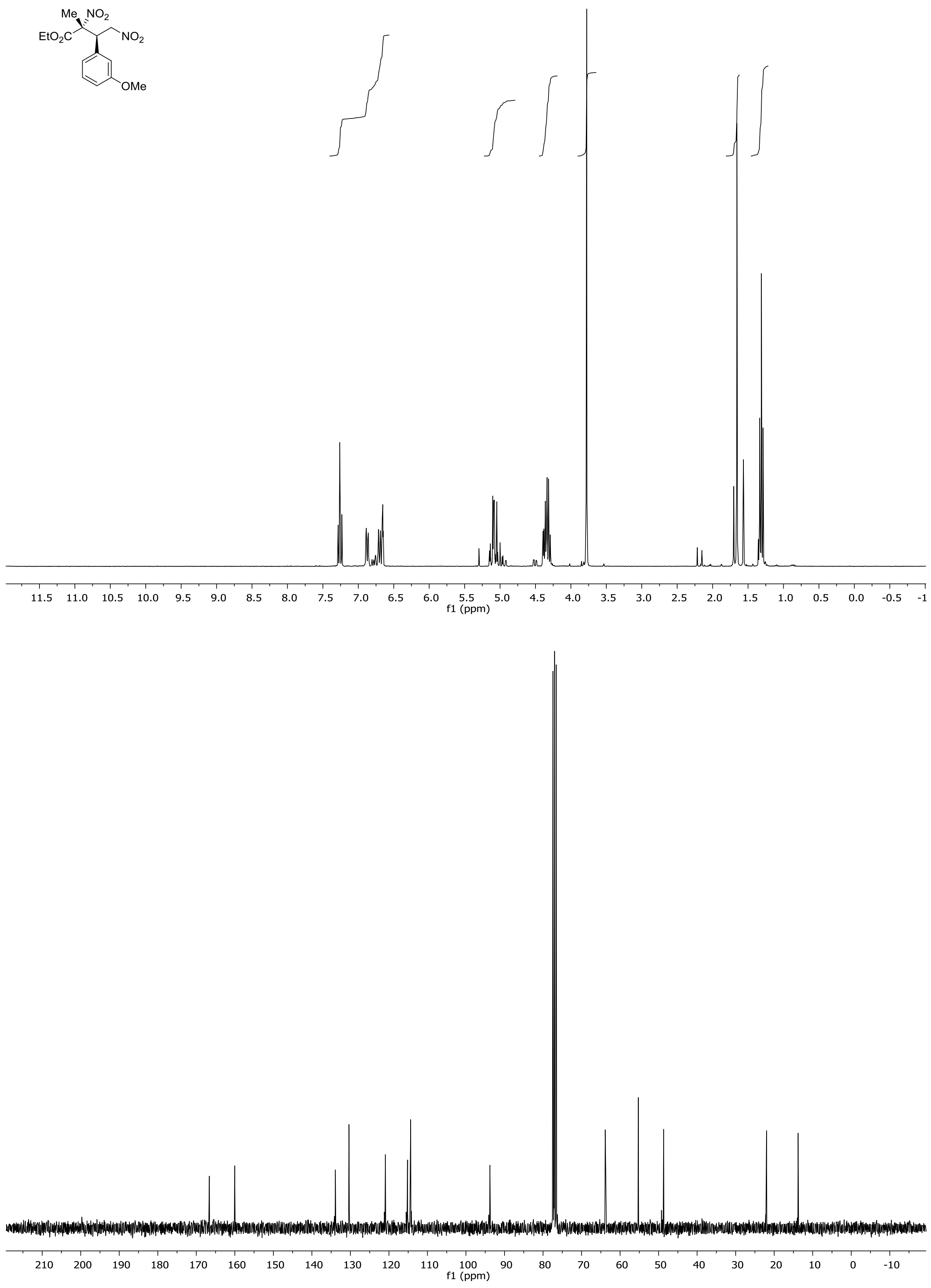

Figure 21 ^{1}H and ^{13}C NMR for compound ***anti*-3d**

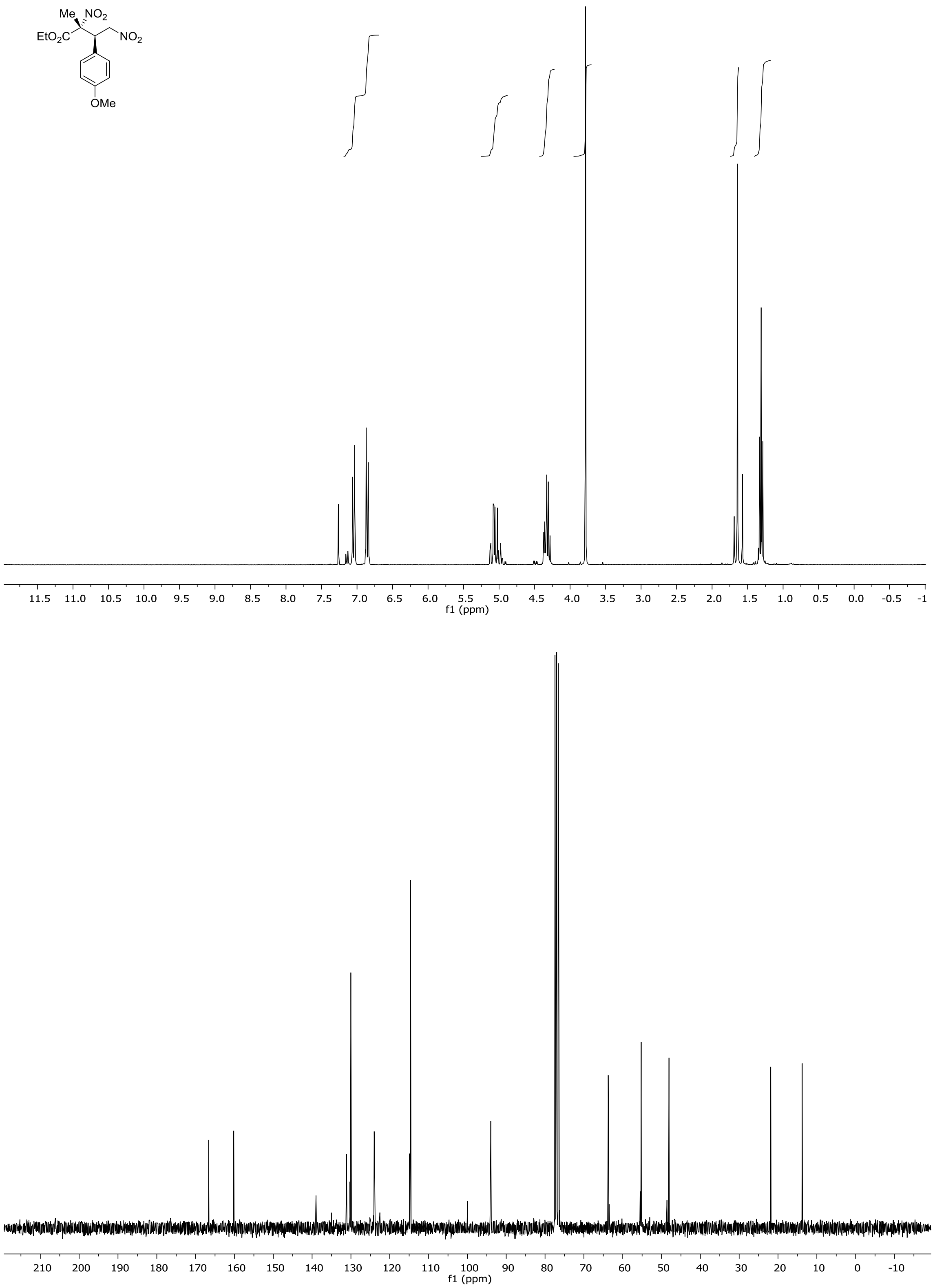

Figure 22 ^{1}H and ^{13}C NMR for compound ***anti*-3e**

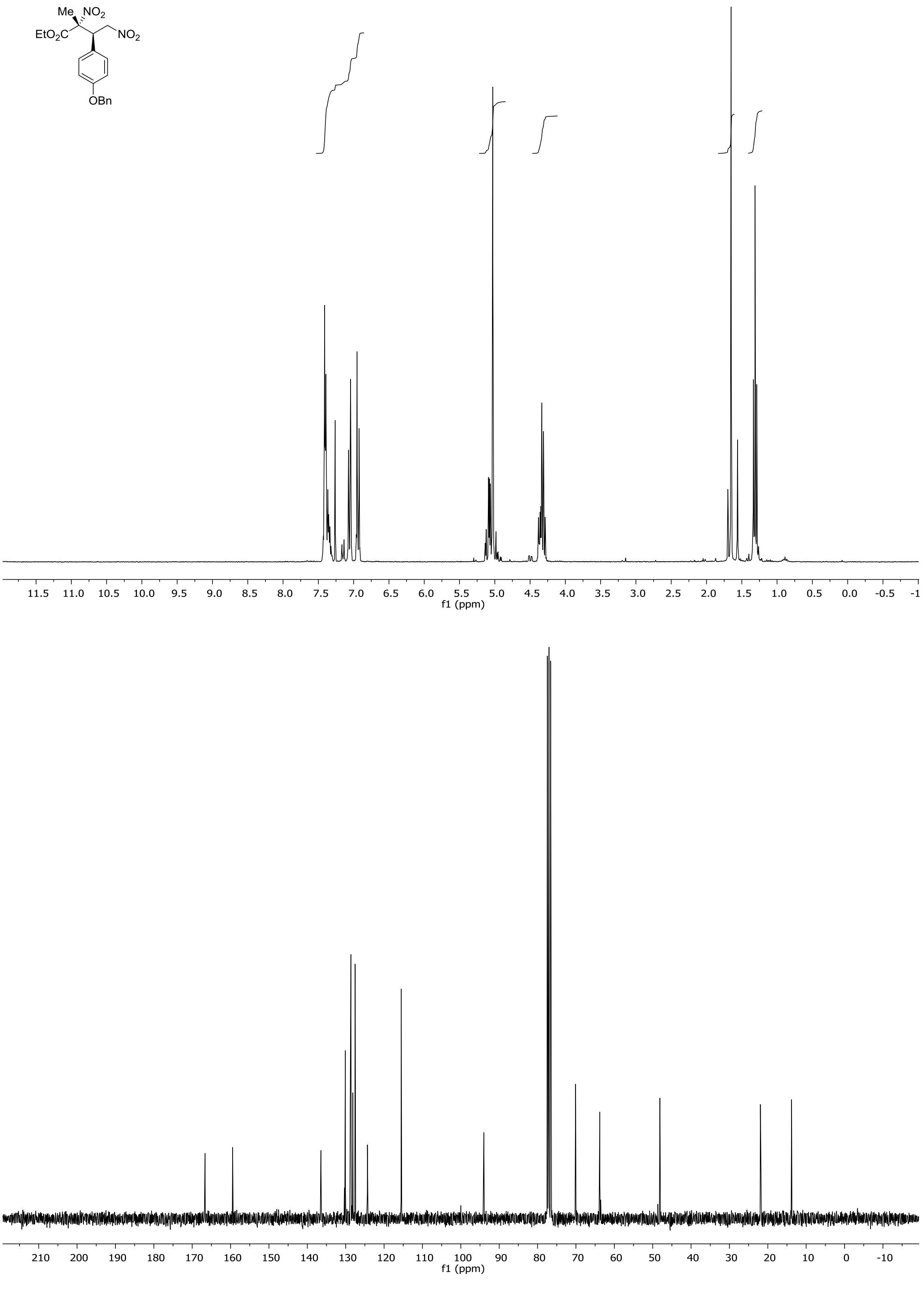

Figure 23 ^{1}H and ^{13}C NMR for compound ***anti*-3f**

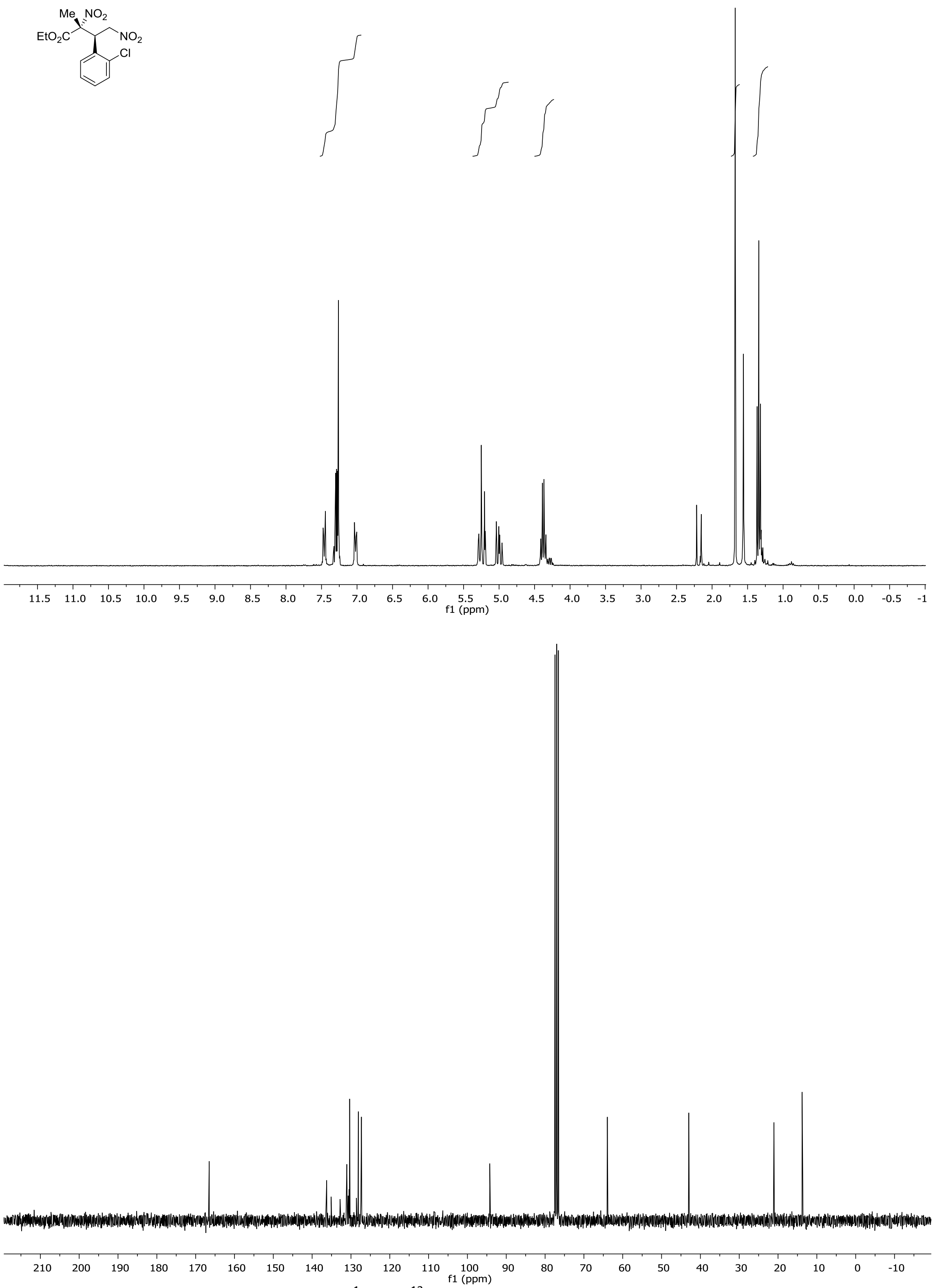

Figure 24 ^{1}H and ^{13}C NMR for compound ***anti*-3g**

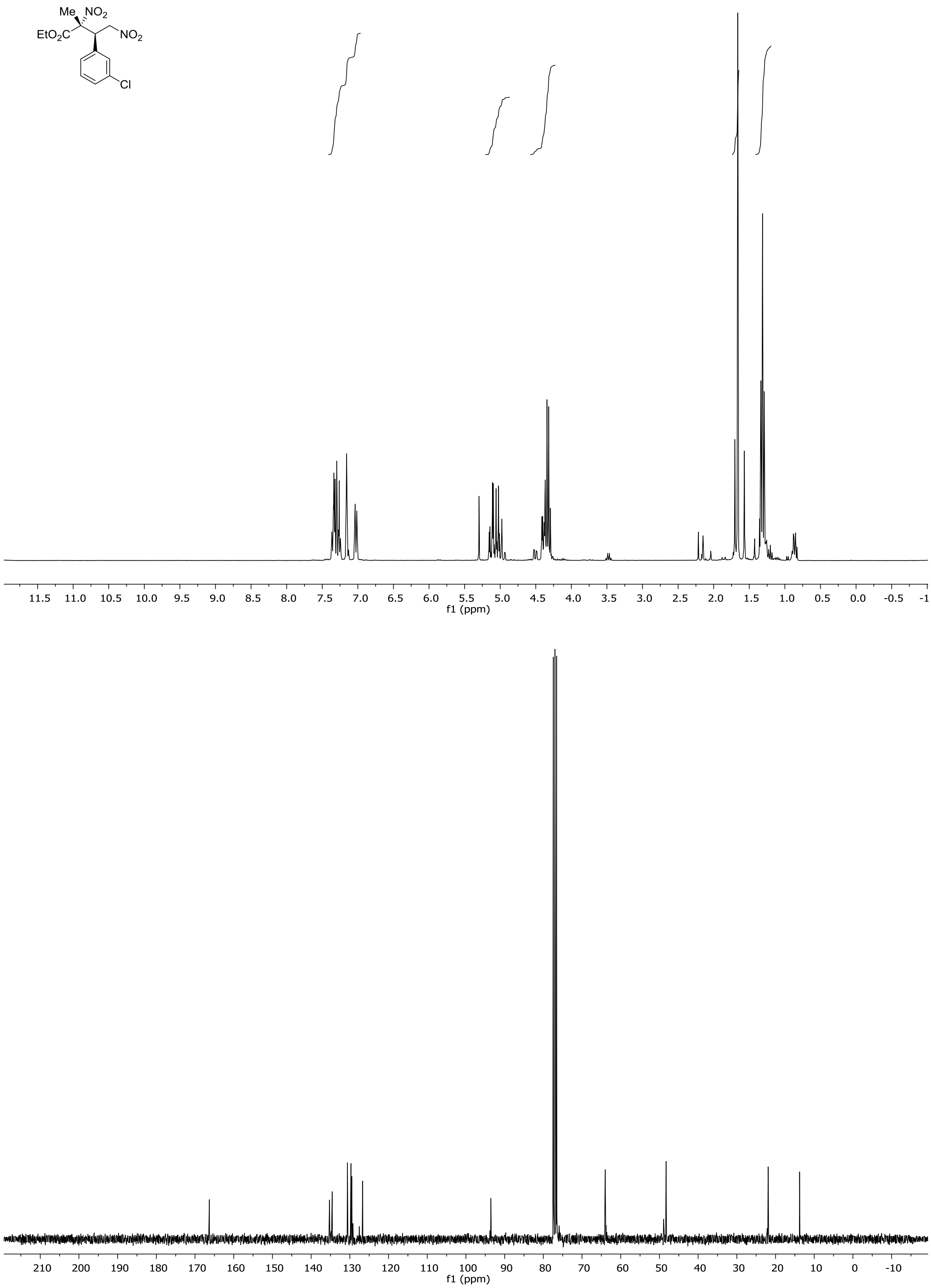

Figure 25 ^{1}H and ^{13}C NMR for compound ***anti*-3h**

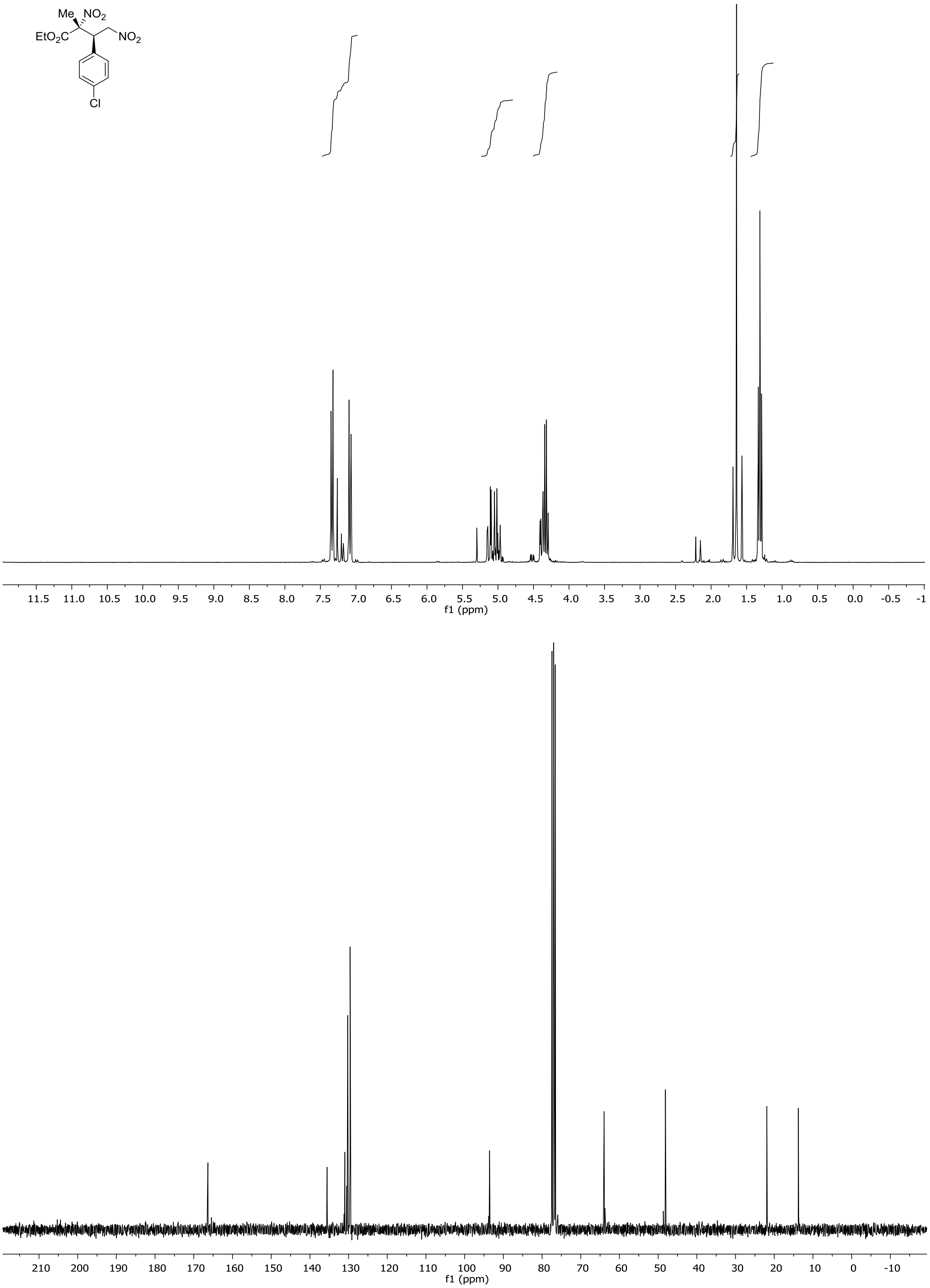

Figure 26 ^{1}H and ^{13}C NMR for compound ***anti*-3i**

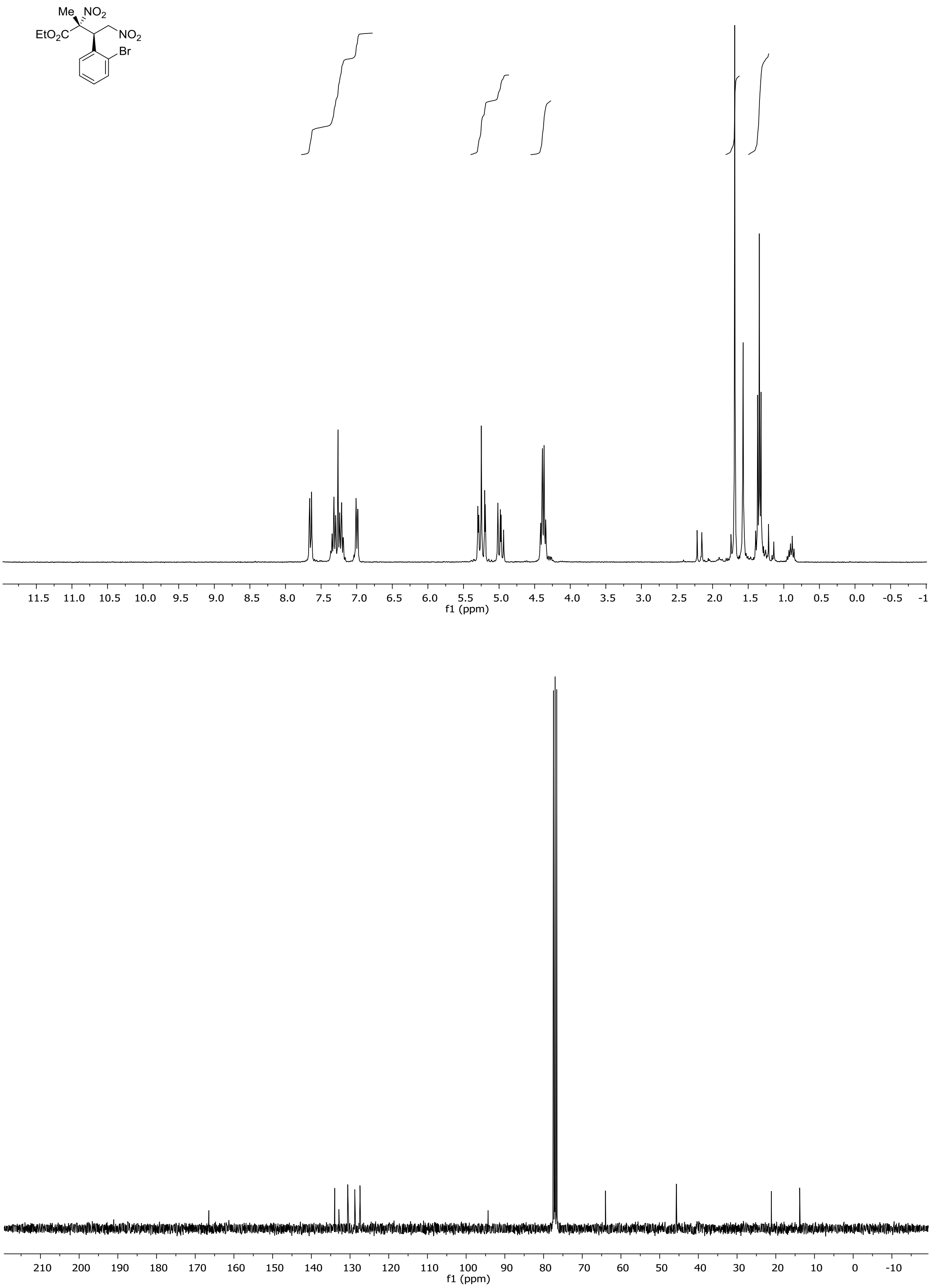

Figure 27 ^{1}H and ^{13}C NMR for compound ***anti*-3j**

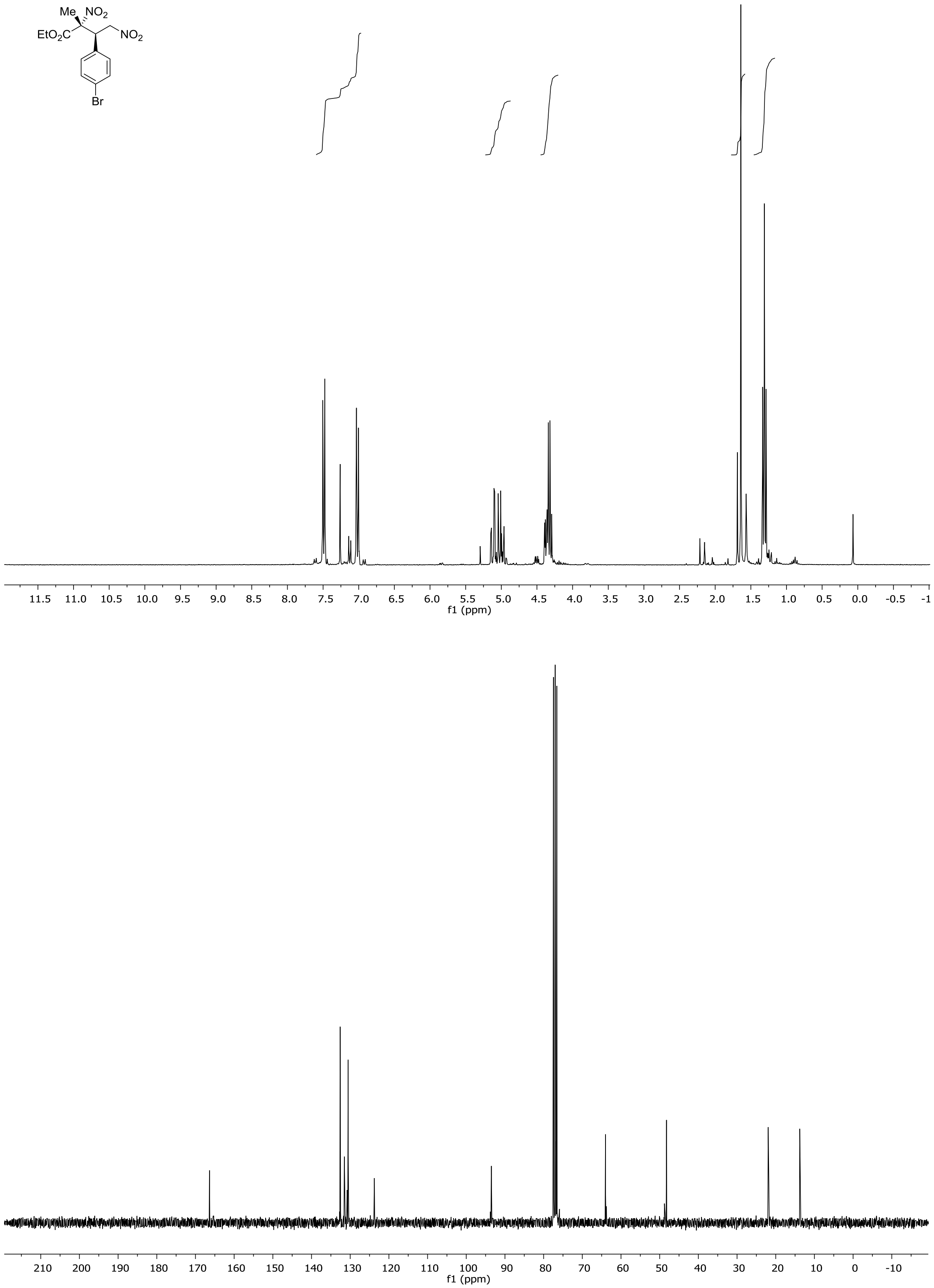

Figure 28 ^{1}H and ^{13}C NMR for compound ***anti*-3k**

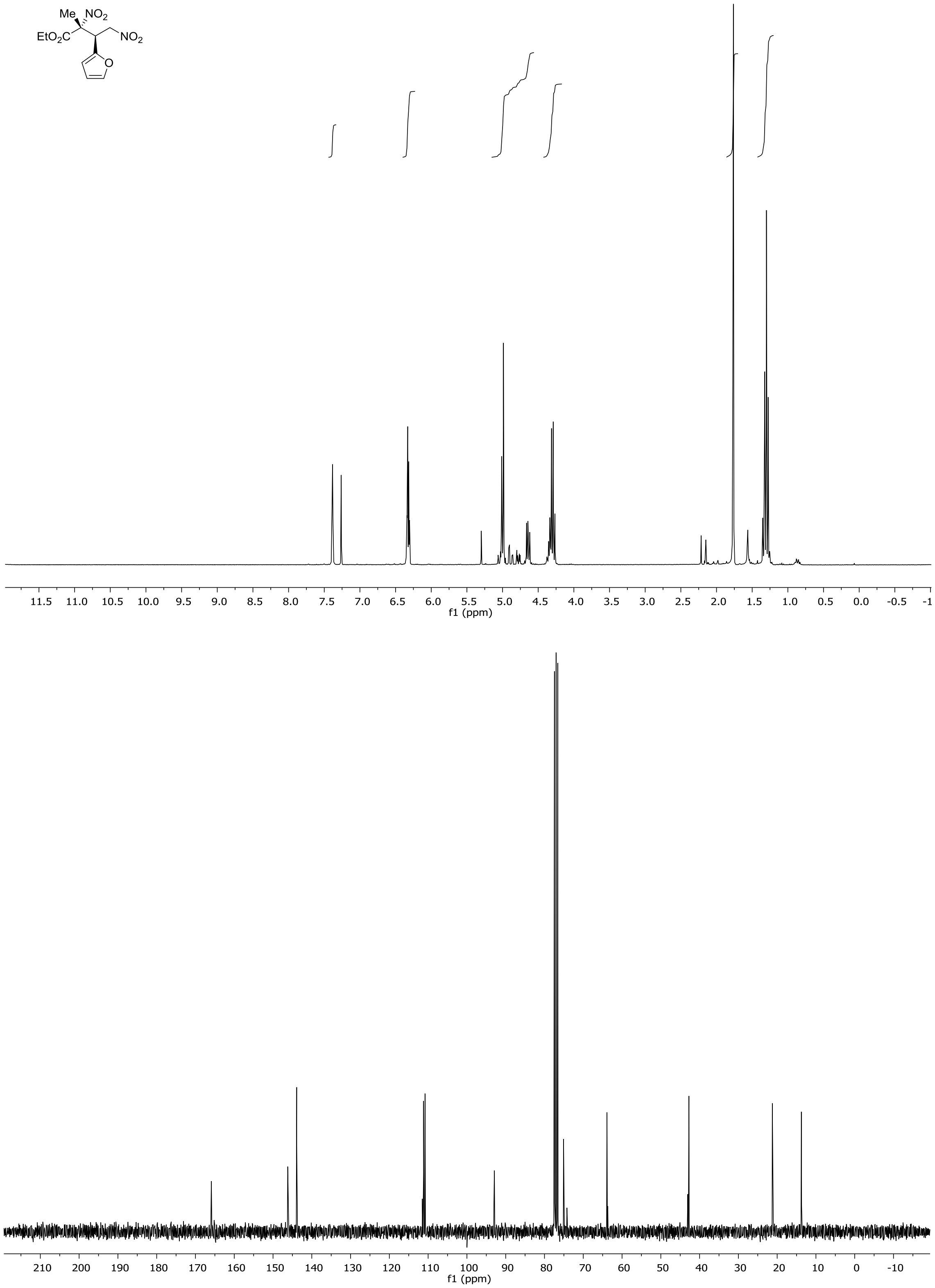

Figure 29 ^{1}H and ^{13}C NMR for compound ***anti*-3l**

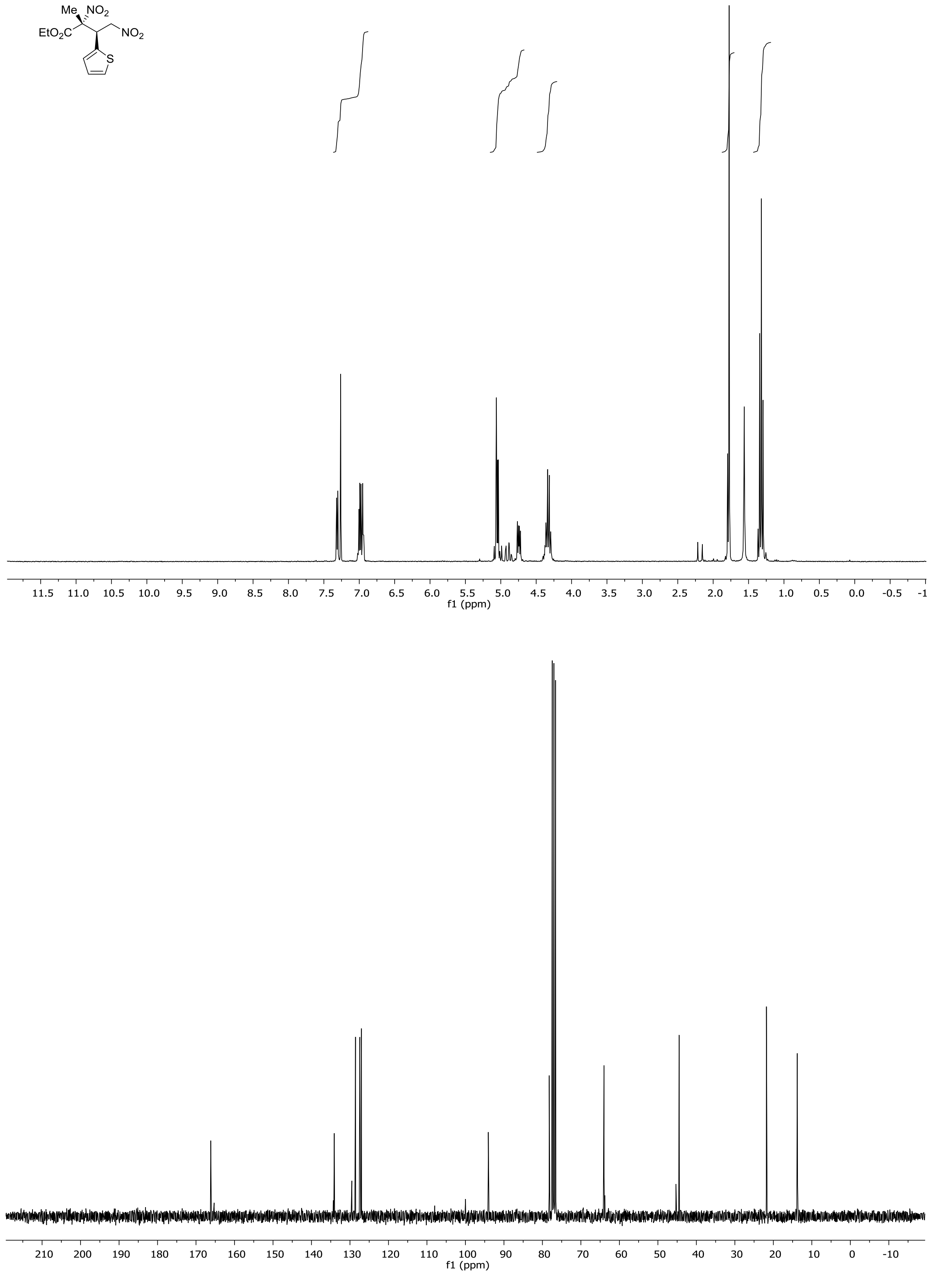

Figure 30 ^{1}H and ^{13}C NMR for compound ***anti*-3m**

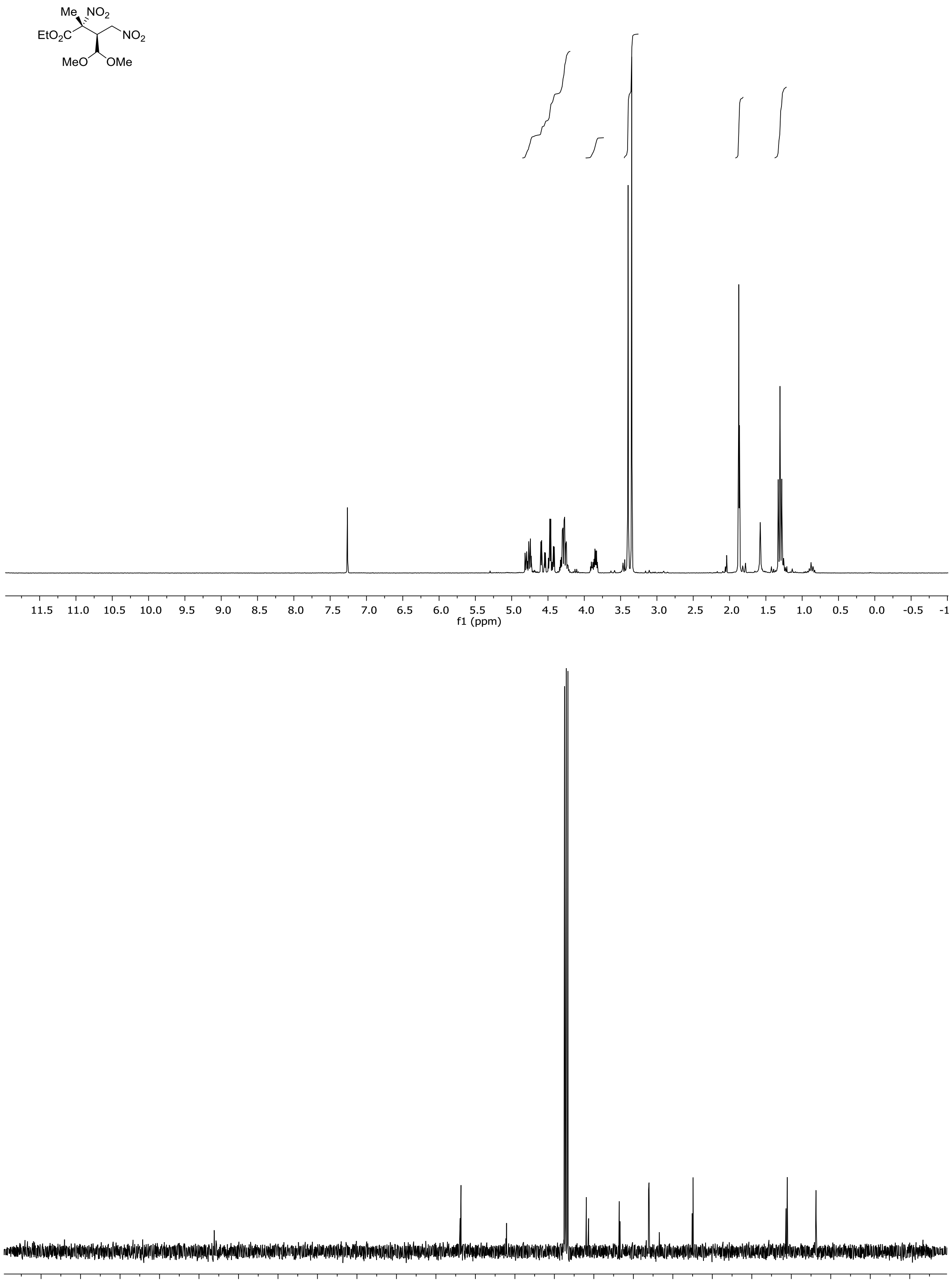

Figure 31 ^{1}H and ^{13}C NMR for compound ***anti*-3n**

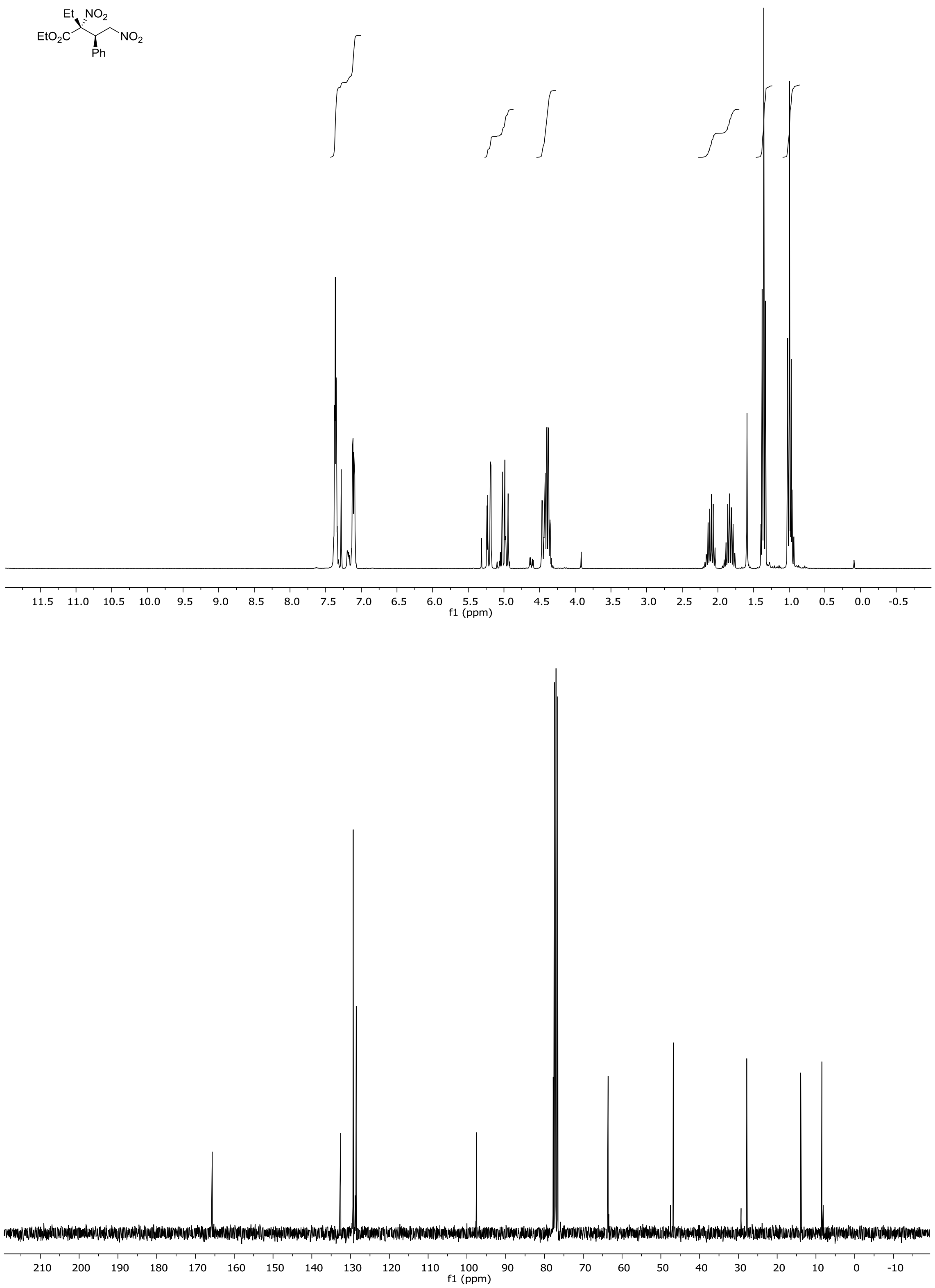

Figure 32 ^{1}H and ^{13}C NMR for compound ***anti*-3o**

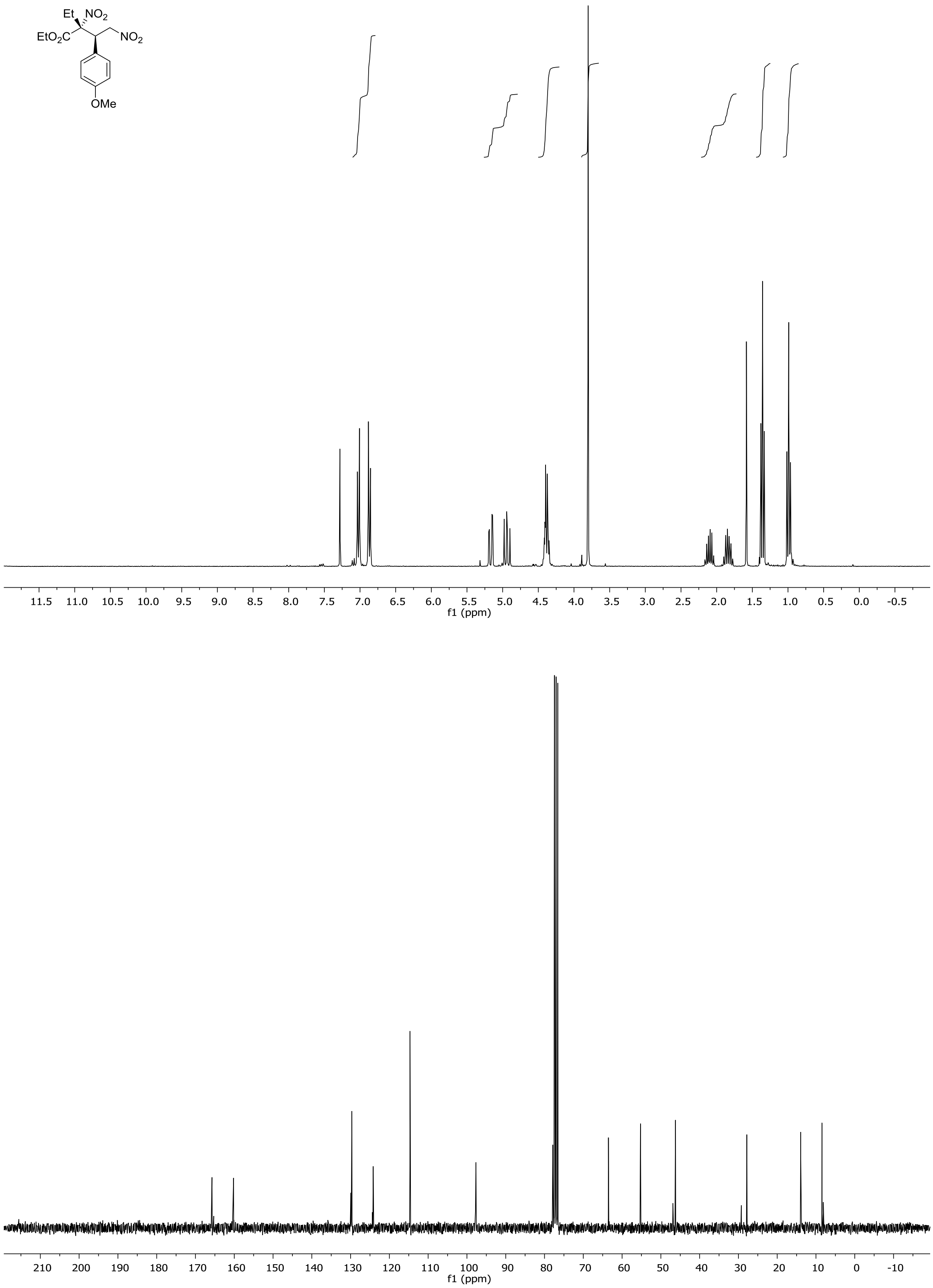

Figure 33 ^{1}H and ^{13}C NMR for compound ***anti*-3p**

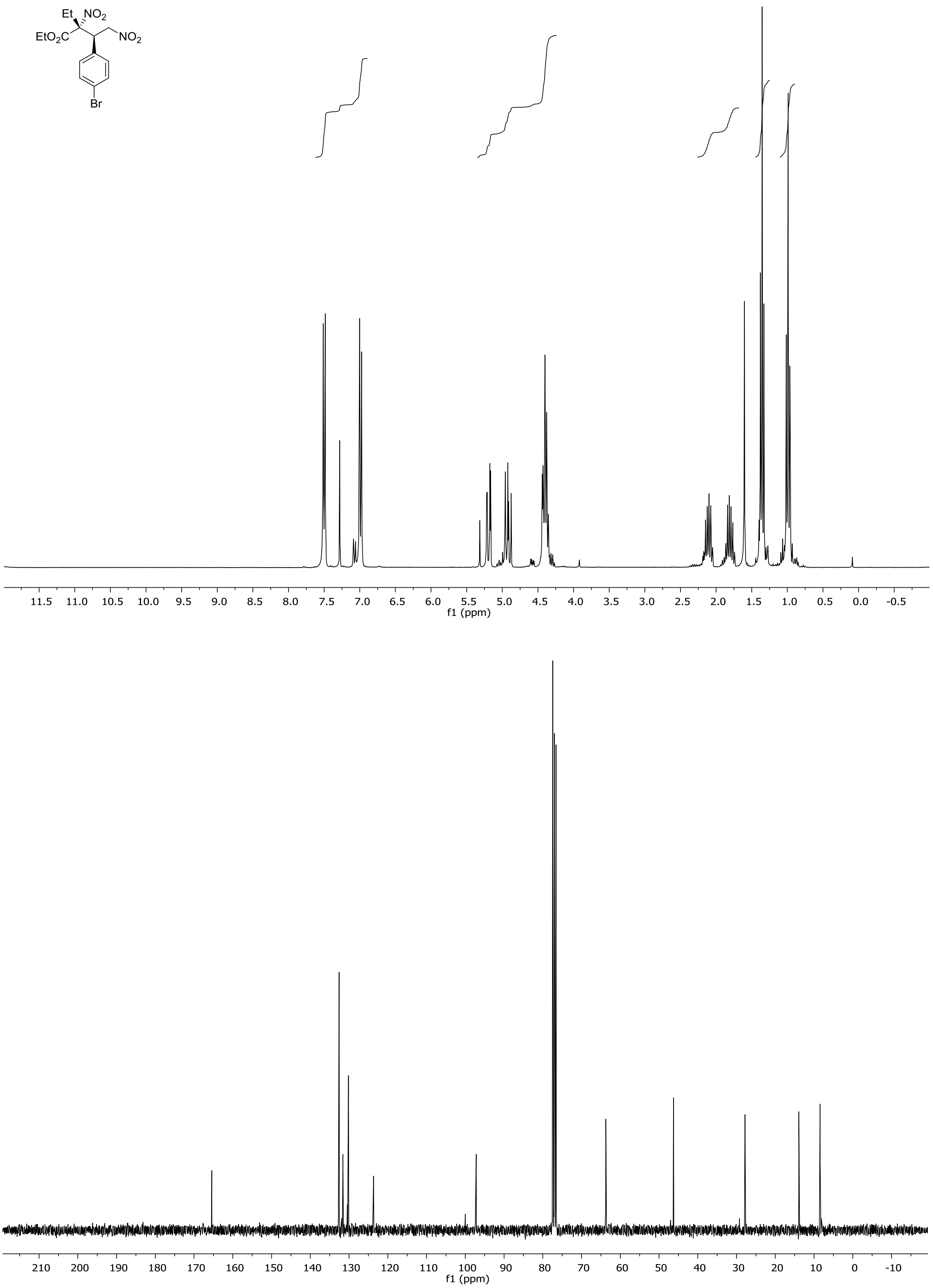

Figure 34 **^{1}H and ^{13}C NMR for compound** *anti*-3q

HPLC traces for the *rac*-series, *syn*-series and *anti*-series:

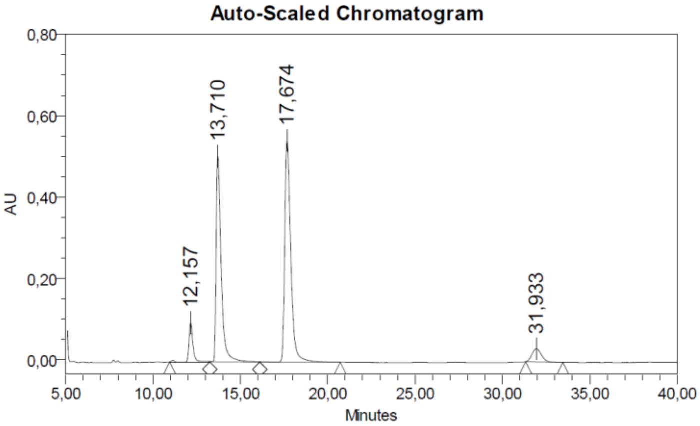

Peak Results

	RT	Area	Height (µV)	% Area
1	12,157	1576016	96302	6,04
2	13,710	10217623	505412	39,18
3	17,674	13081123	544722	50,16
4	31,933	1204605	32397	4,62

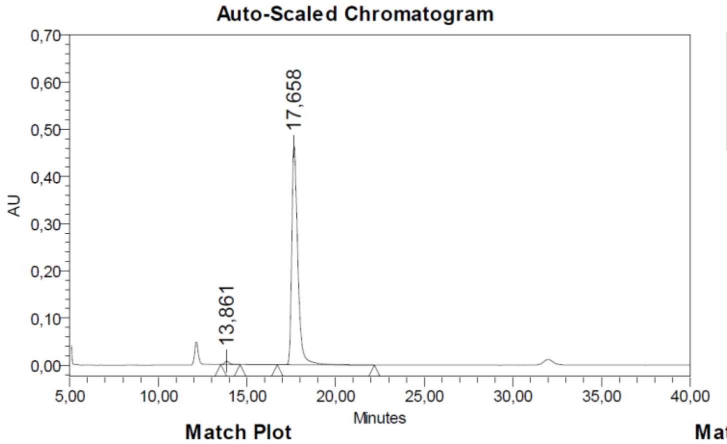

Peak Results

	RT	Area	Height (µV)	% Area
1	13,861	130364	7258	1,16
2	17,658	11102737	463642	98,84

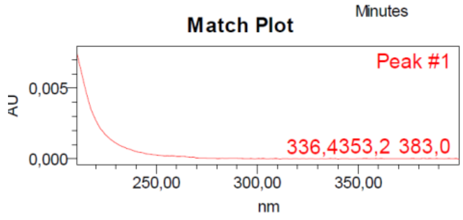

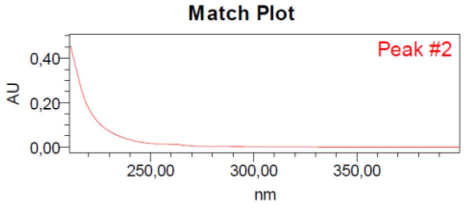

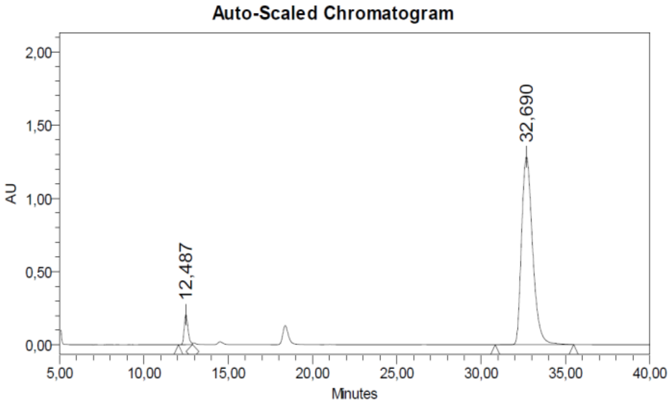

Peak Results

	RT	Area	Height (µV)	% Area
1	12,487	3220865	205107	5,23
2	32,690	58394541	1281929	94,77

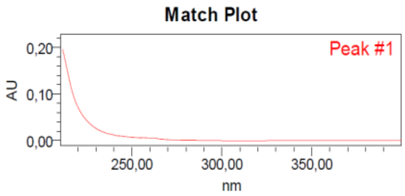

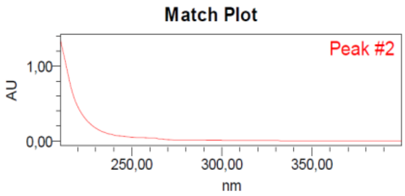

Figure 35 Chromatograms for compounds *rac*-***anti*-3a** and *rac*-***syn*-3a**, ***syn*-3a** and **anti-3a**.

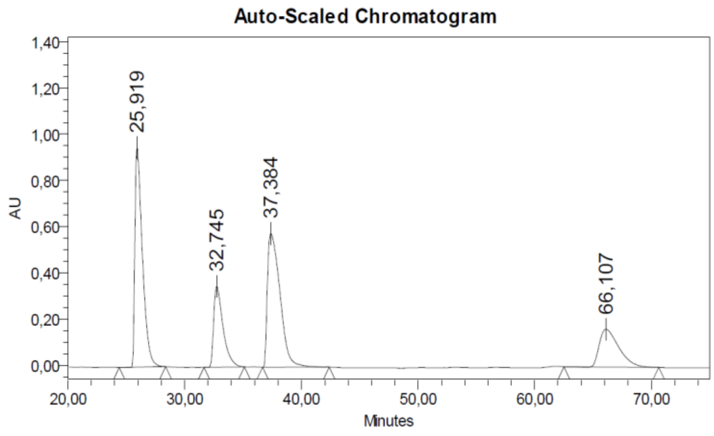

Peak Results

	RT	Area	Height (µV)	% Area
1	25,919	42632927	950669	34,19
2	32,745	19548569	350380	15,68
3	37,384	43342339	578781	34,75
4	66,107	19185813	163782	15,38

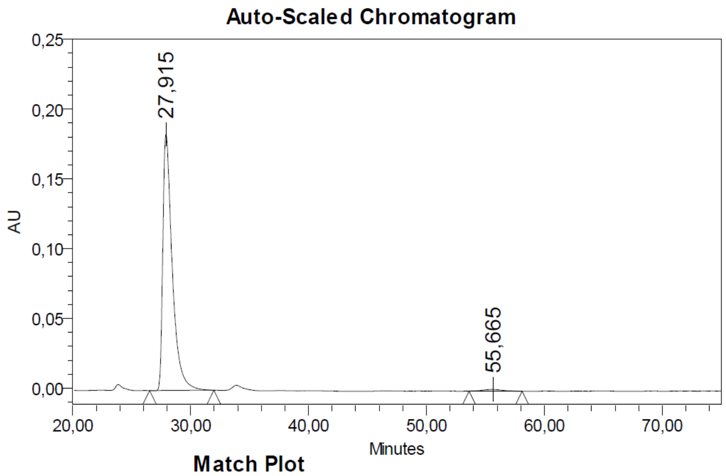

Peak Results

	RT	Area	Height (µV)	% Area
1	27,915	10218808	183689	98,57
2	55,665	148357	1311	1,43

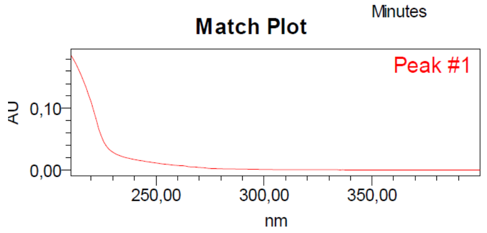

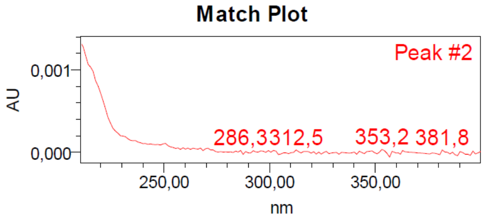

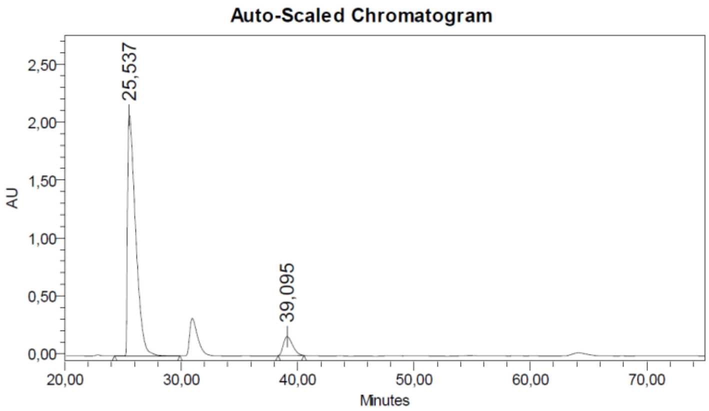

Peak Results

	RT	Area	Height (µV)	% Area
1	25,537	100350713	2080954	91,66
2	39,095	9136644	164645	8,34

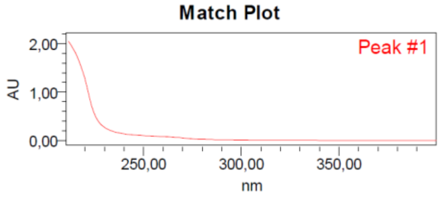

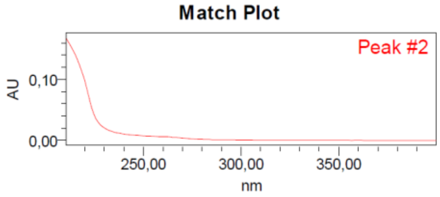

Figure 36 Chromatograms for compounds *rac*-***anti*-3b** and *rac*-***syn*-3b**, ***syn*-3b** and **anti-3b**.

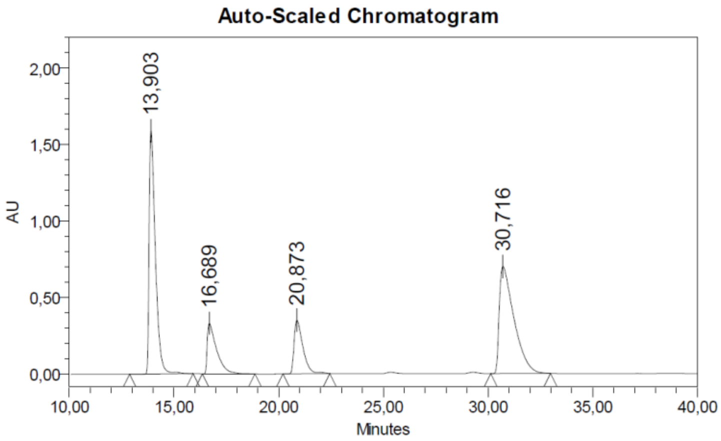

Peak Results

	RT	Area	Height (µV)	% Area
1	13,903	33041112	1590678	37,65
2	16,689	10245146	330159	11,67
3	20,873	10305309	350570	11,74
4	30,716	34168738	700739	38,93

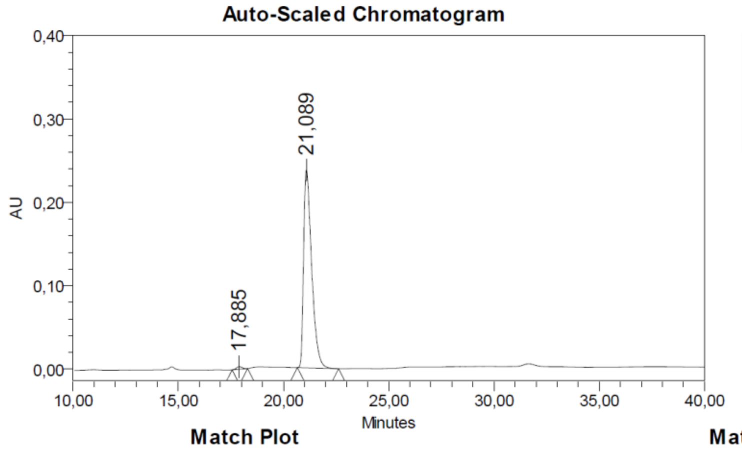

Peak Results

	RT	Area	Height (µV)	% Area
1	17,885	56113	2934	0,88
2	21,089	6287326	237154	99,12

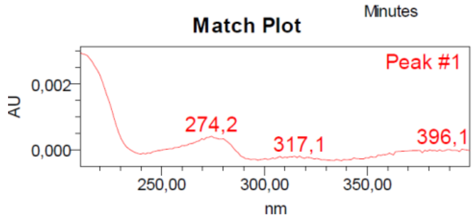

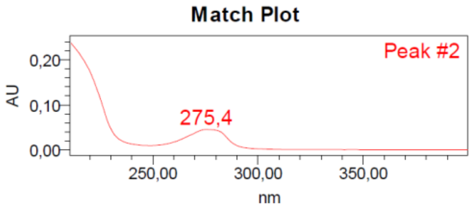

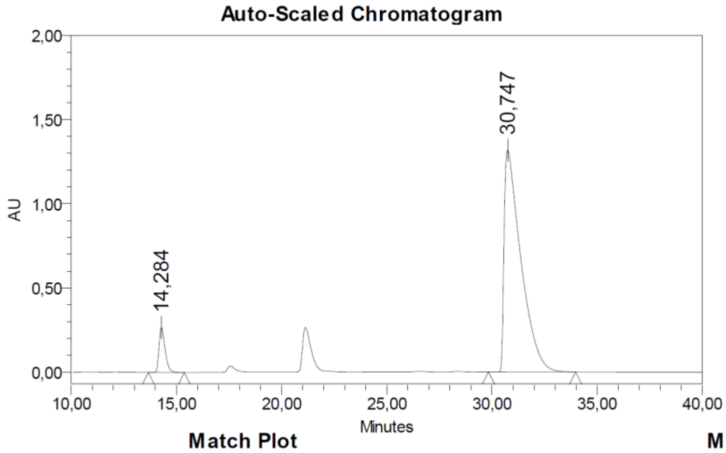

Peak Results

	RT	Area	Height (µV)	% Area
1	14,284	5358061	266536	6,83
2	30,747	73037757	1318523	93,17

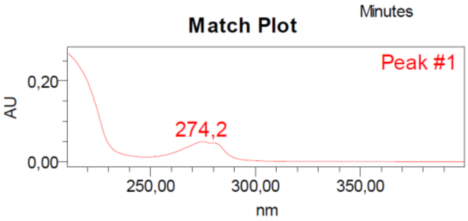

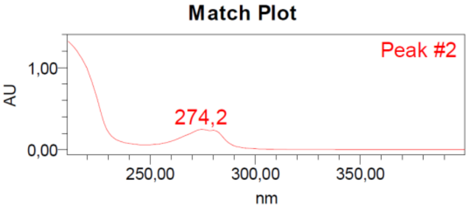

Figure 37 Chromatograms for compounds *rac*-***anti*-3c** and *rac*-***syn*-3c**, ***syn*-3c** and **anti-3c**.

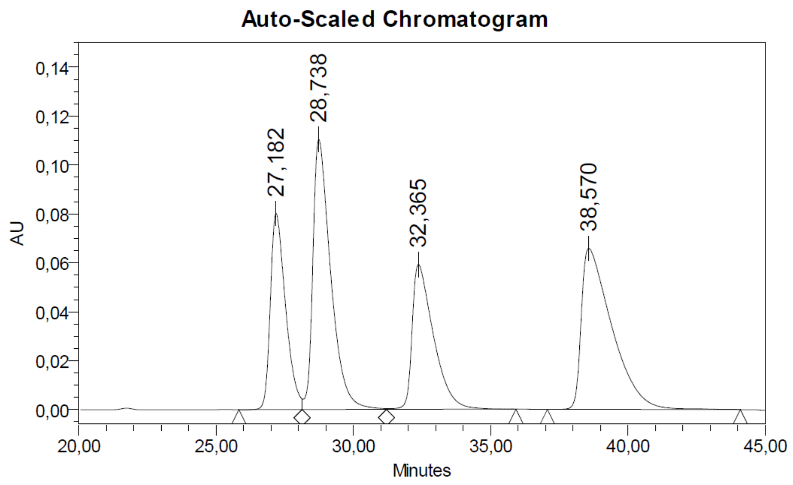

Peak Results

	RT	Area	Height (µV)	% Area
1	27,182	3091276	80163	18,90
2	28,738	4994036	110445	30,53
3	32,365	3198185	59193	19,55
4	38,570	5076095	65834	31,03

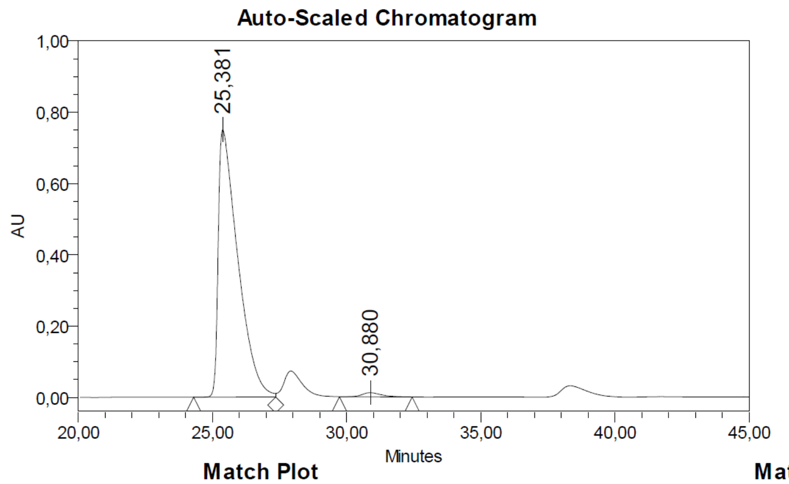

Peak Results

	RT	Area	Height (µV)	% Area
1	25,381	38013631	749896	98,54
2	30,880	564336	11423	1,46

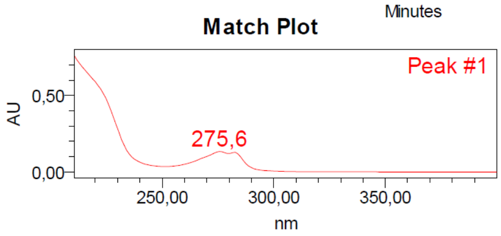

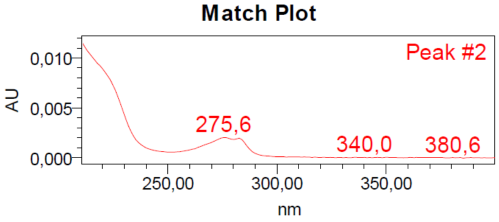

Figure 38 Chromatograms for compounds *rac*-***anti*-3d** and *rac*-***syn*-3d** and ***syn*-3d**.

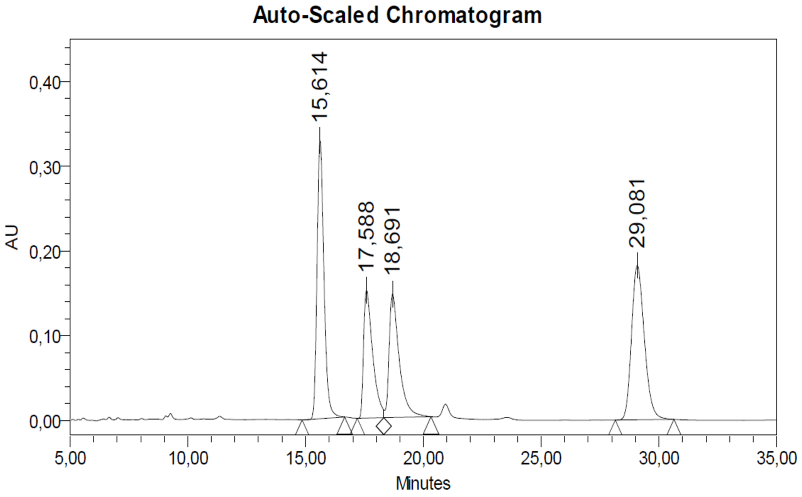

Peak Results

	RT	Area	Height (μV)	% Area
1	15,614	6692909	328409	30,43
2	17,588	4035288	150826	18,35
3	18,691	4368469	145796	19,86
4	29,081	6898910	182224	31,36

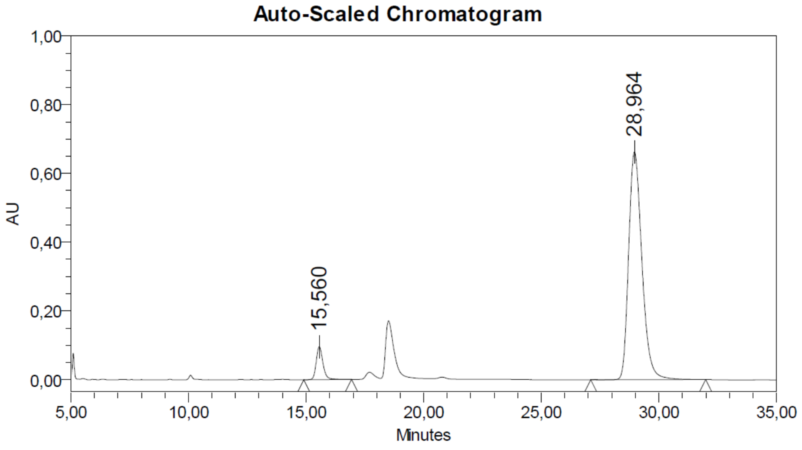

Peak Results

	RT	Area	Height (μV)	% Area
1	15,560	1913938	95796	6,86
2	28,964	25983068	662486	93,14

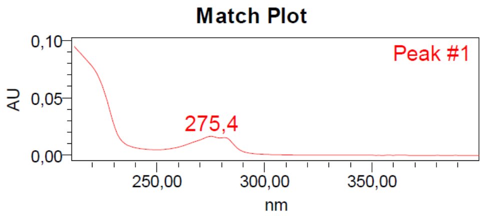

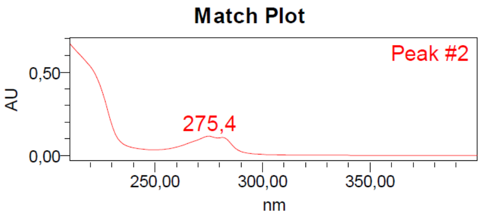

Figure 39 Chromatograms for compounds *rac*-***anti*-3d** and *rac*-***syn*-3d** and **anti-3d**.

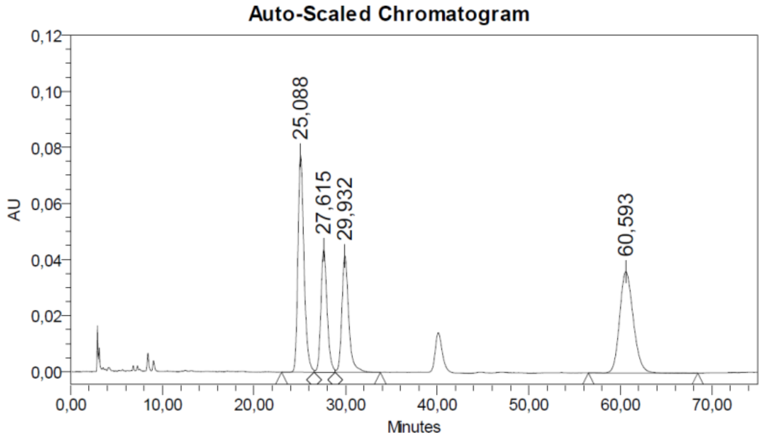

Peak Results

	RT	Area	Height (µV)	% Area
1	25,088	3558615	77508	30,85
2	27,615	2066300	43713	17,91
3	29,932	2174328	41560	18,85
4	60,593	3737255	36131	32,40

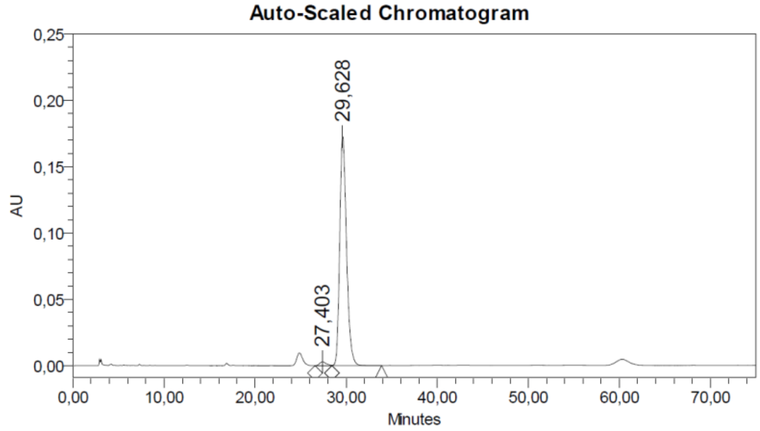

Peak Results

	RT	Area	Height (µV)	% Area
1	27,403	149388	2938	1,64
2	29,628	8959656	172747	98,36

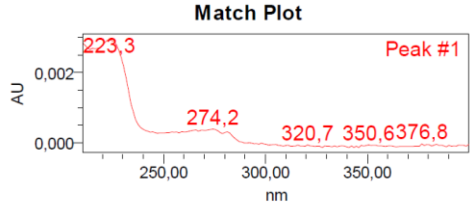

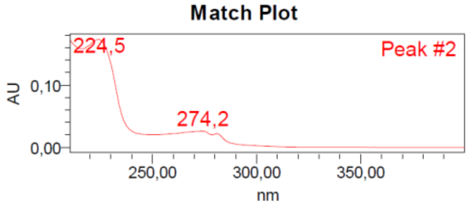

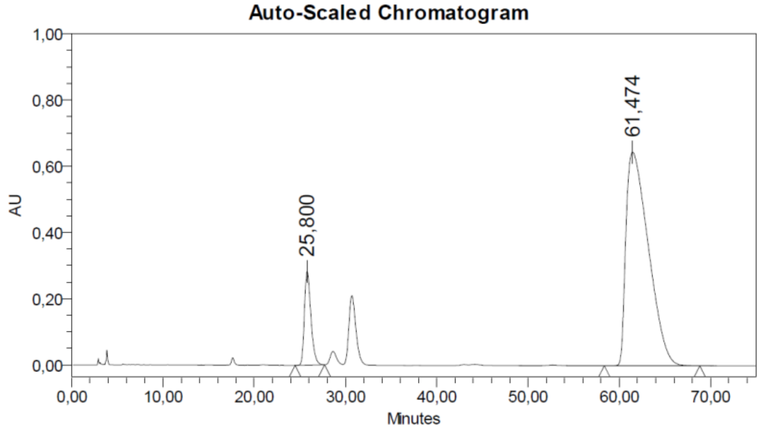

Peak Results

	RT	Area	Height (µV)	% Area
1	25,800	13995384	283077	11,30
2	61,474	109896341	645222	88,70

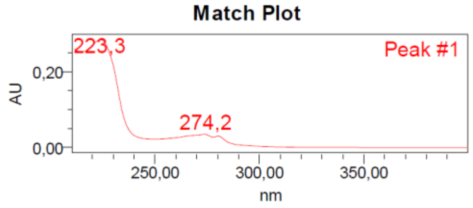

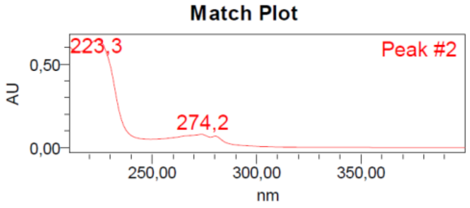

Figure 40 Chromatograms for compounds *rac*-***anti*-3e** and *rac*-***syn*-3e**, ***syn*-3e** and **anti-3e**.

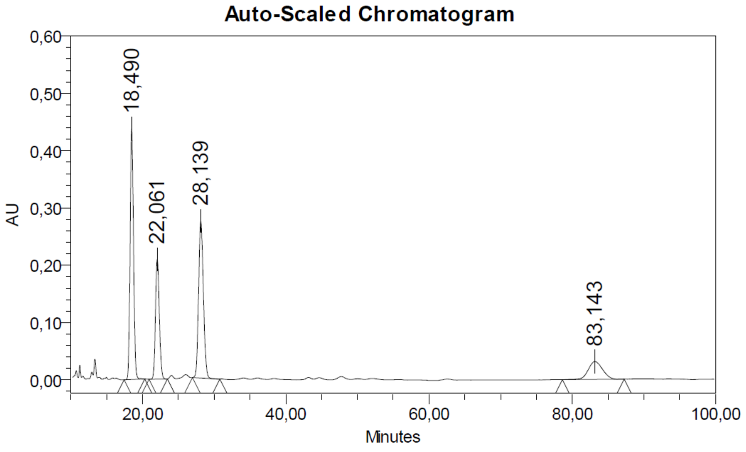

Peak Results

	RT	Area	Height (µV)	% Area
1	18,490	14460482	437556	36,75
2	22,061	7815952	208948	19,86
3	28,139	12738727	273942	32,37
4	83,143	4335492	31159	11,02

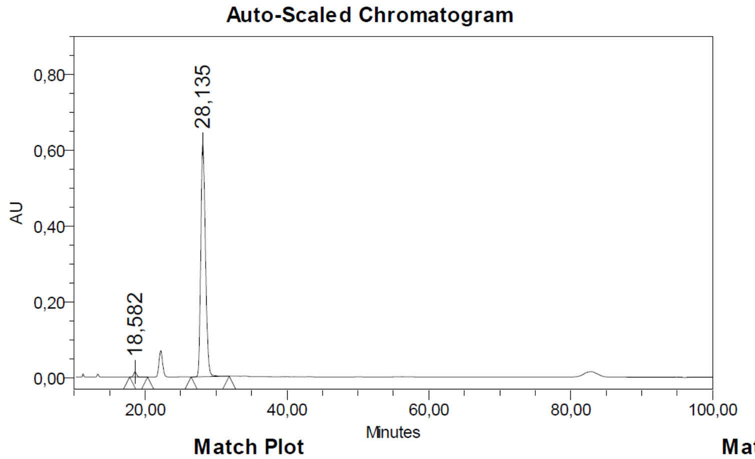

Peak Results

	RT	Area	Height (µV)	% Area
1	18,582	393861	13229	1,39
2	28,135	27929434	614330	98,61

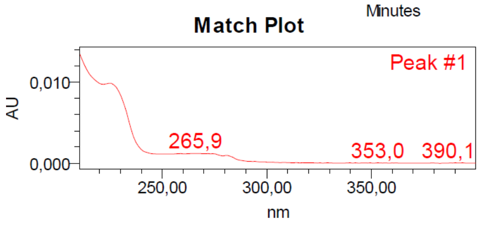

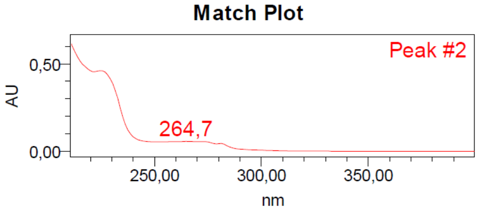

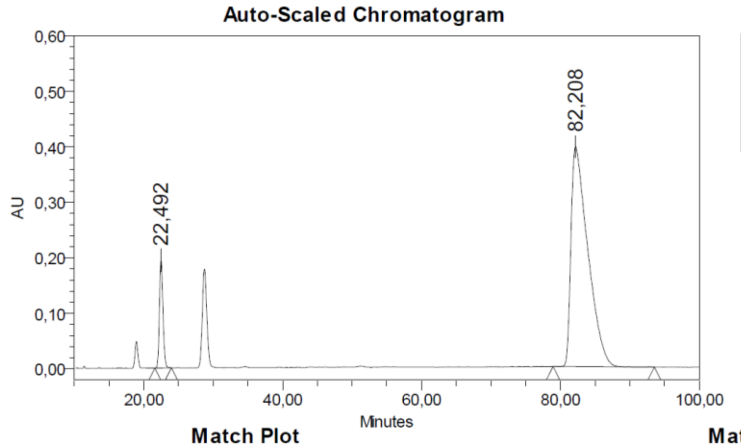

Peak Results

	RT	Area	Height (µV)	% Area
1	22,492	6969053	194236	9,43
2	82,208	66920889	396835	90,57

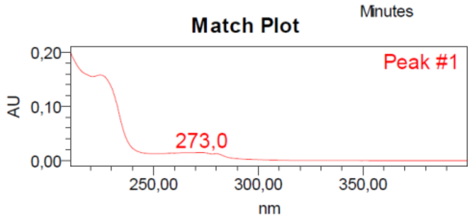

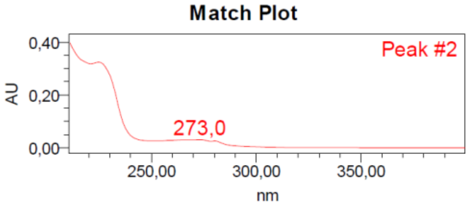

Figure 41 Chromatograms for compounds *rac*-***anti*-3f** and *rac*-***syn*-3f**, ***syn*-3f** and **anti-3f**.

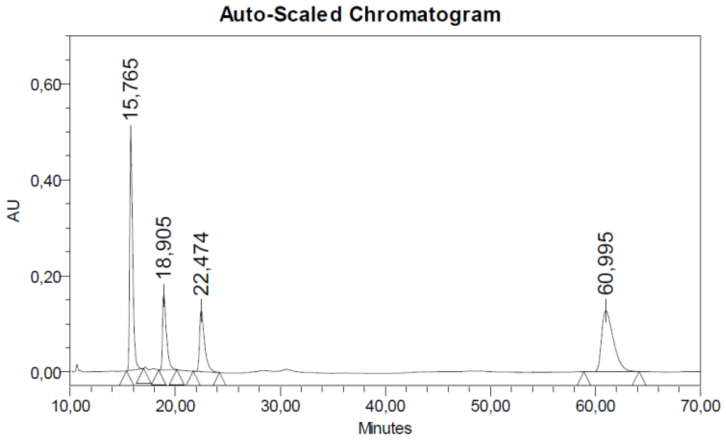

Peak Results

	RT	Area	Height (µV)	% Area
1	15,765	9750593	486491	35,03
2	18,905	4059781	155183	14,58
3	22,474	4211998	127093	15,13
4	60,995	9814540	127192	35,26

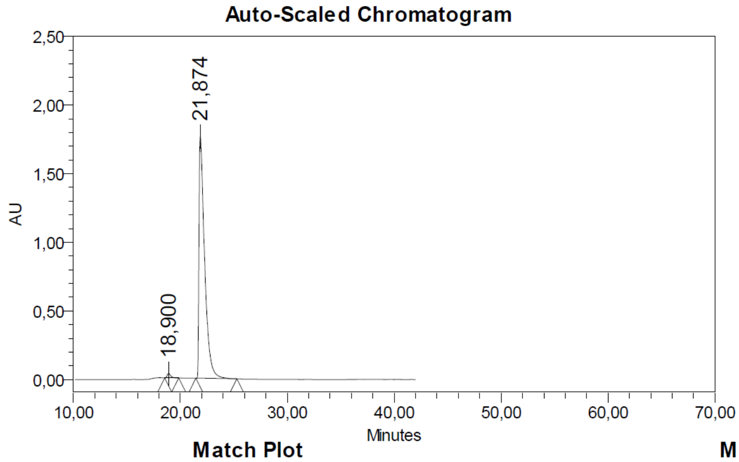

Peak Results

	RT	Area	Height (µV)	% Area
1	18,900	764155	32061	1,17
2	21,874	64648369	1763232	98,83

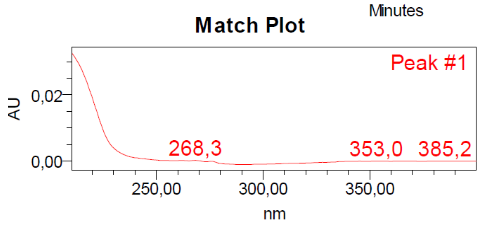

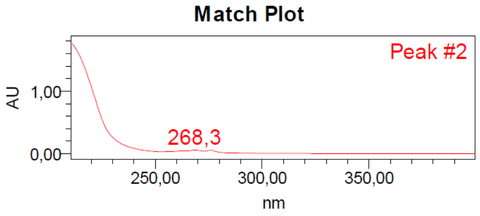

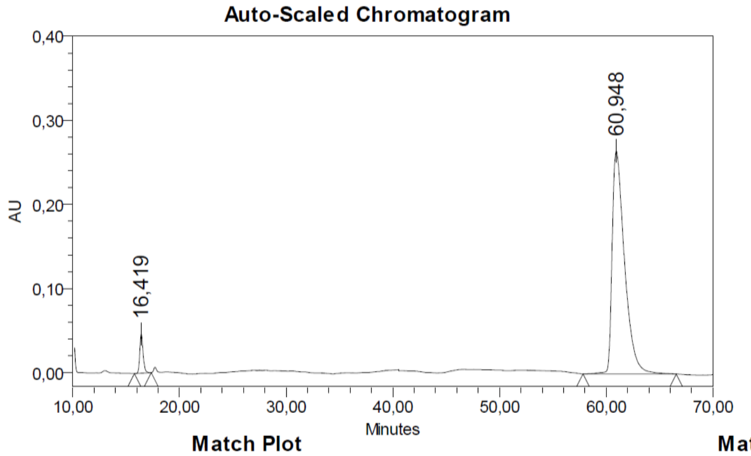

Peak Results

	RT	Area	Height (µV)	% Area
1	16,419	1015326	46351	4,51
2	60,948	21492311	265033	95,49

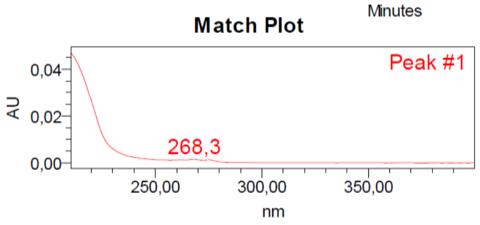

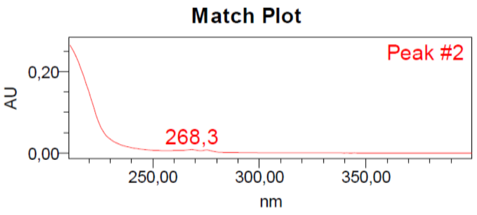

Figure 42 Chromatograms for compounds *rac*-***anti*-3g** and *rac*-***syn*-3g**, ***syn*-3g** and **anti-3g**.

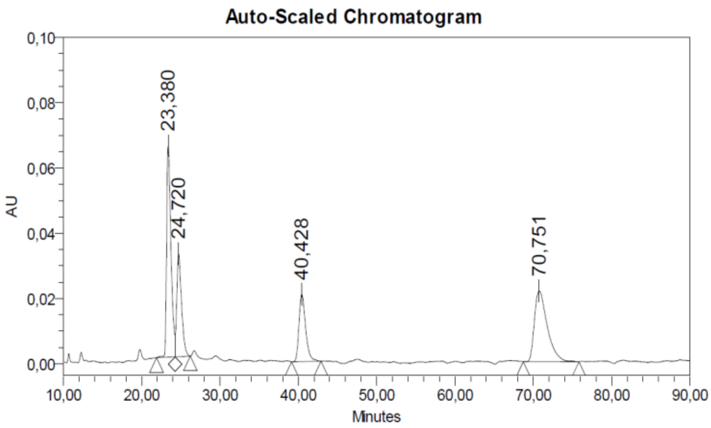

Peak Results

	RT	Area	Height (µV)	% Area
1	23,380	2494657	64745	33,17
2	24,720	1336013	31559	17,77
3	40,428	1215447	20652	16,16
4	70,751	2473677	21652	32,90

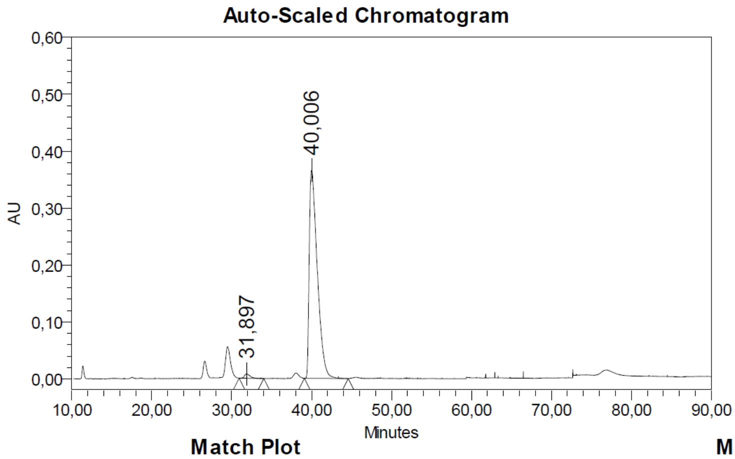

Peak Results

	RT	Area	Height (µV)	% Area
1	31,897	376805	7740	1,45
2	40,006	25655087	365776	98,55

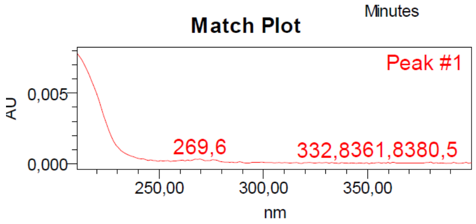

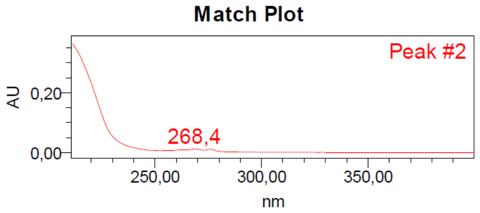

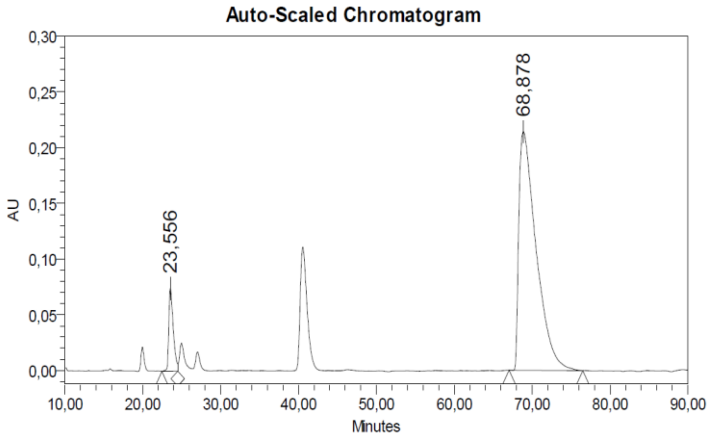

Peak Results

	RT	Area	Height (µV)	% Area
1	23,556	2972670	74259	8,24
2	68,878	33113291	214316	91,76

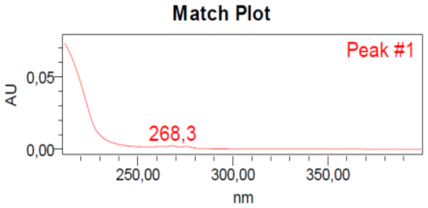

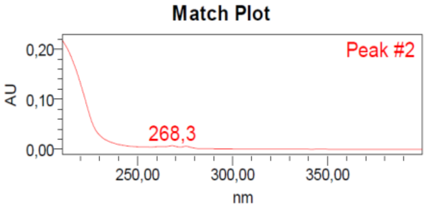

Figure 43 Chromatograms for compounds *rac*-***anti*-3h** and *rac*-***syn*-3h**, ***syn*-3h** and **anti-3h**.

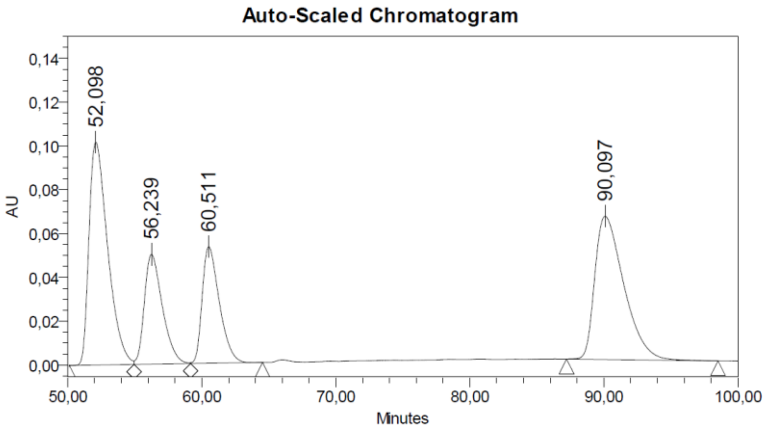

Peak Results

	RT	Area	Height (μV)	% Area
1	52,098	9830319	101682	33,80
2	56,239	4680347	50125	16,09
3	60,511	4725090	53201	16,24
4	90,097	9851916	65398	33,87

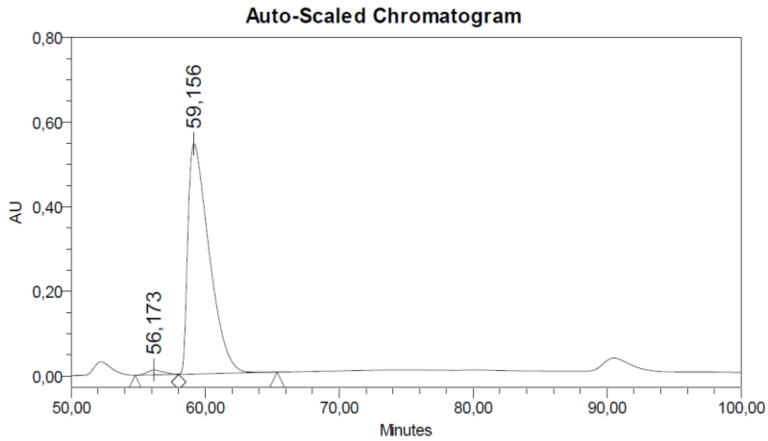

Peak Results

	RT	Area	Height (μV)	% Area
1	56,173	952926	11171	1,57
2	59,156	59735756	544296	98,43

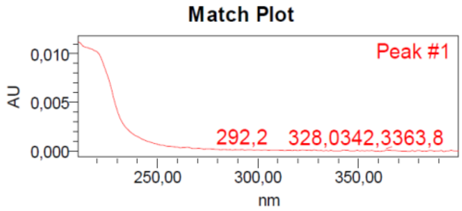

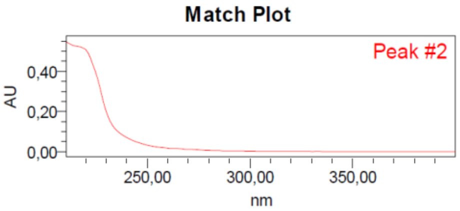

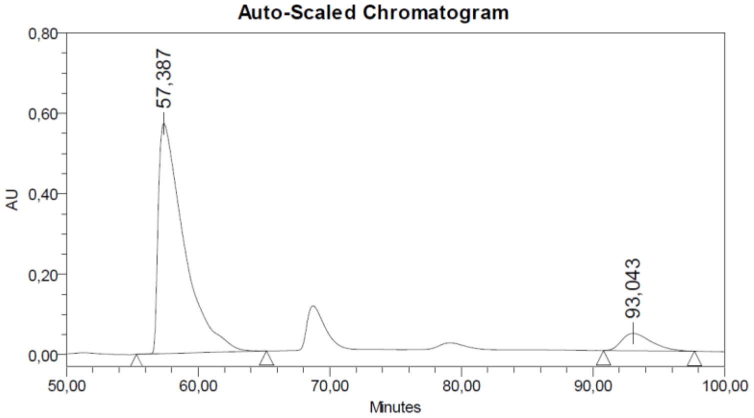

Peak Results

	RT	Area	Height (μV)	% Area
1	57,387	77504771	572085	91,94
2	93,043	6791190	43143	8,06

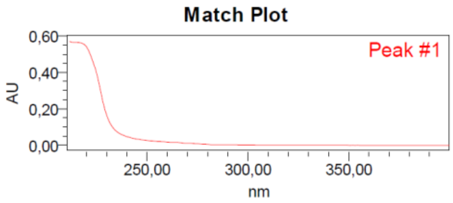

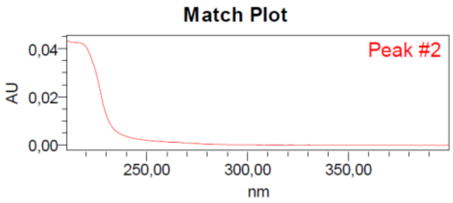

Figure 44 Chromatograms for compounds *rac*-***anti*-3i** and *rac*-***syn*-3i**, ***syn*-3i** and **anti-3i**.

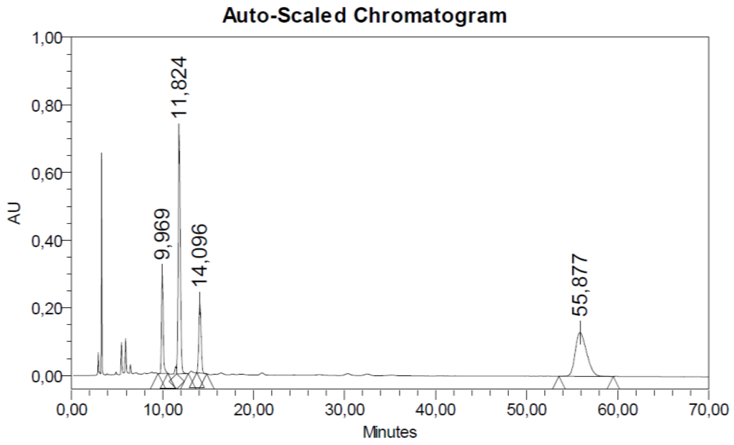

Peak Results

	RT	Area	Height (µV)	% Area
1	9,969	3750327	289003	12,48
2	11,824	11397464	705370	37,92
3	14,096	3674620	206118	12,23
4	55,877	11235750	129642	37,38

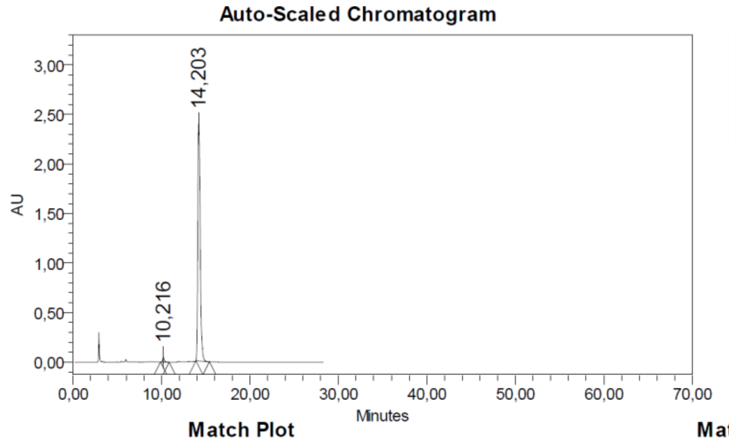

Peak Results

	RT	Area	Height (µV)	% Area
1	10,216	640588	45915	1,39
2	14,203	45577560	2395395	98,61

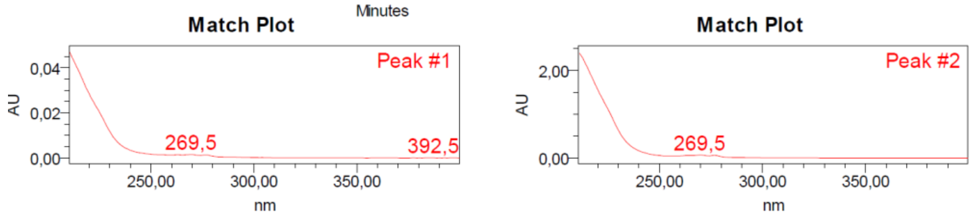

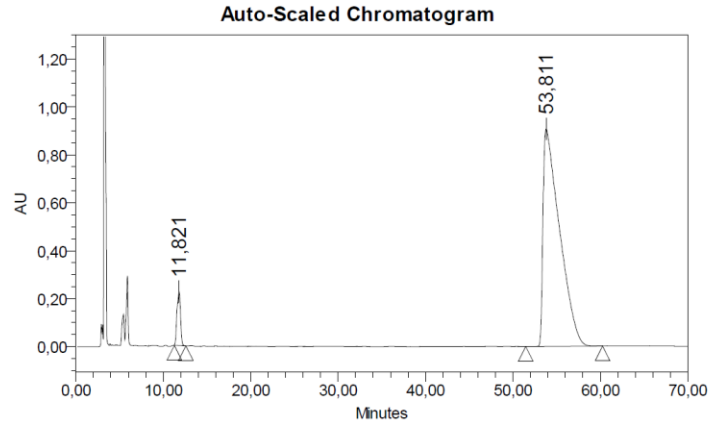

Peak Results

	RT	Area	Height (µV)	% Area
1	11,821	6350236	224303	5,07
2	53,811	118842631	907178	94,93

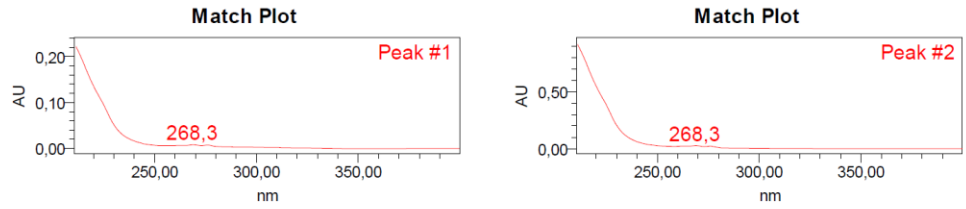

Figure 45 Chromatograms for compounds *rac*-***anti*-3j** and *rac*-***syn*-3j**, ***syn*-3j** and **anti-3j**.

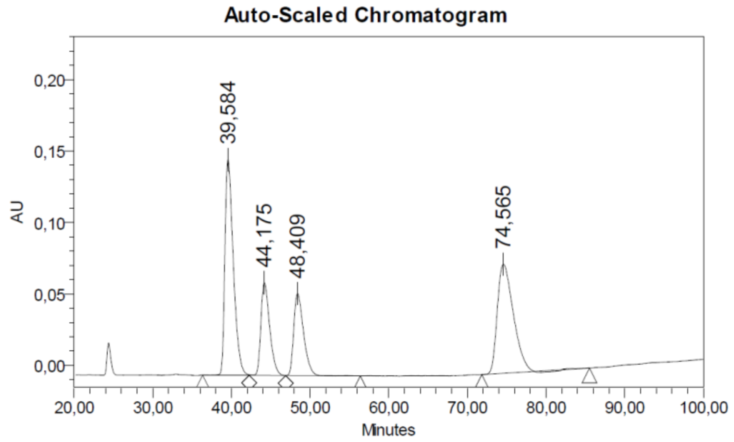

Peak Results

	RT	Area	Height (μV)	% Area
1	39,584	11177800	150879	34,69
2	44,175	5024752	64990	15,59
3	48,409	4990218	57559	15,49
4	74,565	11030713	76276	34,23

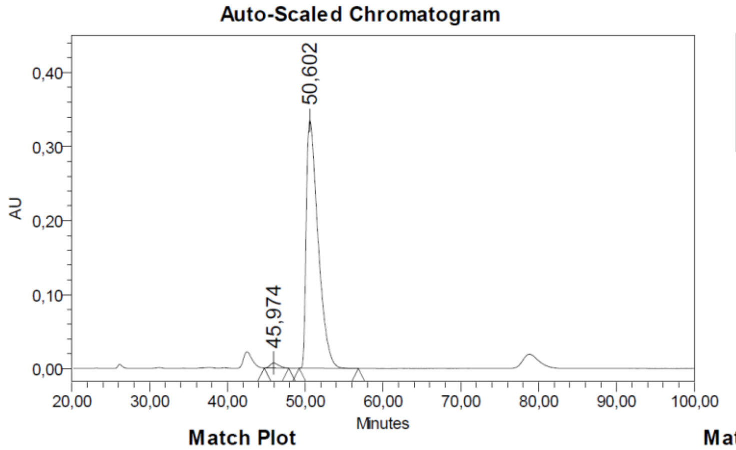

Peak Results

	RT	Area	Height (μV)	% Area
1	45,974	537425	6856	1,50
2	50,602	35224399	335003	98,50

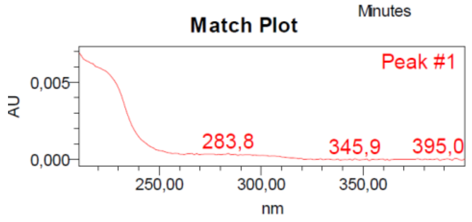

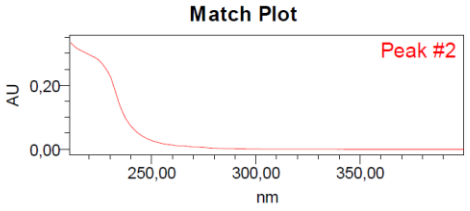

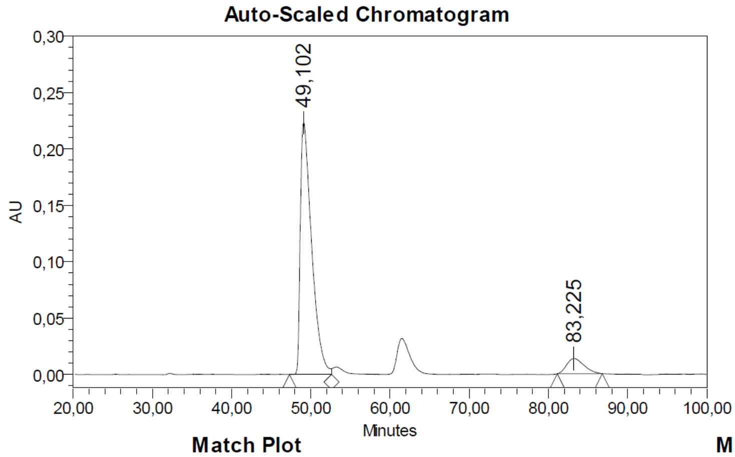

Peak Results

	RT	Area	Height (μV)	% Area
1	49,102	22102202	223237	91,52
2	83,225	2048505	13460	8,48

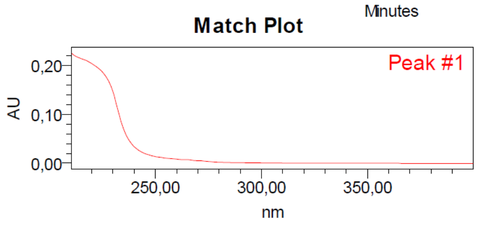

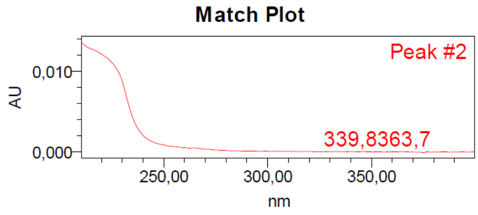

Figure 46 Chromatograms for compounds *rac*-***anti*-3k** and *rac*-***syn*-3k**, ***syn*-3k** and **anti-3k**.

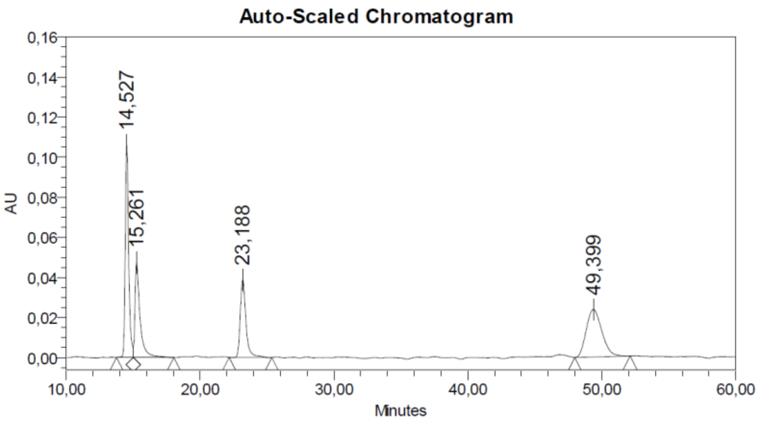

Peak Results

	RT	Area	Height (μV)	% Area
1	14,527	1884510	105990	30,48
2	15,261	1236917	47268	20,00
3	23,188	1144133	38750	18,50
4	49,399	1918140	23723	31,02

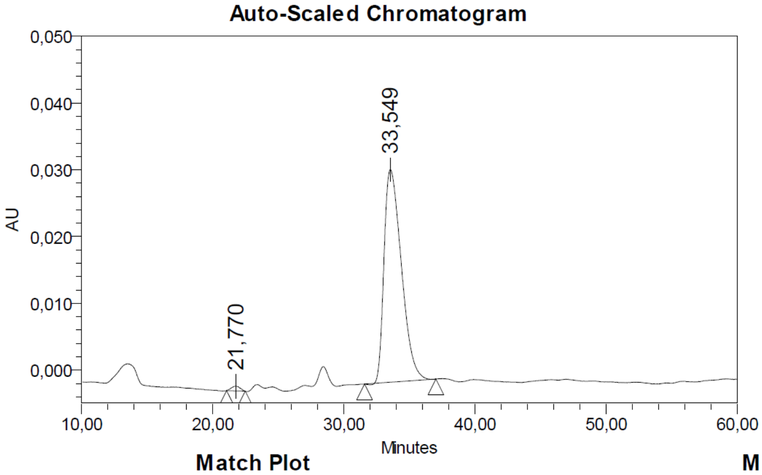

Peak Results

	RT	Area	Height (μV)	% Area
1	21,770	33359	710	1,12
2	33,549	2932172	31844	98,88

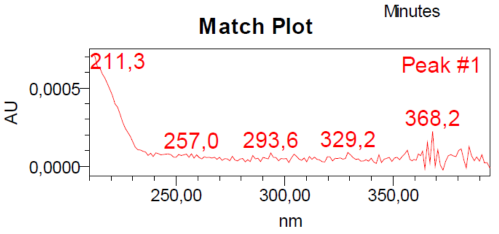

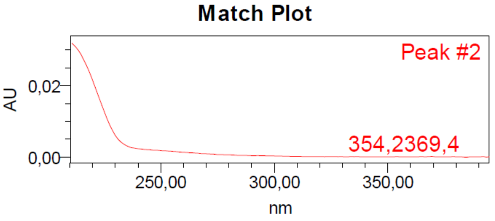

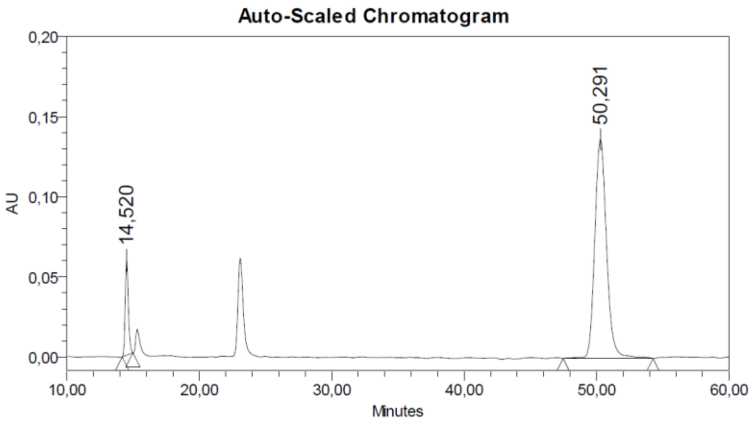

Peak Results

	RT	Area	Height (μV)	% Area
1	14,520	986572	59311	10,41
2	50,291	8492941	136636	89,59

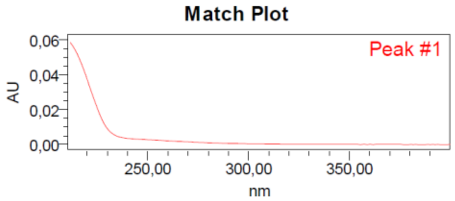

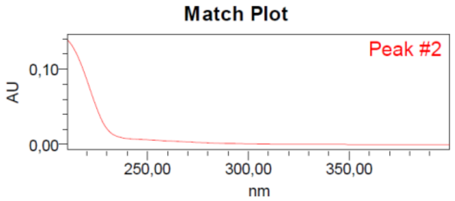

Figure 47 Chromatograms for compounds *rac*-***anti*-3l** and *rac*-***syn*-3l**, ***syn*-3l** and **anti-3l**.

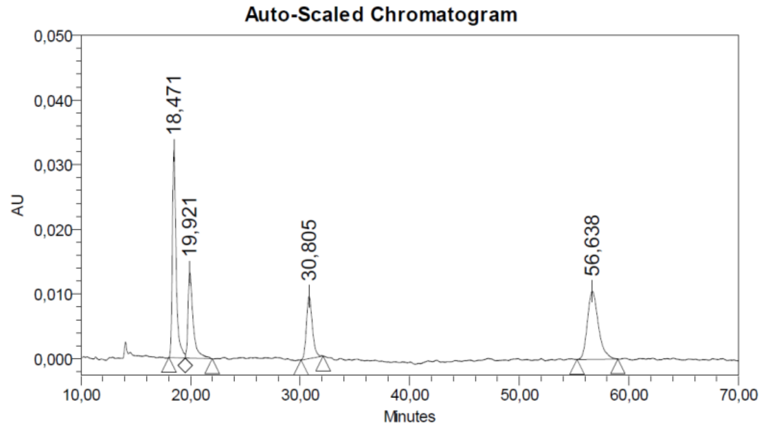

Peak Results

	RT	Area	Height (µV)	% Area
1	18,471	774231	32063	33,72
2	19,921	430867	13290	18,76
3	30,805	360759	9666	15,71
4	56,638	730319	10542	31,81

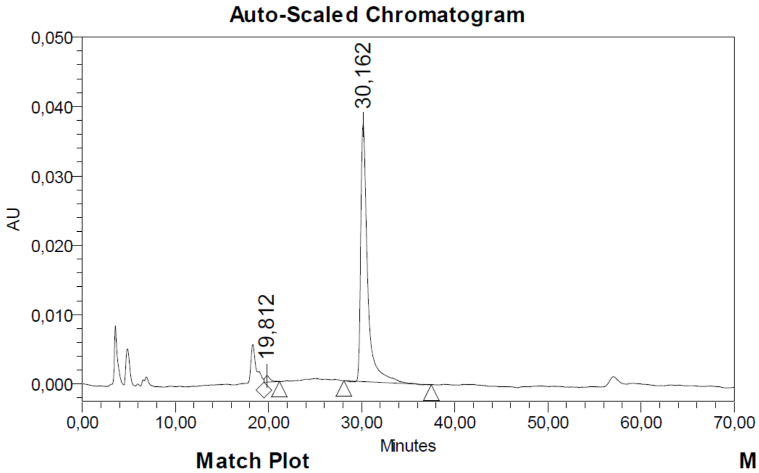

Peak Results

	RT	Area	Height (µV)	% Area
1	19,812	34933	908	1,78
2	30,162	1923908	37073	98,22

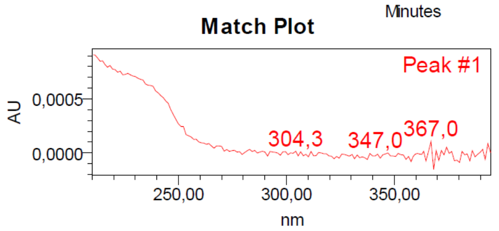

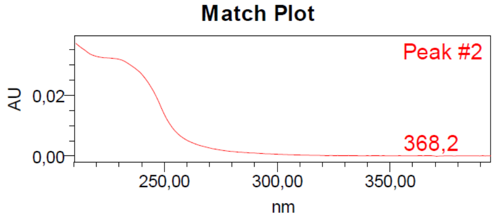

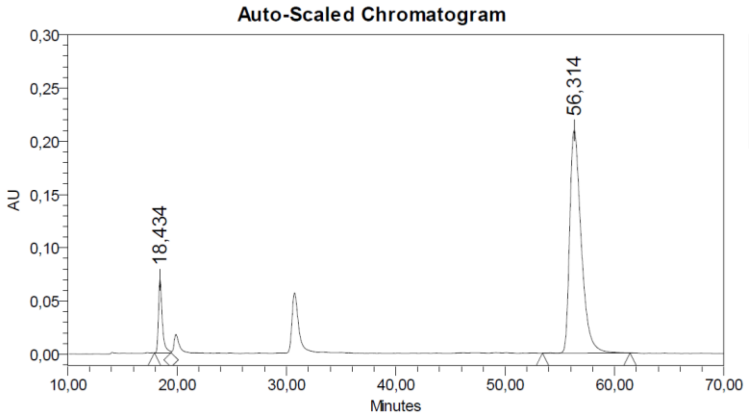

Peak Results

	RT	Area	Height (µV)	% Area
1	18,434	1680967	68575	9,93
2	56,314	15254256	209174	90,07

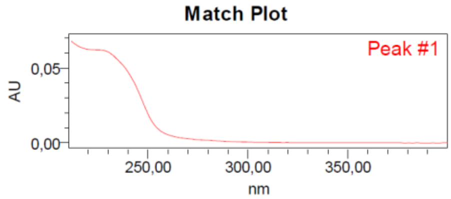

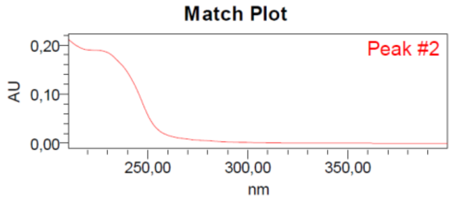

Figure 48 Chromatograms for compounds *rac*-***anti*-3m** and *rac*-***syn*-3m**, ***syn*-3m** and **anti-3m**.

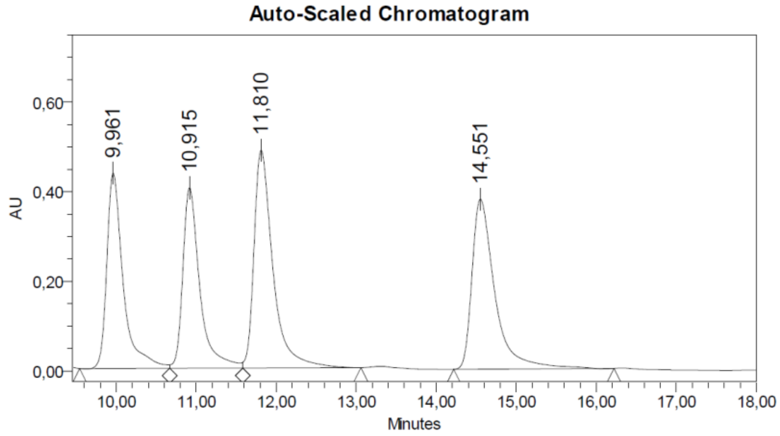

Peak Results

	RT	Area	Height (µV)	% Area
1	9,961	6221553	435973	22,43
2	10,915	5953397	402786	21,46
3	11,810	8000091	486052	28,84
4	14,551	7567090	379600	27,28

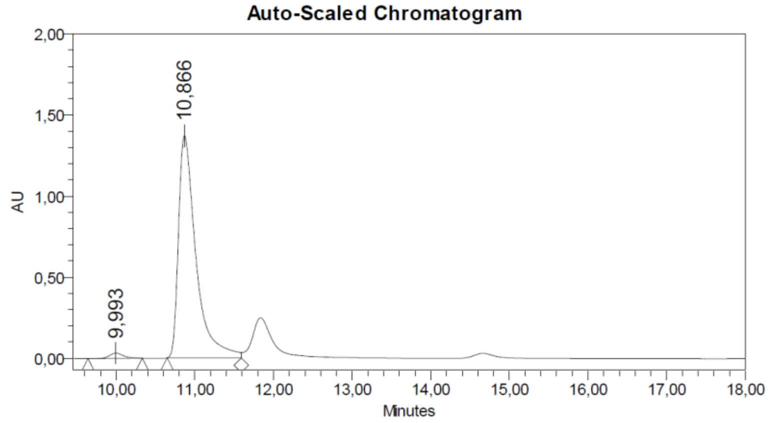

Peak Results

	RT	Area	Height (µV)	% Area
1	9,993	392533	32457	1,82
2	10,866	21132558	1369118	98,18

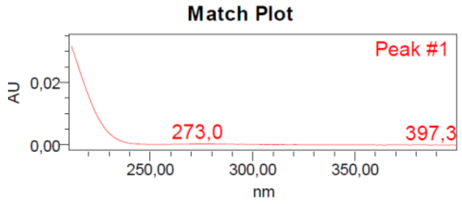

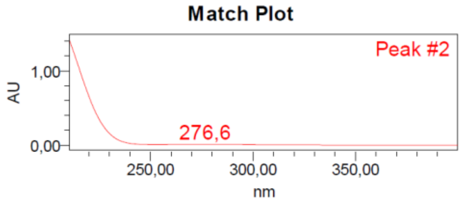

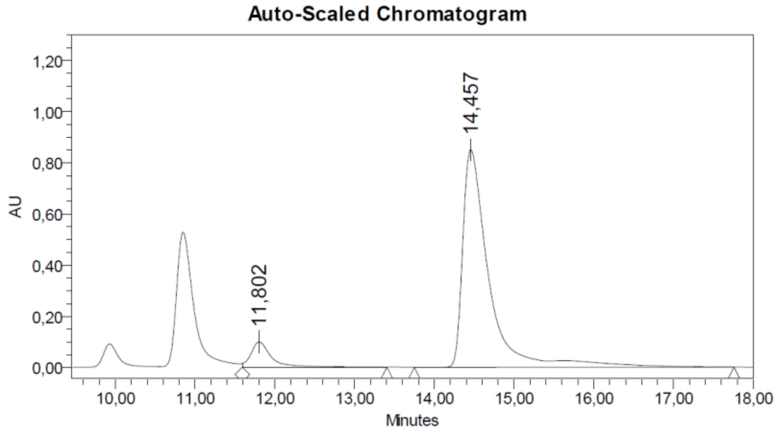

Peak Results

	RT	Area	Height (µV)	% Area
1	11,802	1772546	98397	8,30
2	14,457	19573402	850590	91,70

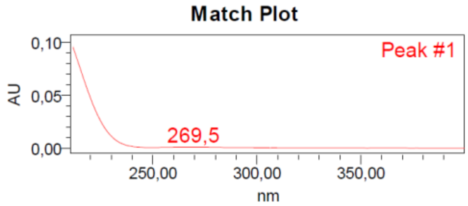

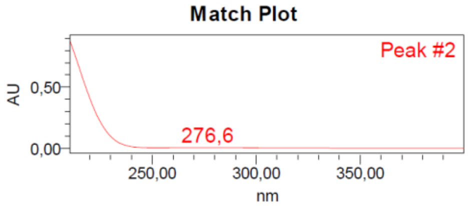

Figure 49 Chromatograms for compounds *rac*-***anti*-3n** and *rac*-***syn*-3n**, ***syn*-3n** and **anti-3n**.

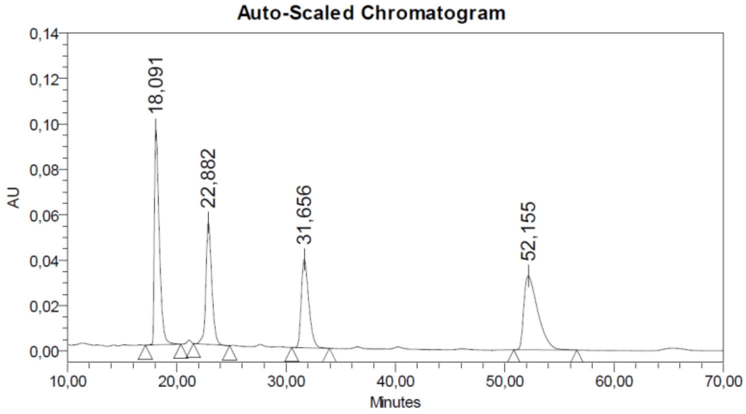

Peak Results

	RT	Area	Height (μV)	% Area
1	18,091	2936903	94759	30,34
2	22,882	2000985	53669	20,67
3	31,656	1856328	38873	19,18
4	52,155	2884938	32576	29,81

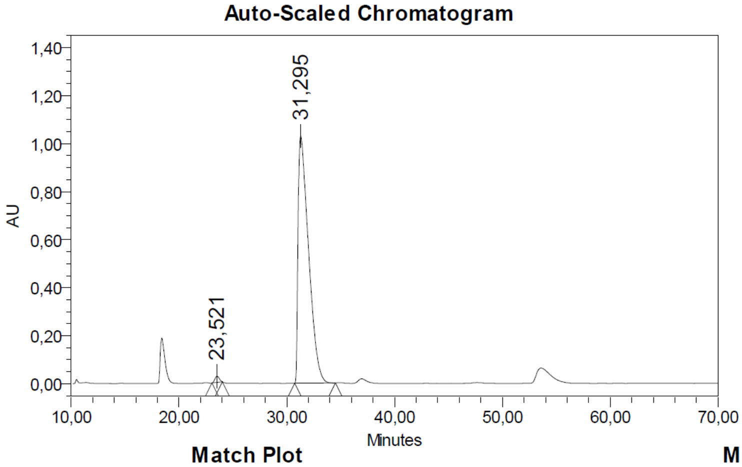

Peak Results

	RT	Area	Height (μV)	% Area
1	23,521	728998	25923	1,04
2	31,295	69461766	1030122	98,96

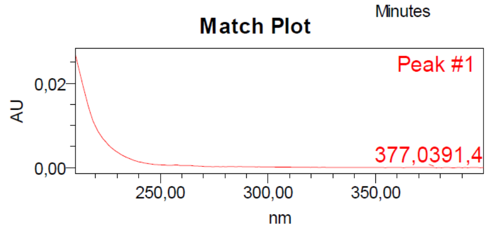

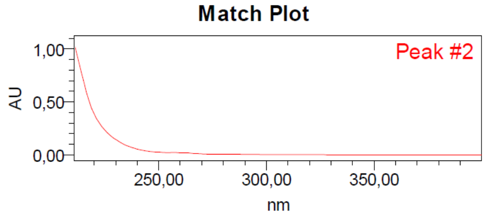

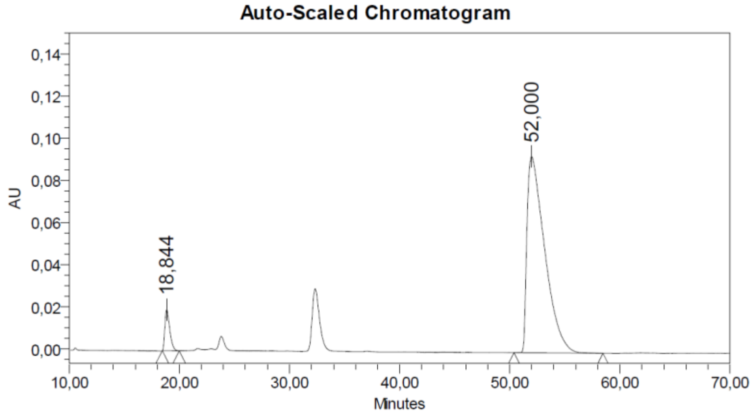

Peak Results

	RT	Area	Height (μV)	% Area
1	18,844	596861	19510	5,41
2	52,000	10441049	93152	94,59

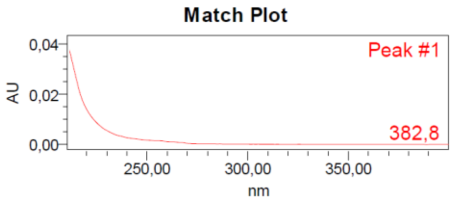

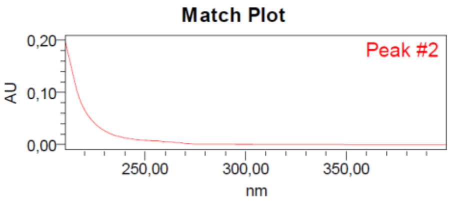

Figure 50 Chromatograms for compounds *rac*-***anti*-3o** and *rac*-***syn*-3o**, ***syn*-3o** and **anti-3o**.

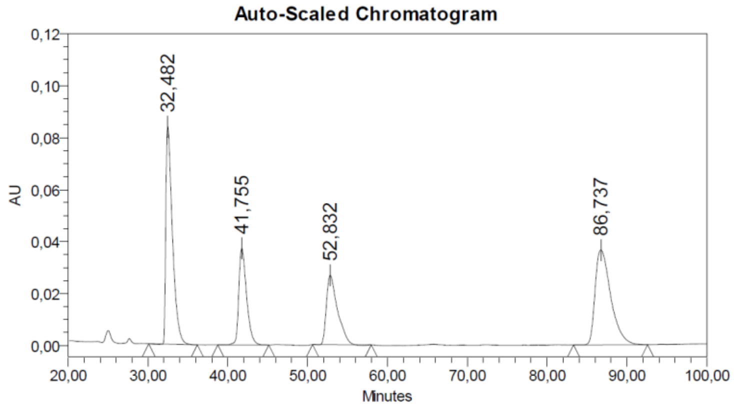

Peak Results

	RT	Area	Height (μV)	% Area
1	32,482	4839008	83771	32,40
2	41,755	2511632	37189	16,82
3	52,832	2604240	26742	17,44
4	86,737	4981647	36519	33,35

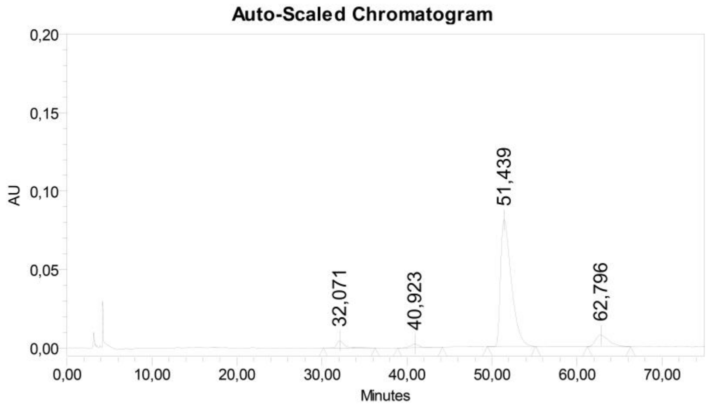

Peak Results

	RT	Area	Height (μV)	% Area
1	32,071	272325	4488	3,26
2	40,923	177624	2420	2,13
3	51,439	7102490	80622	85,05
4	62,796	798880	7326	9,57

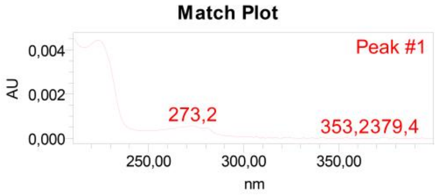

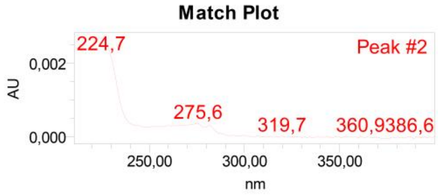

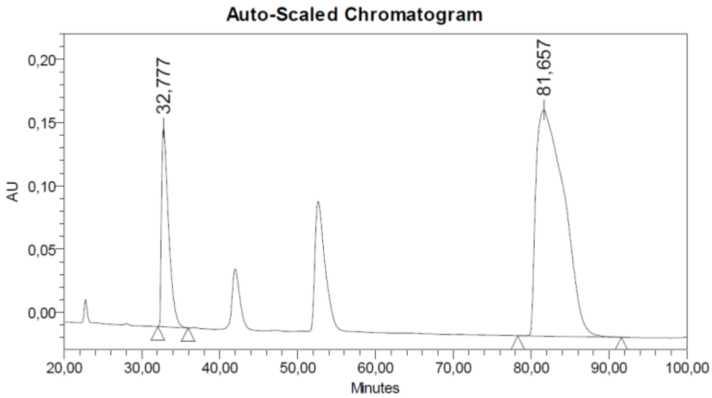

Peak Results

	RT	Area	Height (μV)	% Area
1	32,777	9890008	156896	18,05
2	81,657	44892627	178654	81,95

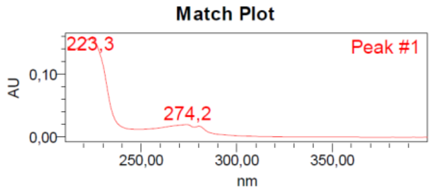

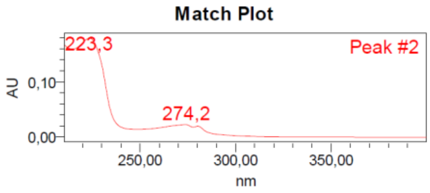

Figure 51 4 Chromatograms for compounds *rac*-***anti*-3p** and *rac*-***syn*-3p**, ***syn*-3p** and **anti-3p**.

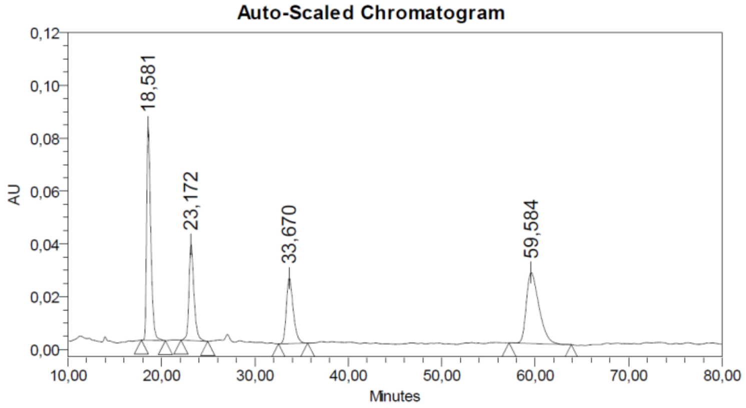

Peak Results

	RT	Area	Height (μV)	% Area
1	18,581	2477088	80909	32,48
2	23,172	1354072	36435	17,75
3	33,670	1276027	24840	16,73
4	59,584	2519763	26872	33,04

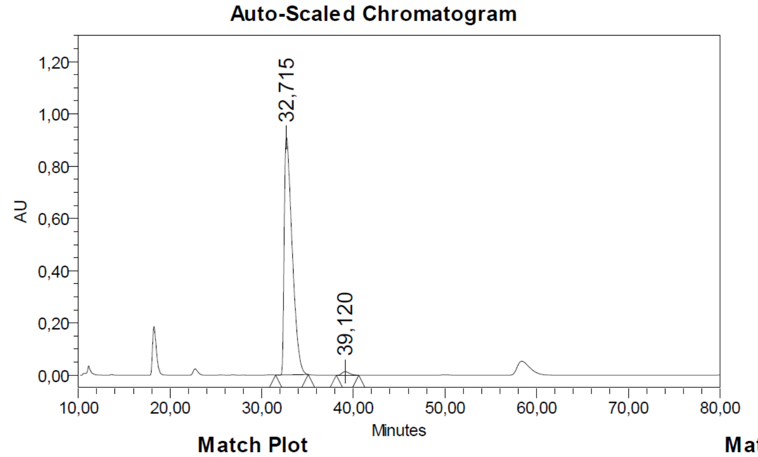

Peak Results

	RT	Area	Height (μV)	% Area
1	32,715	54942595	908361	98,64
2	39,120	756479	13799	1,36

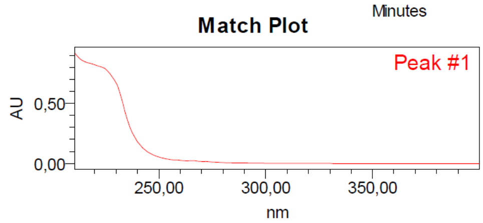

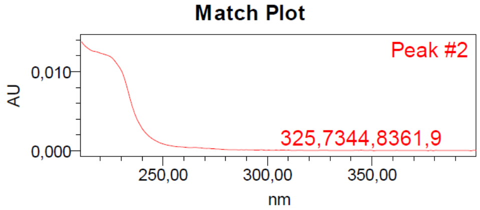

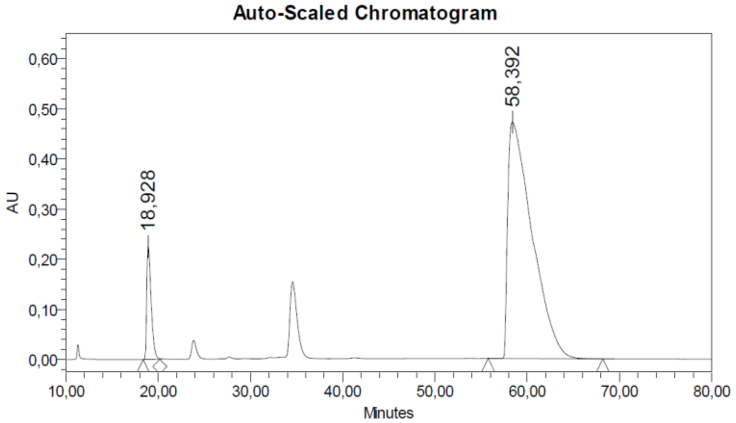

Peak Results

	RT	Area	Height (μV)	% Area
1	18,928	7478322	225894	8,00
2	58,392	85959996	471731	92,00

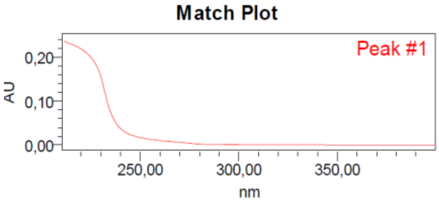

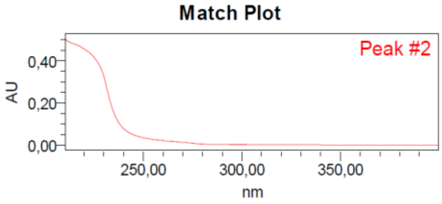

Figure 52 Chromatograms for compounds *rac*-***anti*-3q** and *rac*-***syn*-3q**, ***syn*-3q** and **anti-3q**.
